# The epidemiology and phenomenology of non-antipsychotic-induced dystonia: a hybrid systematic-narrative review

**DOI:** 10.1192/j.eurpsy.2025.18

**Published:** 2025-02-10

**Authors:** Kirsten Catthoor, Johan Detraux, Marc De Hert

**Affiliations:** 1 Ziekenhuis Netwerk Antwerpen (ZNA), Antwerp, Belgium; 2 Flemish Psychiatric Association, Kortenberg, Belgium; 3 Collaborative Antwerp Psychiatric Research Institute (CAPRI), University of Antwerp, Antwerp, Belgium; 4 University Psychiatric Center KU Leuven, Kortenberg, Belgium; 5 Research Group Psychiatry, KU Leuven, Leuven, Belgium; 6 Department of Neurosciences, Research Group Psychiatry, Center for Clinical Psychiatry, KU Leuven, Leuven, Belgium; 7 Antwerp Health Law and Ethics Chair, AHLEC University Antwerpen, Antwerp, Belgium

**Keywords:** antidepressant, antiemetic, antiepileptic, dystonia, mood stabilizer

## Abstract

**Background:**

Medication-induced dystonia (MID) is a movement disorder (MD), characterized by involuntary sustained or intermittent muscle contractions, causing abnormal, often repetitive, movements, postures, or both. Although MID is commonly associated with the use of antipsychotics, it also occurs with many other medications widely used in clinical practice.

**Methods:**

A systematic literature search (from inception to November 2023), using the PubMed and Embase databases, was conducted without language restriction for articles reporting on MID in people without pre-existing MDs, and this for all potentially relevant non-antipsychotic medications. A narrative synthesis of the available evidence was undertaken.

**Results:**

MID is common (1 to 10%) with certain antiemetics. Selective serotonin reuptake inhibitors and the antiepileptics valproate, carbamazepine, and lamotrigine are rarely (0.01 to 0.1%) or very rarely (<0.01%) associated with MID. All other medications are very rarely (<0.01%) associated with MID or have a risk that cannot be precisely estimated. The actual rate of dystonic reactions with most non-antipsychotic agents remains unknown, owing to misdiagnosis and underreporting in the scientific literature. In general, MID seems to occur more often in children and adolescents, even with a single low dose, and with polymedication. In most cases, MID is acute in onset (occurring within hours to days) and involves the head and neck.

**Conclusions:**

Although MID is most common with dopamine receptor-blocking antiemetics, many other medications may also produce dystonic reactions, particularly in children and adolescents. Although such incidents remain rare, there are indications that MID is underreported for many classes of medications.

## Introduction

Dystonia is “a movement disorder (MD) characterized by involuntary sustained or intermittent muscle contractions, causing abnormal, often repetitive, movements, postures, or both” [1–7]. It is the third most common MD after essential tremor and Parkinson’s disease [2, 8, 9] and can affect any muscle group in the body (see Table 1) [1, 2, 4, 10]. Focal dystonias are the most common forms seen in clinical practice, involving the neck (cervical dystonia), the eyes (oculogyric crisis), the larynx (laryngeal dystonia), the mouth and jaw (oromandibular dystonia), or the limb (limb dystonia) [10–12]. Prevalence rates seem to be higher in female individuals for most types of dystonia [13].

**Table 1.** Classification of dystonia by body region [1, 2, 11].

Form	Definition
Focal	Involvement of one body region
Segmental	Involvement of two or more contiguous body regions
Multifocal	Involvement of two noncontiguous or more (contiguous or not) body regions
Generalized	The trunk and at least two other sites are involved

Dystonia may be inherited, idiopathic, or acquired [1, 2, 5]. Acquired dystonias result from apparent outside factors and can be attributed to a specific cause, such as medications [2, 5, 14]. Medications most commonly associated with this type of MD are antipsychotics [5, 10, 15, 16]. However, dystonia may also occur with many other kinds of medication, such as antidepressants, lithium, antiepileptics, and calcium channel blockers [1, 14, 15, 17–19]. Medication-induced dystonia (MID) can be acute (occurring within hours to days of exposure to the drug), subacute (building up more slowly after days to weeks of exposure), or tardive [following long-term therapy (months-years) with the offending drug] [10, 20–30]. MID mostly resolves within a few hours or days with adequate treatment. However, in some cases, it can be persistent [10].

Existing lacunae in understanding the epidemiology and phenomenology of MID face clinicians with substantial challenges in the diagnosis and management of this drug-induced MD [31–33]. MID may be confused with different conditions such as partial seizure, encephalitis, tetanus, hysteria, or panic attacks. In exceptional cases (i.e. acute laryngeal dystonia), misdiagnosis can lead to a life-threatening situation [16, 23, 34–37]. Early identification, therefore, is essential.

Until now, no extensive review on the epidemiology and phenomenology of MID across different non-antipsychotic medication groups has been conducted. Our objective therefore is to identify published evidence-based literature on the epidemiology and phenomenology of non-antipsychotic-induced dystonia in people without pre-existing MD by using a hybrid systematic-narrative strategy. This approach builds on the main components of both systematic and narrative reviews [38].

## Methods

The protocol of this systematic-narrative review has been registered with the Open Science Framework initiative (https://osf.io/uvpbn/).

### Search strategy

A comprehensive and systematic literature search (from inception to November 2023), using the PubMed and Embase databases, was conducted without language restriction for articles reporting on non-antipsychotic-induced dystonia in people without pre-existing MDs. One of the authors (JD) constructed search strings for both databases. Generic and brand drug names were used to identify cases of non-antipsychotic dystonia. Full search strategies are available as Supplementary Material. Articles, identified through PubMed and Embase, were imported into EndNote X9 and duplicates were removed [39]. After removing duplicates, titles and abstracts were screened by JD, using Rayyan QCRI. Articles that were deemed potentially relevant were selected. JD reviewed the full text of the selected articles and assessed their eligibility. Any doubts were solved by consensus or by the decision of a second and third reviewer (MDH, KC).

### Selection criteria

All types of study designs were eligible for inclusion. Although observational studies, case series, and case reports have lower levels of evidence, we found it important to implement this kind of evidence, as (randomized) clinical trials have limited power to detect rarer events, such as motor side effects [40, 41]. Only articles providing information on the epidemiology and phenomenology of non-antipsychotic-induced dystonia in people (children, adolescents, adults, and elderly) without a pre-existing MD were selected. A narrative synthesis of the systematically retrieved eligible articles was made.

## Results

The search yielded 58,326 articles. After removing duplicates (*n* = 39,662) a total of 718 systematic reviews and/or meta-analyses and 17,946 other records were screened for eligibility. Of these 40 systematic reviews and/or meta-analyses and 1,998 other records were identified as eligible.

For each non-antipsychotic medication group we will discuss, if this information is available, (1) epidemiology, (2) phenomenology [onset and form(s) of dystonia], (3) risk factors, and (4) agents that are specifically associated with an increased risk for dystonia. Among risk factors, race or ethnicity are not discussed as potential moderators. Although there are some studies that have indicated that for certain medications Asian patients may be more likely to experience MID, this has not been systematically studied.

## Antiemetics and gastrointestinal drugs

Antiemetics are widely used to treat nausea and vomiting that can be caused by a variety of medical conditions and situations, such as chemotherapy, surgery, migraine, and pregnancy [42–49].

*Metoclopramide* can induce the entire phenomenological spectrum of dystonia, even with a single low dose [16, 24, 50–66]. Metoclopramide-induced acute dystonia has been seen in 0.2% up to 8.3% of adult cases [24, 44, 55, 57, 59, 67–76]. The risk can even be higher in children and elderly [54, 70, 77], and is increased at higher doses or with long-term treatment [75]. It typically occurs within 24–48 h of initiating treatment [59].

Given the known risk of MID with metoclopramide, particularly with chronic use or in young people, the European Medicines Agency (EMA) and the Food and Drug Administration (FDA) restricted the indications for metoclopramide to short-term use (up to 5 days). In children, it should only be used as a second-choice treatment [78, 79]. Metoclopramide (primarily metabolized by the cytochrome P450 enzyme CYP2D6) dosing should also be reduced in CYP2D6 poor metabolizers. It therefore should not be co-administered with strong CYP2D6 inhibitors [50, 80–83].

Acute or subacute dystonic reactions with *prochlorperazine*, first introduced as an antipsychotic in the 1950s [84], are seen in up to 4% of cases [68, 77, 85].

Several studies and case reports reported *promethazine*-induced acute dystonia in children and in pregnant women hospitalized for hyperemesis gravidarum [86–94]. Promethazine seems to be associated with a higher risk for dystonia, compared to metoclopramide [92, 93], sometimes inducing severe acute dystonic reactions (e.g. opisthotonus) in overdose cases [90]. In 2000, a warning section was added to the medication package insert stating that promethazine is contraindicated in children less than 2 years of age [91].

Dystonia is a very rare complication when using *domperidone* (0.01%), as it does not traverse the blood–brain barrier, unlike metoclopramide. Domperidone-induced acute dystonia usually occurs in infants and very young children (due to the poorly developed blood–brain barrier) or in the elderly [45, 46, 95–98].

At recommended clinical dosages, dystonic reactions associated with *levosulpiride* occur in less than 1% [98–101]. Levosulpiride-induced MDs seem to occur more frequently in the elderly, requiring strict pharmacovigilance [102, 103]. In exceptional cases, even the use of low-dose levosulpiride can lead to persistent dystonia [104, 105].

Although uncommon, some setrons also have also been associated with acute dystonic reactions in adults, as well as children. *Ondansetron*, for example, can induce the entire phenomenological spectrum of dystonia [49, 65, 106–120].

*Clebopride*, a dopamine receptor blocking agent (DRBA) which is 10 times more potent than metoclopramide [120] but marketed only in some countries, is associated with the occurrence of different types of dystonic reactions (oromandibular dystonia, blepharospasm, torticollis) [48, 65, 98, 121–126], particularly in younger people, even after one single dose [121].

*Droperidol*-induced acute dystonia has, with the exception of few, been reported in several studies [127–133] and case reports [65, 134–138] and can be severe and persistent [136].

Other commonly used antiemetics or gastrointestinal drugs that have been rarely associated with dystonic reactions are *cimetidine*, *ranitidine*, *cyclizine*, and *cisapride* [50, 106, 108, 139–148].

## Antiepileptics

Antiepileptics (also known as antiseizure medications or anticonvulsants) are commonly prescribed for epilepsy/seizures prophylaxis or management, as well as for many other indications, such as bipolar disorder, anxiety, migraine, chronic pain, weight management, and insomnia [149].

The relationship between antiepileptics and MDs is complex. Although antiepileptics are used as a treatment for hyperkinetic MDs (specifically for tremor, myoclonus, and restless legs syndrome), several also have the potential to induce or worsen MDs, including dystonia [150, 151]. Four of these have been rarely (0.01 to 0.1%) associated with dystonia: valproate, carbamazepine, lamotrigine, and phenytoin. There have been more reports of MID with these agents in the middle-aged adult population.

*Valproate* is generally regarded as a first-choice agent for most forms of epilepsy, but it is also used to treat manic episodes, and as a medication for migraine prevention and impulse control [152]. Although tremor and parkinsonism are well-known side effects of valproate [150, 151, 153–155], dystonic reactions, most often subacute (> 3 weeks) and presenting as axial and cervical dystonia, have also been reported [151, 154]. Possible interactions with clozapine, risperidone, quetiapine, olanzapine, carbamazepine, ziprasidone, and butamirate citrate have been described [154].

Particularly children and adolescents seem to be susceptible to the development of *carbamazepine*-induced dystonia [156]. A recent systematic review identified 22 cases of carbamazepine-induced, mostly subacute (> 3 weeks), dystonia [151]. Generalized or segmental dystonia and oculogyric crises have been reported within normal and toxic plasma concentrations of carbamazepine. The combination of carbamazepine and isoniazid or lithium has been reported to induce oculogyric crisis and severe dystonic movements, including opisthotonos [150].

*Lamotrigine*, also used as a mood stabilizer for the treatment of bipolar disorder, most often is associated with the subacute (> 3 weeks) manifestation of blepharospasms, oculogyric crises, and oromandibular dystonia [150, 151, 157].

Mostly subacute (> 3 weeks) dystonic reactions have been reported with *phenytoin* at therapeutic and toxic serum levels [15, 151, 158–161]. The most common presentation seems to be upper limb dystonia.

Dystonia, although very rarely (<0.01%), has also been reported in association with *other antiepileptics* (see Table 2 for an overview of these antiepileptics), sometimes related to polymedication [162–164].

**Table 2.** Higher risk medications that require special attention from healthcare professionals.

Medication class	Medication	Frequency^1^
Antiemetics and gastrointestinal drugs	Clebopride	Common
	Domperidone	Rare
	Metoclopramide	Common
	Prochlorperazine	Common
	Promethazine	Uncommon
Calcium antagonists (medication for migraine and dizziness)	Cinnarizine	Rare
	Flunarizine	Rare
Antiepileptics	Carbamazepine	Rare
	Phenytoin	Rare
	Lamotrigine	Rare
	Valproate and valproic acid	Rare
Antidepressants	Fluoxetine	Rare
	Fluvoxamine	Rare
	Paroxetine	Rare
Mood stabilizers	Lithium	Rare
ADHD medication	Methylphenidate (combined with an antipsychotic)	Rare

1 Very common (≥10%); common (≥1% to <10%); uncommon (≥0,1% to <1%); rare (≥0,01% to <0,1%).

## Antidepressants

Selective serotonin reuptake inhibitors (SSRIs) and serotonin and norepinephrine reuptake inhibitors (SNRIs) are the most commonly prescribed types of antidepressant medication [165, 166]. These medications have a number of approved indications (such as major depression, obsessive compulsive disorder, and anxiety disorders) [167, 168], but are frequently used off-label as well. Tricyclic antidepressants (TCAs) and monoamine oxidase inhibitors (MAOIs) are prescribed less often because they tend to cause more side effects.

Although uncommon, cases of antidepressant-induced acute and tardive dystonia have been observed and reported for decades [65, 139, 166, 168–180]. These side effects are seen more often with SSRIs than with SNRI’s, TCAs, MAOIs, or other antidepressants [169, 173, 181, 182].

## Selective serotonin reuptake inhibitors (SSRIs)

According to a review of Hawthorne & Caley [166], *citalopram, escitalopram, fluoxetine*, and *sertraline* are most frequently involved in dystonia cases. In 63% of the cases, dystonia occurred mostly subacute within 7 days of treatment initiation or dose increase (although acute or tardive dystonia cases have also been observed). Most cases of dystonia occurred in adult patients who have been receiving normal dosing and when a DRBA (mostly an antipsychotic) was added to the SSRI. Cases across the whole spectrum of dystonic reactions were observed. After the publication of this review several new cases of MID have been reported with sertraline and escitalopram, mostly in the adult population [167, 168, 174, 175, 183–186], but some of these also in the pediatric and adolescent population [167, 187–189].

An analysis of the WHO pharmacovigilance database found that the SSRIs fluoxetine, *fluvoxamine*, and *paroxetine* were statistically significantly associated with dystonia [173].

### *Serotonin and* norepinephrine *reuptake inhibitors (SNRIs)*

Without providing specific information on dystonia cases, an analysis of FDA Adverse Event Reporting System cases [177], as well as a large epidemiological study [190] identified the SNRI *duloxetine* as the antidepressant showing the highest association with EPS, compared with other antidepressants. A more recent analysis of MID reports in the WHO Pharmacovigilance database [173], however, showed no statistically significant association between duloxetine and dystonia.

### *Serotonin receptor* antagonist *and reuptake inhibitors (SARIs)*

*Trazodone*, the prototype drug of this class of drugs, is approved for the treatment of major depressive disorder but is also commonly used off-label to treat insomnia or delirium, particularly in the elderly [170, 191]. Although only few cases have been reported in the scientific literature [170, 191–194], clinicians should be aware that long-term use of trazodone as a hypnotic, particularly when combined with an antipsychotic, such as risperidone, can cause tardive dystonia in elderly patients [191].

### *Serotonin and* norepinephrine *disinhibitors (SNDIs)*

A literature review on *mirtazapine*, primarily used for the treatment of major depressive disorder, but also for several other off-label indications such as insomnia, migraine, and hot flushes, identified only five cases of dystonia (particularly in the elderly) [195].

### *Tricyclic antidepressants (*TCAs*)*

Although less common than with SSRIs, dystonia cases have been reported with the TCAs *amitriptyline, amoxapine, doxepin, imipramine*, and *clomipramine* [65, 173, 182, 196–198]. According to a review of 48 reports, examining the link between amitriptyline and MDs, patients with amitriptyline-induced dystonia (*n* = 19) tended to be younger and were prescribed a lower dose of amitriptyline [197]. A postmarketing study in the world pharmacovigilance database [173] found that amoxapine is the TCA associated with the highest risk for dystonia. It may induce several forms of subacute and tardive dystonia, including cervical dystonia and oculogyric crisis [199–201].

### *Monoamine oxidase* inhibitors *(MAOIs)*

EPS (including dystonia) have infrequently been reported during treatment with MAOIs [173, 182]. According to a postmarketing study in the world pharmacovigilance database [173], none of the studied MAOIs (isocarboxazid, phenelzine, tranylcypromine, moclobemide) was significantly associated with dystonia. Despite this, acute and subacute forms of dystonia have been reported with *tranylcypromine* (truncal dystonia) and *phenelzine* (oculogyric crisis and cervical dystonia), respectively [202, 203].

### Combination drugs

Although most GPs are aware that antipsychotics can induce EPS, they may be less aware that patients treated with a combination drug^1^, including an antipsychotic, may also be at risk to develop dystonia. One such example is a combination of the first-generation antipsychotic flupentixol (0.5 mg) and the tricyclic antidepressant melitracen (10 mg). Many GPs and neurologists prescribe this medication for depression, anxiety, or neurotic symptoms [204–206], for example in patients with irritable bowel syndrome [205]. Although no cases of dystonia in the scientific literature have been identified with this combination drug, there are indications that dystonia can be induced with long-term daily use of this medication (personal communication). Moreover, this combination drug is not approved for use and marketing in several developed countries, including the United States and the United Kingdom [204]. In India, it was even banned [207]. Although still registered in Belgium, the Belgian Centre for Pharmacotherapeutic Information strongly advises against using this combination drug to treat patients with depression.

### Lithium

A recent review [208] found dystonia to be the fourth most common MD with *lithium* (after parkinsonism, dyskinesia, and myoclonus). Twenty-two of the 436 identified MD cases concerned individuals who developed all forms of dystonia (including blepharospasm, oromandibular, cervical, distal segmental, axial, and lingual dystonia). Interestingly, one of every two individuals developing lithium-induced dystonia was from Asia. These patients were also significantly younger than the subjects presenting other MDs. The onset of dystonia varied between 1 day and 25 years. In about one-fourth of the identified cases, an antipsychotic was used. However, it is important to recognize dystonia as a potential complication of lithium, not only when administered in combination with an antipsychotic, but even when it is used as monotherapy or combined with small doses of other non-DRBA, especially during long-term use [209–211].

### Stimulants

*Methylphenidate* (MPH) is often used as a treatment for children and adolescents with ADHD with or without comorbid conduct-dissocial disorder [212].^.^Most reported MPH-induced dystonia cases in children and adolescents have occurred after initiation or up-titration of MPH. These cases involved MPH monotherapy [213] and combined MPH-second generation antipsychotic treatment [41, 214, 215]. A review of case reports and an analysis of the WHO pharmacovigilance database on the occurrence of MDs in children and adolescents using a combination therapy of MPH and the antipsychotic risperidone identified 4 case reports and 32 individual case safety reports (ICSRs) describing dystonic movements in relation to the combination therapy. Among the ICSRs, dystonia was the second most reported MD, and cases across the whole spectrum of dystonic reactions were observed [41]. Dystonia with MPH has also been reported in combination with other antipsychotics and medications known to have a risk of inducing dystonia (aripiprazole, propofol) [216], after prolonged use [212], or in the context of MPH withdrawal during psychostimulant detoxification [217].

## Antihistamines

MID due to the use of antihistamines has been very rarely reported [47, 218–222].

*Cetirizine* is a frequently used antihistamine for the treatment of allergic disorders in children. Several cases of cetirizine-induced acute (even after a single oral dose at recommended dosages), subacute, or tardive dystonia, such as oculogyric crisis, cervical, and oromandibular dystonia, in (mostly) children and adults, have been reported in the literature [47, 65, 218, 221–223].

Despite its widespread use in the management of MID [224], the first-generation antihistaminergic *diphenhydramine*, paradoxically, has also been recognized as a contributor to acute dystonia in very rare cases. The onset of dystonic reactions is usually rapid, developing shortly after taking the antihistamine. However, such reactions may also occur after long-term therapy. Patients characteristically develop facial dystonia, torticollis, and extremities dystonia [225–231].

Although very uncommon (but probably more common than reported) [225], MID with cough and cold preparations having antihistaminic properties (such as the widely used *cloperastine*-based cough syrup), has also been described. Oculogyric crisis and torticollis are among the most frequent dystonic reactions, with children being more susceptible than adults [218, 225, 232].

Finally, few cases of dystonia following *hydroxyzine* administration (widely used for skin allergies) have been reported [233, 234].

It is likely that the risk of MID increases when antipsychotics and (preparations containing) antihistamines are administered concomitantly, particularly in vulnerable individuals (e.g. chronic pretreatment with anti-dopaminergic drugs) [225, 235].

## Calcium channel blockers

Calcium channel blockers (CCBs) are medicines that are most often used to treat conditions of the heart and blood vessels, such as hypertension, angina, and cardiac arrhythmias. Besides these indications, they are also frequently prescribed for the treatment of migraine, vertigo, and cerebrovascular insufficiency [236].

Most CCB-induced MDs are reported with *flunarizine* and *cinnarizine.* According to an analysis of patients who have been taking flunarizine (*n* = 26,133) or cinnarizine (*n* = 7,186) for more than 1 month, both agents significantly increased the risk of subacute or tardive dystonia [incidence rates of flunarizine- and cinnarizine-induced dyskinesia/dystonia were 1.21(0.81–1.78) and 1.52(0.79–2.92) per 10,000 person months, respectively]. However, as many of the patients in this study used antipsychotics or metoclopramide concomitantly, the risk of flunarizine- or cinnarizine-related MDs might have been overestimated [237]. In the study of Fabiani et al. [238] dystonia was diagnosed in 4% of the patients due to the chronic use of cinnarizine and flunarizine. Flunarizine-related MDs (including dystonia) are associated with a high-dose exposure, longer exposure duration, older age, history of essential tremor, and cardiovascular diseases [236].

Some case reports described acute and tardive (persistent) dystonic reactions induced by the CCBs *verapamil* [239–241], *nifedine* [242, 243], and *amlodipine* (inducing cranial, cervical, pharyngo-laryngeal, or axial dystonia) [18], and the antiarrhythmic drug *flecainide* [244].

## Antimalarials

Acute dystonia (oromandibular dystonia and oculogyric crisis) induced by *chloroquine*, commonly used for both the prevention and treatment of malaria, is very rare [139, 245, 246]. It mainly has been reported after a single dose of chloroquine, in the presence [247] (particularly in combination with the common antibiotic metronidazole) [248] or absence of other medications [246].

The are some case-reports of *artesunate/amodiaquine and artemether/lumefantrine*-induced acute dystonia (oculogyric crisis) in the literature [248, 249]. Artemether/lumefantrine treatment may cause dystonic reactions in patients at any age, even at therapeutic dosages [250].

### Other medications

Dystonic reactions, although rarely observed, have been reported with several *antibiotics* [65, 243, 251–265] and *antiviral drugs* [266, 267] (see Table 3), which usually are acute and may involve the whole spectrum of dystonia. Many other medications have been found to induce dystonia (particularly when used in combination with other agents), in most cases involving the head and neck: several *opioid analgesics* (e.g. fentanyl) [21, 139, 243, 268–271], the *non-opioid anesthetic* propofol [21, 113, 139, 233, 268, 272–280] (sometimes inducing full opisthotonus or laryngeal dystonia), the *inhalational anesthetic* sevoflurane (particularly associated with an increased risk of laryngospasm, potentially leading to laryngeal dystonia, especially in children) [21, 269, 281–288], the *analgesic and antipyretic drug* paracetamol (although acute dystonia with therapeutic doses of paracetamol is very unusual) [288], several *antitussives* [225, 232, 235, 289–293] (often associated with cervical dystonia), the *anthelminthic drug* albendazole (particularly in sensitive children) [294, 295], the *histamine analog* betahistine (largely used in the treatment of Ménière’s disease and also having the propensity to induce tardive dystonia after prolonged use) [296–298], the *cytostatic drug* capecitabine [299–301] (typically associated with oromandibular dystonia), *tetrabenazine* (a medication mainly used in patients with hyperkinetic MDs, including dystonia, that may, however, worsen dystonia particularly in vulnerable young adults) [65, 139, 302–305], *isotretinoin* (a medication used to treat severe acne that can induce oculogyric crisis) [306], and the *immunosuppressant agents* cyclosporine (rarely causing limb or focal hand dystonia that may persist after cyclosporine withdrawal) [307, 308] and tacrolimus (strongly associated with dystonia, particularly in female pediatric patients) [309]. Concerning analgesic-induced dystonia particularly female patients seem to be vulnerable, as women might respond differently to general anesthetic agents, compared to men [310]. *Cholinesterase inhibitors*, widely used in patients with Alzheimer’s disease and in patients with myasthenia gravis, seem to be particularly associated with the Pisa Syndrome, also known as pleurothotonus, a term used to describe a type of acute or tardive truncal dystonia [65, 311–318]. Finally, several *benzodiazepines* have been associated with acute and tardive dystonia (including opisthotonus) in adults and children [319–322] (See Table 3). For example, long-term use of etizolam, zolpidem, and brotizolam may result in blepharospasms, especially in women [323, 324].

**Table 3.** Medications very rarely (<0,01%) associated with dystonia or for which this risk cannot be precisely estimated.

Medication class	Medication
Antibiotics	Cefalexine
	Cefepime
	Cefixime
	Cefuroxime
	Ciprofloxacin
	Erythromycin
	Gemifloxacin
	Levofloxacin
	Metronidazole
	Spiramycin
Antiemetics and gastrointestinal drugs	Cimetidine
	Cisapride
	Itopride
	Ondansetron
	Ranitidine
	Tropisetron
Antidepressants	Amitriptyline
	Amoxapine
	Bupropion
	Citalopram
	Clomipramine
	Doxepin
	Duloxetine
	Escitalopram
	Flupentixol-Melitracen (!!!)
	Imipramine
	Mirtazapine
	Sertraline
	Venlafaxine
	Trazodone
Antiepileptics	Clobazam
	Felbamate
	Gabapentin
	Midazolam
	Oxcarbazepine
	Perampanel
	Phenobarbital
	Pregabalin
	Tiagabine
	Topiramate
	Vigabatrin
Cholinesterase inhibitors	Donepezil
	Galantamine
	Rivastigmine
Opioid analgesics	Fentanyl
	Meperidine
	Morphine
	Pentazocine
Analgesics and antipyretics	Paracetamol
Antitussives	Butamirate citrate
	Cloperastine
	Codeine
	Dextromethorphan (although almost none of the cases of dextromethorphan-induced dystonia has been reported within the therapeutic range)
Calcium antagonists	Nifedipine
	Amlodipine
	Verapamil
	Flecainide
Anesthetics	Nitric oxide
	Propofol (particularly with certain propofol combination regimens that include either ketamine or dexmedetomidine)
	Sevoflurane (particularly when administered in combination with an antipsychotic)
Antiallergics	Cetirizine
	Diphenhydramine
	Hydroxyzine
Antivirals	Foscarnet
	Lamivudine
Antimalarials	Artesunate/amodiaquine
	Hydroxychloroquine
	Artemether/lumefantrine
Sleep medication, sedatives, and anxiolytics (mostly long-term use)	Bromazepam (BDZ)
	Brotizolam (BDZ)
	Clobazam (BDZ)
	Midazolam (BDZ)
	Diazepam (BDZ)
	Zolpidem
	Etizolam
Retinoids	Isotretinoin
Cytostatics	Capecitabine
Anthelminthics	Albendazole
Histamine analogs	Betahistine
Vesicular monoamine transporter 2 (VMAT2) inhibitors	Tetrabenazine
Immunosuppressive agents	Cyclosporine
	Tacrolimus

BDZ: Benzodiazepine

(!!!): Belgian Centre for Pharmacotherapeutic Information strongly advises against the use of this medication

## Discussion

The rates of MID probably are underestimated [102, 325, 326]. The Hannover epidemiology study [11], which considered all forms of dystonia (including DRBA-induced dystonia) in highly specialized centers, estimated dystonia rates to be at least four times higher than previously thought. There are indications that dystonia is also underreported for several other classes of medications, including antidepressants, antiemetics, and cholinesterase inhibitors [63, 75, 173, 175, 180, 318, 325]. Revet et al. [173], for example, identified 5,113 dystonia cases (0.50%) (on a total of 1,027,405 reported cases containing at least one of the 58 selected antidepressant drugs) in the WHO pharmacovigilance database during the time period of January 1967 to February 2017. This means that the prevalence of dystonia for antidepressants, as a group, lies between ≥0.1% to <1% (= uncommon side effect), while the frequency of this side effect for each antidepressant has been rated by the authors of this article as rare or very rare (see Tables 2 and 3).

There are several reasons why MID might be underreported. Firstly, only few individual studies or systematic reviews/meta-analyses on medication-induced EPS mention dystonia as a separate category because of the smaller numbers of this MD, compared to these for other MD, such as dyskinesia, akathisia, or parkinsonism. Secondly, although it is generally well-known to GPs that dystonia is commonly associated with the use of DRBAs such as high-potency antipsychotics, they do not expect it to be an adverse drug reaction (ADR) associated with medications widely used in general clinical practice, such as antidepressants, antibiotics, antivirals, antiallergics, and antitussives. Moreover, many GPs are not familiar with the clinical presentation of acute dystonia. This leads to a higher likelihood of misdiagnosis [325]. Finally, the severity spectrum of dystonia can be extremely large. Dystonia might be a subtle finding, rather than a complaint, without a serious consequence for the patient [11]. Under these circumstances, GPs may interpret this ADR as not important. However, in exceptional cases (i.e. laryngeal dystonia) MID can be life-threatening [20, 31, 37, 277, 327–336]. The patient can develop acute respiratory distress through upper airway obstruction showing signs, such as cyanosis, stridor, gasping, and an inability to manage secretions [34, 211, 337, 338]. Acute laryngeal dystonia can easily be misdiagnosed as anaphylaxis, epiglottitis, hysteria, panic attack, or acute anxiety [23, 34–36]. Prompt recognition therefore can save lives. The sudden onset of symptoms with rapid progression in the presence of a dystonia risk profile should caution the health professional [339]. Characteristic symptoms of laryngeal dystonia are dyspnea, laryngeal stridor, and extreme distress. Laryngeal dystonia may also be accompanied by dystonia in other parts of the body [31, 37].

The treatment of dystonia typically involves discontinuing the offending drug (due to the risk of a recurrent dystonic reaction) and administration of medications that block the acetylcholine receptors (i.e. anticholinergics, benzodiazepines, and certain antihistamines) [11, 20, 24, 327]. However, symptoms may reoccur within hours after initial treatment. In these cases, clinicians should give another dose of the medication or administer the medication for several days to prevent the reoccurrence of dystonia [327, 340].

## Supporting information

Catthoor et al. supplementary material Catthoor et al. supplementary material

## Footnotes

1 Medications that include two or more active ingredients combined in a single form at a fixed dose, of which at least one is associated with an increased risk of dystonic reactions.

## Supplementary material

The supplementary material for this article can be found at http://doi.org/10.1192/j.eurpsy.2025.18.
