# Supplementary material for: The epidemiology and phenomenology of non-antipsychotic-induced dystonia: a hybrid systematic-narrative review
Source: Eur Psychiatry. 2025 Feb 10;68(1):e36. doi: 10.1192/j.eurpsy.2025.18 (PMC11883798; doi:10.1192/j.eurpsy.2025.18)
Supplement: Catthoor et al. supplementary material [file S0924933825000185sup001.docx]

**APPENDIX Search Strategies**

**PUBMED**

Dystonia

("Dyskinesias"[Mesh:NoExp] OR "Dystonia"[Mesh] OR "Movement Disorders"[Mesh:NoExp] OR "Dystonic Disorders"[Mesh] OR "Blepharospasm"[Mesh] OR "Muscle Cramp"[Mesh] OR "dyston*"[tiab] OR "myodystonia"[tiab] OR "myodystony"[tiab] OR "torticollis"[tiab] OR "retrocollis"[tiab] OR "anterocollis"[tiab] OR "laterocollis"[tiab] OR "oculogyric*"[tiab] OR "opisthotonus"[tiab] OR "opisthotonos"[tiab] OR "laryngospasm"[tiab] OR "wryneck"[tiab] OR "wry neck"[tiab] OR "loxia"[tiab] OR "muscle tonus"[tiab] OR "motor dysfunction*"[tiab] OR "torsion spasm*"[tiab] OR "Meige*"[tiab] OR "Brueghel*"[tiab] OR "blepharospasm*"[tiab] OR "spasmodic dysphonia*"[tiab])

Antiemetics and gastrointestinal drugs

("Antiemetics"[Mesh] OR "antiemetic*"[tiab] OR "anti-emetic*"[tiab] OR "Serotonin 5-HT3 Receptor Antagonists"[Mesh] OR "5-HT3 receptor antagonist*"[tiab] OR "5-HT3 receptor block*"[tiab] OR "5-HT3 antagonist*"[tiab] OR "5-HT 3 antagonist*"[tiab] OR "5-HT3 block*"[tiab] OR "serotonin 3 antagonist*"[tiab] OR "Dopamine D2 Receptor Antagonists"[Mesh] OR "dopamine D2 receptor antagonist*"[tiab] OR "dopamine 2 receptor antagonist*"[tiab] OR "dopamine D2 receptor block*"[tiab] OR "dopamine 2 receptor block*"[tiab] OR "dopamine D2 antagonist*"[tiab] OR "dopamine 2 antagonist*"[tiab] OR "dopamine D2 block*"[tiab] OR "dopamine 2 receptor blocking agent*"[tiab] OR "Neurokinin-1 Receptor Antagonists"[Mesh] OR "neurokinin-1 receptor antagonist*"[tiab] OR "neurokinin-1 antagonist*"[tiab] OR "NK1 antagonist*"[tiab] OR "neurokinin-1 receptor block*"[tiab] OR "tachykinin receptor 1 antagonist*"[tiab] OR "tachykinin receptor 1 blocker*"[tiab] OR "substance P receptor antagonist*"[tiab] OR "substance P receptor blocker*"[tiab] OR "Adrenal Cortex Hormones"[Mesh] OR "adrenal cortex hormone*"[tiab] OR "adrenal cortical hormone*"[tiab] OR "adrenocortical hormone*"[tiab] OR "adrenocortical steroid*"[tiab] OR "adrenocorticosteroid*':ti,ab,kw OR 'cortical steroid*"[tiab] OR "adrenal cortical steroid*"[tiab] OR "adreno cortical steroid*"[tiab] OR "adreno corticosteroid*"[tiab] OR "adrenal steroid*"[tiab] OR "corticosteroid*"[tiab] OR "cortico steroid*"[tiab] OR "corticoid*"[tiab] OR "Histamine H1 Antagonists"[Mesh] OR "H1 receptor antagonist*"[tiab] OR "Histamine 1 receptor antagonist*"[tiab] OR "Histamine 1 receptor block*"[tiab] OR "H1 antihistamine*"[tiab] OR "H1 receptor block*"[tiab] OR "H1 antagonist*"[tiab] OR "H1 block*"[tiab] OR "H1 receptor blockader*"[tiab] OR "Cholinergic Antagonists"[Mesh] OR "cholinergic receptor antagonist*"[tiab] OR "cholinergic receptor block*"[tiab] OR "cholinergic antagonist*"[tiab] OR "cholinergic block*"[tiab] OR "cholinergic blocking agent*"[tiab] OR "cholinolytic*"[tiab] OR "acetylcholine antagonist*"[tiab] OR "acetylcholine receptor block*"[tiab] OR "acetylcholine receptor inhibitor*"[tiab] OR "AChR inhibitor*"[tiab] OR "acetylcholine block*"[tiab] OR "acetylcholine blocking agent*"[tiab] OR "anticholinergic*"[tiab] OR "anti-cholinergic*"[tiab] OR "parasympathetic agent*"[tiab] OR "parasympathetic drug*"[tiab] OR "parasympathetic block*"[tiab] OR "parasympatholytic agent*"[tiab] OR "parasympatholytic drug*"[tiab] OR "parasympatholytic block*"[tiab] OR "parasympatholytic*"[tiab] OR "parasympathicolytic agent*"[tiab] OR "parasympathicolytic drug*"[tiab] OR "parasympathicolytic block*"[tiab] OR "cholinolytic agent*"[tiab] OR "atropinic agent*"[tiab] OR "atropinic drug*"[tiab] OR "Cholinergic Antagonists"[Mesh] OR "cholinergic receptor antagonist*"[tiab] OR "cholinergic receptor block*"[tiab] OR "cholinergic antagonist*"[tiab] OR "cholinergic block*"[tiab] OR "cholinergic blocking agent*"[tiab] OR "cholinolytic*"[tiab] OR "acetylcholine antagonist*"[tiab] OR "acetylcholine receptor block*"[tiab] OR "acetylcholine receptor inhibitor*"[tiab] OR "AChR inhibitor*"[tiab] OR "acetylcholine block*"[tiab] OR "acetylcholine blocking agent*"[tiab] OR "anticholinergic*"[tiab] OR "anti-cholinergic*"[tiab] OR "parasympathetic agent*"[tiab] OR "parasympathetic drug*"[tiab] OR "parasympathetic block*"[tiab] OR " parasympatholytic agent*"[tiab] OR "parasympatholytic drug*"[tiab] OR "parasympatholytic block*"[tiab] OR "parasympatholytic*"[tiab]OR "parasympathicolytic agent*"[tiab] OR "parasympathicolytic drug*"[tiab] OR "parasympathicolytic block*"[tiab] OR "cholinolytic agent*"[tiab] OR "atropinic agent*"[tiab] OR "atropinic drug*"[tiab] OR "Ondansetron"[Mesh] OR "ondansetron*"[tiab] OR "ad 04"[tiab] OR "ad04"[tiab] OR "avessaron"[tiab] OR "bryterol"[tiab] OR "cedantron"[tiab] OR "cellondan*"[tiab] OR "ceramos"[tiab] OR "emeset"[tiab] OR "gr 38032"[tiab] OR "gr 38032f"[tiab] OR "gr c507 75"[tiab] OR "gr38032"[tiab] OR "gr38032f"[tiab] OR "modifical"[tiab] OR "narfoz"[tiab] OR "onsia"[tiab] OR "sakisozin"[tiab] OR "setofilm"[tiab] OR "sn 307"[tiab] OR "sud 002"[tiab] OR "sud002"[tiab] OR "suda 002"[tiab] OR "suda002"[tiab] OR "vomceran"[tiab] OR "zensana"[tiab] OR "zetron"[tiab] OR "zofrene"[tiab] OR "zofron"[tiab] OR "zondamist"[tiab]OR "zofran*"[tiab] OR "zophran"[tiab] OR "zophren"[tiab] OR "zuplenz"[tiab] OR "Granisetron"[Mesh] OR "granisetron*"[tiab] OR "apf 530"[tiab] OR "apf530"[tiab] OR "axigran"[tiab] OR "az 010"[tiab] OR "az010"[tiab] OR "brl 43694"[tiab] OR "brl 43694a"[tiab] OR "brl43694"[tiab] OR "brl43694a"[tiab] OR "cam 2047"[tiab] OR "cam2047"[tiab] OR "emegar"[tiab] OR "eutrom"[tiab] OR "granegis"[tiab] OR "grani-denk"[tiab] OR "granicip"[tiab] OR "granigen"[tiab] OR "granisol"[tiab] OR "granissetrom"[tiab] OR "granitron"[tiab] OR "granizetron"[tiab] OR "kevatril"[tiab] OR "kytril"[tiab] OR "pandiol"[tiab] OR "rasetron"[tiab] OR "sancuso"[tiab] OR "sp 01"[tiab] OR "sp01"[tiab] OR "sustol"[tiab] OR "taraz"[tiab] OR "viatrinil"[tiab] OR "dolasetron" [Supplementary Concept] OR "dolasetron*"[tiab] OR "Anemet"[tiab] OR "Anzemet"[tiab] OR "mdl 73147"[tiab] OR "mdl 73147ef"[tiab] OR "mdl73147"[tiab] OR "mdl73147ef"[tiab] OR "zamanon"[tiab] OR "Palonosetron"[Mesh] OR "palonosetron*"[tiab] OR "Aloxi"[tiab] OR "nuowei"[tiab] OR "onicit"[tiab] OR "paloxi"[tiab] OR "rs 25259"[tiab] OR "rs 25259 197"[tiab] OR "rs 25259-197"[tiab] OR "rs25259"[tiab] OR "rs25259 197"[tiab] OR "rs25259-197"[tiab] OR "ruo shan"[tiab] OR "Tropisetron"[Mesh] OR "tropisetron*"[tiab] OR "endoprol"[tiab] OR "endostem"[tiab] OR "ics 205 930"[tiab] OR "ics 205, 930"[tiab] OR "ics 205-930"[tiab] OR "ics 205930"[tiab] OR "ics-205930"[tiab] OR "Navoban"[tiab] OR "novaban"[tiab] OR "novoban"[tiab] OR "Metoclopramide"[Mesh] OR "metoclopramid*"[tiab] OR "ahr 3070 c"[tiab] OR "ahr 3070c"[tiab] OR "ahr3070c"[tiab] OR "ametic"[tiab] OR "anausin"[tiab] OR "apo-metoclop"[tiab] OR "aputern"[tiab] OR "betaclopramide"[tiab] OR "bondigest"[tiab] OR "carnotprim primperan"[tiab] OR "cerucal"[tiab] OR "clodilion"[tiab] OR "clopamon"[tiab] OR "clopan"[tiab] OR "clopra"[tiab] OR "clopra-yellow"[tiab] OR "clopram"[tiab] OR "degan"[tiab] OR "del 1267"[tiab] OR "del1267"[tiab] OR "dibertil"[tiab] OR "duraclamid"[tiab] OR "emenil"[tiab] OR "emetal"[tiab] OR "emetard"[tiab] OR "emitasol"[tiab] OR "emperal"[tiab] OR "encil"[tiab] OR "enzimar"[tiab] OR "gastro timelets"[tiab] OR "gastrobi*"[tiab] OR "gastronerton"[tiab] OR "gastrosil"[tiab] OR "gastrotem"[tiab] OR "gastrotimelets"[tiab] OR "gavistal"[tiab] OR "gensil"[tiab] OR "gimoli"[tiab] OR "gimoti"[tiab] OR "hemesis"[tiab] OR "hyrin"[tiab] OR "imperan"[tiab] OR "m 813"[tiab] OR "m813"[tiab] OR "maalox nausea"[tiab] OR "maril"[tiab] OR "maxeran"[tiab] OR "maxeron"[tiab] OR "maxolan"[tiab] OR "maxolon"[tiab] OR "mcp-beta tropfen"[tiab] OR "meclomid"[tiab] OR "meclopamide"[tiab] OR "meclopramide"[tiab] OR "meclopran"[tiab] OR "megaldrate"[tiab] OR "meramide"[tiab] OR "metaclopramide"[tiab] OR "metadrate"[tiab] OR "metagliz"[tiab] OR "metamide"[tiab] OR "methochlopramide"[tiab] OR "methoclopramide"[tiab] OR "methoclopramine"[tiab] OR "metlazel"[tiab] OR "metochlopramide"[tiab] OR "metoclopamide"[tiab] OR "metoclopramine"[tiab] OR "metoclopranide hydrochloride"[tiab] OR "metoclor"[tiab] OR "metoclorpramide"[tiab] OR "metocobil"[tiab] OR "metocyl"[tiab] OR "metodopramide"[tiab] OR "metolon"[tiab] OR "metopram"[tiab] OR "metox"[tiab] OR "metozolv*"[tiab] OR "metpamid"[tiab] OR "metram"[tiab] OR "moriperan"[tiab] OR "mygdalon"[tiab] OR "nausil"[tiab] OR "neopramiel"[tiab] OR "netaf"[tiab] OR "neu sensamide"[tiab] OR "nic 001"[tiab] OR "nic001"[tiab] OR "nilatika"[tiab] OR "normastin"[tiab] OR "octamide"[tiab] OR "opram"[tiab] OR "paspertin*"[tiab] OR "perinorm"[tiab] OR "pharmyork"[tiab] OR "plasil"[tiab] OR "pramidin"[tiab] OR "pramin"[tiab] OR "pramotel"[tiab] OR "primperan"[tiab] OR "primperil"[tiab] OR "prinparl"[tiab] OR "prokinyl lp"[tiab] OR "prowel"[tiab] OR "pulin"[tiab] OR "reclomide"[tiab] OR "reglan*"[tiab] OR "reliveran"[tiab] OR "rimetin"[tiab] OR "sensamide"[tiab] OR "sotatic-10"[tiab] OR "terperan"[tiab] OR "tomid"[tiab] OR "vertivom"[tiab] OR "vomitrol"[tiab] OR "zumatrol"[tiab] OR "Prochlorperazine"[Mesh] OR "prochlorperazine*"[tiab] OR "Compazine"[tiab] OR "6140 rp"[tiab] OR "antinaus"[tiab] OR "az 001"[tiab] OR "az001"[tiab] OR "bayer a 173"[tiab] OR "bayer a173"[tiab] OR "capazine"[tiab] OR "chlormeprazine"[tiab] OR "chlorpeazine"[tiab] OR "chlorperazine"[tiab] OR "compro"[tiab] OR "dicopal"[tiab] OR "emelent"[tiab] OR "klometil"[tiab] OR "kronocin"[tiab] OR "meterazine"[tiab] OR "metherazine"[tiab] OR "nautisol"[tiab] OR "nipodal"[tiab] OR "normalmin"[tiab] OR "pasotomin"[tiab] OR "prochlor"[tiab] OR "prochlor perazine"[tiab] OR "prochlorpemazine"[tiab] OR "prochlorperacine"[tiab] OR "prochlorperzine"[tiab] OR "prochlorpromazine"[tiab] OR "proclorperazine"[tiab] OR "rp 6140"[tiab] OR "rp6140"[tiab] OR "sk and f 4657"[tiab] OR "skf 4657"[tiab] OR "skf4657"[tiab] OR "tementil"[tiab] OR "temetil"[tiab] OR "Droperidol"[Mesh] OR "droperidol"[tiab] OR "dehidrobenzoperidol"[tiab] OR "dehydrobenzoperidol"[tiab] OR "dehydrobenzoperiol"[tiab] OR "dehydrobenzperidol"[tiab] OR "dehydrobenzperidolum"[tiab] OR "dridol"[tiab] OR "droleptan"[tiab] OR "droperol"[tiab] OR "halkan"[tiab] OR "inaprine"[tiab] OR "inapsin*"[tiab] OR "mcn jr 4749"[tiab] OR "mcn r 4749"[tiab] OR "oridol"[tiab] OR "r 4749"[tiab] OR "sintodian"[tiab] OR "troperidol"[tiab] OR "xomolix"[tiab] OR "Aprepitant"[Mesh] OR "aprepitant"[tiab] OR "aponvie"[tiab] OR "aprepilor"[tiab] OR "atanto"[tiab] OR "cinvanti"[tiab] OR "emend"[tiab] OR "ht 001"[tiab] OR "ht001"[tiab] OR "htx 019"[tiab] OR "htx019"[tiab] OR "inepitant"[tiab] OR "l 754030"[tiab] OR "l754030"[tiab] OR "lorpitan"[tiab] OR "mk 0869"[tiab] OR "mk 869"[tiab] OR "mk0869"[tiab] OR "mk869"[tiab] OR "ono 7436"[tiab] OR "ono7436"[tiab] OR "tiperant"[tiab] OR "weg 232"[tiab] OR "weg232"[tiab] OR "rolapitant" [Supplementary Concept] OR "rolapitant*"[tiab] OR "sch 619734"[tiab] OR "sch619734"[tiab] OR "varubi"[tiab] OR "varuby"[tiab] OR "Dexamethasone"[Mesh] OR "dexamethason*"[tiab] OR "adrecort"[tiab] OR "adrenocot"[tiab] OR "aeroseb*"[tiab] OR "aflucoson*"[tiab] OR "alfalyl"[tiab] OR "anaflogistico*"[tiab] OR "aphtasolon"[tiab] OR "arcodexan*"[tiab] OR "artrosone"[tiab] OR "auxiron"[tiab] OR "azium"[tiab] OR "baycadron"[tiab] OR "bidexol"[tiab] OR "bisu ds"[tiab] OR "calonat"[tiab] OR "cebedex"[tiab] OR "cetadexon"[tiab] OR "colofoam"[tiab] OR "corsona"[tiab] OR "corsone"[tiab] OR "cortastat*"[tiab] OR "cortidex"[tiab] OR "cortidexason"[tiab] OR "cortidrona"[tiab] OR "cortidrone"[tiab] OR "cortisumman"[tiab] OR "dacortina fuerte"[tiab] OR "dacortine fuerte"[tiab] OR "dalalone*"[tiab] OR "danasone"[tiab] OR "de-sone la"[tiab] OR "decacortin"[tiab] OR "decadeltoson*"[tiab] OR "decaderm"[tiab] OR "decadion"[tiab] OR "decadran"[tiab] OR "decadron*"[tiab] OR "decaesadril"[tiab] OR "decagel"[tiab] OR "decaject*"[tiab] OR "decalix"[tiab] OR "decameth*"[tiab] OR "decasone"[tiab] OR "decaspray"[tiab] OR "decasterolone"[tiab] OR "decdan"[tiab] OR "decilone*"[tiab] OR "decofluor"[tiab] OR "dectancyl"[tiab] OR "dekacort"[tiab] OR "delladec"[tiab] OR "deltafluoren*"[tiab] OR "dergramin"[tiab] OR "deronil"[tiab] OR "desacort*"[tiab] OR "desadrene"[tiab] OR "desalark"[tiab] OR "desameton*"[tiab] OR "desigdron"[tiab] OR "dexa cortisyl"[tiab] OR "dexa dabrosan"[tiab] OR "dexa korti"[tiab] OR "dexa scherosan"[tiab] OR "dexa scherozon*"[tiab] OR "dexa-p"[tiab] OR "dexacen 4"[tiab] OR "dexachel"[tiab] OR "dexacollyre"[tiab] OR "dexacort*"[tiab] OR "dexadabroson"[tiab] OR "dexadecadrol"[tiab] OR "dexadrol"[tiab] OR "dexafarma"[tiab] OR "dexagel"[tiab] OR "dexagen"[tiab] OR "dexahelvacort"[tiab] OR "dexakorti"[tiab] OR "dexalien"[tiab] OR "dexaliquid geriasan"[tiab] OR "dexalocal"[tiab] OR "dexame"[tiab] OR "dexamecortin"[tiab] OR "dexameson*"[tiab] OR "dexametason*"[tiab] OR "dexameth*"[tiab] OR "dexamonozon"[tiab] OR "dexan"[tiab] OR "dexane"[tiab] OR "dexano"[tiab] OR "dexapolcort"[tiab] OR "dexapot"[tiab] OR "dexaschero*"[tiab] OR "dexason*"[tiab] OR "dexinoral"[tiab] OR "dexionil"[tiab] OR "dexmethsone"[tiab] OR "dexona"[tiab] OR "dexone*"[tiab] OR "dexpak*"[tiab] OR "dextelan"[tiab] OR "dextenza"[tiab] OR "dextrasone"[tiab] OR "dexycu*"[tiab] OR "dezone"[tiab] OR "dibasona"[tiab] OR "doxamethasone"[tiab] OR "esacortene"[tiab] OR "ex s1"[tiab] OR "exadion*"[tiab] OR "firmalone"[tiab] OR "fluormethyl*"[tiab] OR "fluormone"[tiab] OR "fluorocort"[tiab] OR "fluorodelta"[tiab] OR "fluoromethylprednisolone"[tiab] OR "fortecortin"[tiab] OR "gammacorten*"[tiab] OR "glensoludex"[tiab] OR "grosodexon*"[tiab] OR "hemady"[tiab] OR "hexadecadiol"[tiab] OR "hexadecadrol"[tiab] OR "hexadiol"[tiab] OR "hexadrol"[tiab] OR "isicort"[tiab] OR "isnacort"[tiab] OR "isopto*"[tiab] OR "isv 305"[tiab] OR "isv305"[tiab] OR "lokalison f"[tiab] OR "loverine"[tiab] OR "luxazone"[tiab] OR "marvidione"[tiab] OR "maxidex"[tiab] OR "mediamethasone"[tiab] OR "megacortin"[tiab] OR "mephameson*"[tiab] OR "metasolon*"[tiab] OR "methazon*"[tiab] OR "methylfluorprednisolone"[tiab] OR "metisone lafi"[tiab] OR "mexasone"[tiab] OR "millicorten*"[tiab] OR "mk 125"[tiab] OR "mk125"[tiab] OR "mymethasone"[tiab] OR "neoforderx"[tiab] OR "neofordex"[tiab] OR "nisomethasona"[tiab] OR "nodexon"[tiab] OR "novocort"[tiab] OR "nsc 34521"[tiab] OR "nsc34521"[tiab] OR "oftan-dexa"[tiab] OR "opticorten"[tiab] OR "opticortinol"[tiab] OR "oradexan"[tiab] OR "oradexon*"[tiab] OR "orgadrone"[tiab] OR "oto 104"[tiab] OR "oto104"[tiab] OR "ozurdex"[tiab] OR "perazone"[tiab] OR "pidexon"[tiab] OR "policort"[tiab] OR "posurdex"[tiab] OR "predni-f*"[tiab] OR "prednisolone f"[tiab] OR "prodexona"[tiab] OR "prodexone"[tiab] OR "sanamethasone"[tiab] OR "santenson"[tiab] OR "santeson"[tiab] OR "sawasone"[tiab] OR "sk 0503"[tiab] OR "sk0503"[tiab] OR "solurex*"[tiab] OR "spoloven"[tiab] OR "spt 2101"[tiab] OR "spt2101"[tiab] OR "sterasone"[tiab] OR "thilodexine"[tiab] OR "triamcimetil"[tiab] OR "vexamet"[tiab] OR "visumetazone"[tiab] OR "visumethazone"[tiab] OR "zeqmelit"[tiab] OR "Scopolamine"[Mesh] OR "scopolamin*"[tiab] OR "atrochin"[tiab] OR "atroquin"[tiab] OR "atroscine"[tiab] OR "Boro-Scopol"[tiab] OR "d l hyoscine"[tiab] OR "dl atroscine"[tiab] OR "hyosceine"[tiab] OR "hyoscine*"[tiab] OR "hysco"[tiab] OR "kimite-patch"[tiab] OR "Kwells"[tiab] OR "l epoxytropine tropate"[tiab] OR "n methylhyoscine"[tiab] OR "oscine"[tiab] OR "Scoburen"[tiab] OR "Scopace"[tiab] OR "scopalamine*"[tiab] OR "scopine tropate"[tiab] OR "Scopoderm*"[tiab] OR "transcop"[tiab] OR "Transderm*"[tiab] OR "Travacalm*"[tiab] OR "tropic acid ester with scopine"[tiab] OR "hyoscine N-oxide" [Supplementary Concept] OR "Ranitidine"[Mesh] OR "ranitidin*"[tiab] OR “acidex”[tiab] OR “aciloc”[tiab] OR “acloral”[tiab] OR “acran”[tiab] OR “aldin”[tiab] OR “alquen”[tiab] OR “anistal”[tiab] OR “antagonin”[tiab] OR “ardoral”[tiab] OR “atural”[tiab] OR “ausran”[tiab] OR “avintac”[tiab] OR “axoban”[tiab] OR “azantac”[tiab] OR “baroxal”[tiab] OR “biotidin”[tiab] OR “consec”[tiab] OR “coralen”[tiab] OR “cygran”[tiab] OR “duractin”[tiab] OR “eltidine”[tiab] OR “eu-ran”[tiab] OR “ezopta”[tiab] OR “galidrin”[tiab] OR “gastran”[tiab] OR “gastrial”[tiab] OR “gastridina”[tiab] OR “gastrosedol”[tiab] OR “hexer”[tiab] OR “histac”[tiab] OR “histak”[tiab] OR “hyzan”[tiab] OR “incid”[tiab] OR “iqfadina”[tiab] OR “kemoranin”[tiab] OR “kiradin”[tiab] OR “lumaren”[tiab] OR “lydin”[tiab] OR “microtid”[tiab] OR “midaven”[tiab] OR “nadine”[tiab] OR”neoceptin-r”[tiab] OR “novo-ranidine”[tiab] OR “pilorex”[tiab] OR “ponaltin”[tiab] OR “ptinolin”[tiab] OR “quantor”[tiab] OR “quicran”[tiab] OR “r-loc”[tiab] OR “radinat”[tiab] OR “radine”[tiab] OR “rafitaz”[tiab] OR “ranacid”[tiab] OR “rancet”[tiab] OR “randin”[tiab] OR “rani 2”[tiab] OR “ranial”[tiab] OR “raniben”[tiab] OR “ranibloc”[tiab] OR “ranicalm”[tiab] OR “ranidil”[tiab] OR “ranidine”[tiab] OR “ranidura”[tiab] OR “ranigast*”[tiab] OR “ranihexal”[tiab] OR “ranimex”[tiab] OR “ranin”[tiab] OR “raniogas”[tiab] OR “raniplex”[tiab] OR “ranisan”[tiab] OR “ranisen”[tiab] OR “ranitab”[tiab] OR “ranital”[tiab] OR “ranitax”[tiab] OR “ranitil”[tiab] OR “ranitin*”[tiab] OR “ranolta”[tiab] OR “rantac”[tiab] OR “rantacid”[tiab] OR “rantin”[tiab] OR “ranuber”[tiab] OR “ranzac”[tiab] OR “ratic”[tiab] OR “raticina”[tiab] OR “raxide”[tiab] OR “retamin”[tiab] OR “rolan”[tiab] OR “rosimol”[tiab] OR “simetac”[tiab] OR “sostril”[tiab] OR “tanidina”[tiab] OR “taural”[tiab] OR “terodul”[tiab] OR “toriol”[tiab] OR “ulcaid”[tiab] OR “ulceran”[tiab] OR “ulceranin”[tiab] OR “ulcex”[tiab] OR “ulcin”[tiab] OR “ulcocur”[tiab] OR “ulsal”[tiab] OR “ultak”[tiab] OR “ultidine”[tiab] OR “urantac”[tiab] OR “verlost”[tiab] OR “vesyca”[tiab] OR “vizerul”[tiab] OR “weichilin”[tiab] OR “weidos”[tiab] OR “xanidine”[tiab] OR “zantab”[tiab] OR “zantac*”[tiab] OR “zantadin”[tiab] OR “zantic”[tiab] OR “zinetac”[tiab] OR "Cisapride"[Mesh] OR "cisapride"[tiab] OR "acenalin"[tiab] OR "acpulsif"[tiab] OR "alimix*"[tiab] OR "alipride"[tiab] OR "cipride"[tiab] OR "cisamod"[tiab] OR "cisaprid*"[tiab] OR "cisapron"[tiab] OR "cisawal'"[tiab] OR "disflux"[tiab] OR "dizmoprida"[tiab] OR "enteropride"[tiab] OR "eriken"[tiab] OR "esorid"[tiab] OR "ethiprid"[tiab] OR "gastromet"[tiab] OR "guarposid"[tiab] OR "kineprid"[tiab] OR "kinestase"[tiab] OR "metison"[tiab] OR "motilar"[tiab] OR "palcid"[tiab] OR "prepulsid"[tiab] OR "presiston"[tiab] OR "prider"[tiab] OR "pridesia"[tiab] OR "prisic"[tiab] OR "propulsid*"[tiab] OR "propulsin"[tiab] OR "propulsit"[tiab] OR "pulsar (drug) "[tiab] OR "refluxin"[tiab] OR "risamol"[tiab] OR "saprid"[tiab] OR "stimulit"[tiab] OR "syspride"[tiab] OR "tono-cis"[tiab] OR "unamol"[tiab] OR "unipride"[tiab] OR "viprasen"[tiab] OR "vomipride"[tiab] OR "wepride"[tiab] OR "Cimetidine"[Mesh] OR "cimetidine*"[tiab] OR "aci-med"[tiab] OR "acibilin"[tiab] OR "acidnor"[tiab] OR "acinil"[tiab] OR "aidar"[tiab] OR "altramet"[tiab] OR "antag"[tiab] OR "apo-cimetidine"[tiab] OR "asaurex"[tiab] OR "azucimet"[tiab] OR "belomet"[tiab] OR "biomag"[tiab] OR "biomet"[tiab] OR "brumetidina"[tiab] OR "campanex"[tiab] OR "cemedin*"[tiab] OR "cementin"[tiab] OR "ciclem"[tiab] OR "cigamet"[tiab] OR "cignatin"[tiab] OR "ciket m"[tiab] OR "cimal"[tiab] OR "cimbene"[tiab] OR "cimedine"[tiab] OR "cimehexal"[tiab] OR "cimeldine"[tiab] OR "cimeloc"[tiab] OR "cimet*"[tiab] OR "cimetum"[tiab] OR "cimewell"[tiab] OR “cimlok"[tiab] OR “cimulcer"[tiab] OR “cinadine"[tiab] OR "cinulcus"[tiab] OR “cismetin"[tiab] OR "citidine"[tiab] OR "citius"[tiab] OR "contracid"[tiab] OR "corsamet"[tiab] OR "cylock"[tiab] OR "cytine"[tiab] OR “dispamet"[tiab] OR “duomet"[tiab] OR “dyspamet"[tiab] OR “edalene"[tiab] OR “eladene"[tiab] OR “erlmetin"[tiab] OR “eureceptor"[tiab] OR “fremet"[tiab] OR “gastab"[tiab] OR “gastidine"[tiab] OR “gastrobitan"[tiab] OR “gastroprotect"[tiab] OR “gawei"[tiab] OR “getidin"[tiab] OR “haldin"[tiab] OR “hexamet"[tiab] OR “himetin"[tiab] OR “histodil"[tiab] OR “inesfay"[tiab] OR “lenamet*"[tiab] OR “lock 2"[tiab] OR “magicul"[tiab] OR “manomet"[tiab] OR “maritidine"[tiab] OR “med-gastramet"[tiab] OR “meticon"[tiab] OR “neutromed"[tiab] OR “neutronorm*"[tiab] OR “novocimetine"[tiab] OR “nulcer"[tiab] OR “peptol"[tiab] OR “piovalen"[tiab] OR “pondarmett"[tiab] OR “powegon"[tiab] OR “promet”:ti,ab,kw OR “sanmetidin”:ti,ab,kw OR “secapine”:ti,ab,kw OR “senoba”:ti,ab,kw OR “sertidine"[tiab] OR “shintamet"[tiab] OR “siamidine"[tiab] OR “sigmetadine"[tiab] OR “simaglen"[tiab] OR “stogamet"[tiab] OR “stomakon"[tiab] OR “stomedine"[tiab] OR “stomet"[tiab] OR “tagagel"[tiab] OR “tagamet*"[tiab] OR “tagma"[tiab] OR “tametin"[tiab] OR “tobymet"[tiab] OR “ulcedin*"[tiab] OR “ulcemet"[tiab] OR “ulcenon"[tiab] OR “ulcerfen"[tiab] OR “ulcidine"[tiab] OR “ulcim*"[tiab] OR “ulcodina"[tiab] OR “ulcolind h2"[tiab] OR “ulcomedina"[tiab] OR “ulcomet"[tiab] OR “ulcumet"[tiab] OR “ulsikur"[tiab] OR “umamett"[tiab] OR “weisdin"[tiab] OR “xepamet"[tiab] OR “zymerol"[tiab])

Antiepileptics

("Anticonvulsants"[Mesh] OR "anticonvuls*"[tiab] OR "anti-convuls*"[tiab] OR "AED"[tiab] OR "AEDs"[tiab] OR "antiepilep*"[tiab] OR "anti-epilep*"[tiab] OR "anti-seizure"[tiab] OR "antiseizure"[tiab] OR "Benzodiazepines"[Mesh] OR "benzodiazepin*"[tiab] OR "Barbiturates"[Mesh] OR "barbiturat*"[tiab] OR "brivaracetam" [Supplementary Concept] OR "brivaracetam" [tiab] OR "Briviact"[tiab] OR "brivlera"[tiab] OR "nubriveo"[tiab] OR "rikelta"[tiab] OR "Midazolam"[Mesh] OR "midazolam*"[tiab] OR "Dormicum"[tiab] OR "Dormire"[tiab] OR "Epistatus"[tiab] OR "Fulsed"[tiab] OR "Garen"[tiab] OR "Hypnovel"[tiab] OR "Ipnovel"[tiab] OR "Versed"[tiab] OR "Dalam"[tiab] OR "Nocturna"[tiab] OR "Setam"[tiab] OR "Propofol"[Mesh] OR "Propofol*"[tiab] OR "Disoprofol"[tiab] OR "Diprivan"[tiab] OR "Disoprivan"[tiab] OR "Fresofol"[tiab] OR "Ivofol"[tiab] OR "Recofol"[tiab] OR "Aquafol"[tiab] OR "Recofol"[tiab] OR "Anepol"[tiab] OR "Hypro"[tiab] OR "Lipuro"[tiab] OR "Plofed"[tiab] OR "Profol"[tiab] OR "Propofil"[tiab] OR "Propolipid"[tiab] OR "Propovan"[tiab] OR "Propoven"[tiab] OR "Provive"[tiab] OR "Clonazepam"[Mesh] OR "clonazepam*"[tiab] OR "Rivotril"[tiab] OR "Klonopin"[tiab] OR "Antelepsin"[tiab] OR "Diazepam"[Mesh] OR "diazepam*"[tiab] OR "Diazemuls"[tiab] OR "Faustan"[tiab] OR "Valium"[tiab] OR "Seduxen"[tiab] OR "Sibazon"[tiab] OR "Stesolid"[tiab] OR "Apaurin"[tiab] OR "Relanium"[tiab] OR "Carbamazepine"[Mesh] OR "carbamazepin*"[tiab] OR "Carbazepin"[tiab] OR "Epitol"[tiab] OR "Finlepsin"[tiab] OR "Neurotol"[tiab] OR "Tegretol"[tiab] OR "Amizepine"[tiab] OR "Valproic Acid"[Mesh] OR "valproic*"[tiab] OR "Divalproex*"[tiab] OR "Depakene"[tiab] OR "Depakine"[tiab] OR "Convulsofin"[tiab] OR "Depakote"[tiab] OR "Dipropyl Acetate"[tiab] OR "valproate*"[tiab] OR "Vupral"[tiab] OR "Propylisopropylacetic Acid"[tiab] OR "Ergenyl"[tiab] OR "Levetiracetam"[Mesh] OR "levetiracetam*"[tiab] OR "Etiracetam"[tiab] OR "Keppra"[tiab] OR "Phenobarbital"[Mesh] OR "phenobarbit*"[tiab] OR "Phenylbarbital"[tiab] OR "Phenylethylbarbituric Acid"[tiab] OR "Phenemal"[tiab] OR "Phenobarbitone"[tiab] OR "Hysteps"[tiab] OR "Luminal"[tiab] OR "Gardenal"[tiab] OR "Lamotrigine"[Mesh] OR "lamotrigi*"[tiab] OR "Lamictal"[tiab] OR "Crisomet"[tiab] OR "Lamiktal"[tiab] OR "Labileno"[tiab] OR "Lacosamide"[Mesh] OR "lacosamide"[tiab] OR "Vimpat"[tiab] OR "Cannabidiol"[Mesh] OR "cannabidiol"[tiab] OR "Epidiolex"[tiab] OR "Cenobamate" [Supplementary Concept] OR "cenobamat*" [tiab] OR "XCOPRI" [tiab] OR "Clobazam"[Mesh] OR "clobazam*"[tiab] OR "Onfi"[tiab] OR "Urbanyl"[tiab] OR "Frisium"[tiab] OR "eslicarbazepin*" [Supplementary Concept] OR "eslicarbazepine" [tiab] OR "Ethosuximide"[Mesh] OR "ethosuximid*"[tiab] OR "ethosuccimid*"[tiab] OR "ethylmethylsuccimide"[tiab] OR "Ethymal"[tiab] OR "Zarontin"[tiab] OR "Petnidan"[tiab] OR "Pyknolepsinum"[tiab] OR "Suksilep"[tiab] OR "Suxilep"[tiab] OR "Emeside"[tiab] OR "Etosuximida Faes"[tiab] OR "ezogabine" [Supplementary Concept] OR "ezogabine"[tiab] OR "retigabin*" [tiab] OR "Potiga" [tiab] OR "Felbamate"[Mesh] OR "felbamat*"[tiab] OR "Taloxa"[tiab] OR "Felbatol"[tiab] OR "Felbamyl"[tiab] OR "Fenfluramine"[Mesh] OR "fenfluramin*"[tiab] OR "Pondimin"[tiab] OR "Isomeride"[tiab] OR "Fintepla"[tiab] OR "Gabapentin"[Mesh] OR "gabapenti*"[tiab] OR "NovoGabapentin"[tiab] OR "ApoGabapentin"[tiab] OR "Neurontin"[tiab] OR "Convalis"[tiab] OR "Lacosamide"[Mesh] OR "lacosamid*"[tiab] OR "Vimpat"[tiab] OR "Lorazepam"[Mesh] OR "lorazepam*"[tiab] OR "Ativan"[tiab] OR "Orfidal Wyeth"[tiab] OR "Témesta"[tiab] OR "Tolid"[tiab] OR "Donix"[tiab] OR "Duralozam"[tiab] OR "Durazolam"[tiab] OR "Idalprem"[tiab] OR "Laubeel"[tiab] OR "Lorazep Von Ct"[tiab] OR "Novo-Lorazem"[tiab] OR "Nu-Loraz"[tiab] OR "Sedicepan"[tiab] OR "Sinestron"[tiab] OR "Somagerol"[tiab] OR "Temesta"[tiab] OR "Oxcarbazepine"[Mesh] OR "oxcarbazepine*"[tiab] OR "Trileptal"[tiab] OR "Timox"[tiab] OR "perampanel" [Supplementary Concept] OR "perampanel*" [tiab] OR "Fycompa" [tiab] OR "Phenytoin"[Mesh] OR "phenytoin*"[tiab] OR "Fenitoin"[tiab] OR "Diphenylhydantoin"[tiab] OR "Difenin"[tiab] OR "Dihydan"[tiab] OR "Phenhydan"[tiab] OR "Epamin"[tiab] OR "Epanutin"[tiab] OR "Hydantol"[tiab] OR "Sodium Diphenylhydantoinate"[tiab] OR "Antisacer"[tiab] OR "Dilantin"[tiab] OR "Pregabalin"[Mesh] OR "pregabalin*"[tiab] OR "Lyrica"[tiab] OR "Primidone"[Mesh] OR "primidon*"[tiab] OR "primidon*"[tiab] OR "Liskantin"[tiab] OR "Mylepsinum"[tiab] OR "Resimatil"[tiab] OR "Desoxyphenobarbital"[tiab] OR "Mizodin"[tiab] OR "Mysoline"[tiab] OR "Primaclone"[tiab] OR "Sertan"[tiab] OR "Misodine"[tiab] OR "rufinamide" [Supplementary Concept] OR "rufinamid*" [tiab] OR "Inovelon" [tiab] OR "stiripentol" [Supplementary Concept] OR "stiripentol*" [tiab] OR "Diacomit" [tiab] OR "Tiagabine"[Mesh] OR "tiagabin*"[tiab] OR "Gabitril"[tiab] OR "Topiramate"[Mesh] OR "topiramat*"[tiab] OR "Epitomax"[tiab] OR "Topamax"[tiab] OR "Vigabatrin"[Mesh] OR "vigabatrin*"[tiab] OR "Sabrilex"[tiab] OR "Sabril"[tiab] OR "Zonisamide"[Mesh] OR "zonisamid*"[tiab] OR "Zonegran"[tiab])

Antidepressants

("Antidepressive Agents"[Mesh] OR "antidepressive*"[tiab] OR "antidepressant*"[tiab] OR "antidepression*"[tiab] OR "anti depressant*"[tiab] OR "Serotonin Uptake Inhibitors"[Mesh] OR "Adrenergic Uptake Inhibitors"[Mesh] OR "Serotonin and Noradrenaline Reuptake Inhibitors"[Mesh] OR "Mirtazapine"[Mesh] OR "Bupropion"[Mesh] OR "Duloxetine Hydrochloride"[Mesh] OR "Venlafaxine Hydrochloride"[Mesh] OR "Sertraline"[Mesh] OR "Fluoxetine"[Mesh] OR "Paroxetine"[Mesh] OR "Fluvoxamine"[Mesh] OR "Escitalopram"[Mesh] OR "Citalopram"[Mesh] OR "Trazodone"[Mesh] OR "serotonin reuptake inhibitor*"[tiab] OR "serotonin specific reuptake inhibitor*"[tiab] OR "serotonin uptake inhibitor*"[tiab] OR "5-hydroxytryptamine uptake inhibitor*"[tiab] OR "5-HT uptake inhibitor*"[tiab] OR "SSRI*"[tiab] OR "second-generation antidepress*"[tiab] OR "atypical antidepress*"[tiab] OR "serotonin and noradrenaline reuptake inhibitor*"[tiab] OR "serotonin and noradrenaline uptake inhibitor*"[tiab] OR "serotonin noradrenaline reuptake inhibitor*"[tiab] OR "serotonin noradrenaline uptake inhibitor*"[tiab] OR "dual reuptake inhibitor*"[tiab] OR "dual uptake inhibitor*"[tiab] OR "citalopram"[tiab] OR "escitalopram"[tiab] OR "fluvoxamine"[tiab] OR "paroxetine"[tiab] OR "fluoxetine"[tiab] OR "sertraline"[tiab] OR "serotonin and norepinephrine reuptake inhibitor*"[tiab] OR "serotonin and norepinephrine uptake inhibitor*"[tiab] OR "serotonin norepinephrine reuptake inhibitor*"[tiab] OR "serotonin norepinephrine uptake inhibitor*"[tiab] OR "SNRI*"[tiab] OR "SSNRI*"[tiab] OR "venlafaxine"[tiab] OR "duloxetine"[tiab] OR "mirtazapine"[tiab] OR "trazodone"[tiab] OR "bupropion"[tiab] OR "amfebutamone"[tiab] OR "Nortriptyline"[Mesh] OR "nortriptyline"[tiab] OR "desitriptyline"[tiab] OR "desmethylamitriptylin"[tiab] OR "Allegron"[tiab] OR "Aventyl"[tiab] OR "Paxtibi"[tiab] OR "Nortrilen"[tiab] OR "Pamelor"[tiab] OR "Norfenazin"[tiab] OR "Amitriptyline"[Mesh] OR "amitrip*"[tiab] OR "Tryptine"[tiab] OR "Amineurin"[tiab] OR "Amitrip"[tiab] OR "Amitrol"[tiab] OR "Anapsique"[tiab] OR "Damilen"[tiab] OR "Domical"[tiab] OR "Laroxyl"[tiab] OR "Lentizol"[tiab] OR "Novoprotect"[tiab] OR "Saroten"[tiab] OR "Sarotex"[tiab] OR "Syneudon"[tiab] OR "Triptafen"[tiab] OR "Endep"[tiab] OR "Tryptizol"[tiab] OR "Elavil"[tiab] OR "Tryptanol"[tiab] OR "Clomipramine"[Mesh] OR "clomipramine*"[tiab] OR "chlomipramine"[tiab] OR "chlorimipramine"[tiab] OR "Hydiphen"[tiab] OR "Anafranil"[tiab] OR "Doxepin"[Mesh] OR "doxepi*"[tiab] OR "Deptran"[tiab] OR "Desidox"[tiab] OR "Doneurin"[tiab] OR "Espadox"[tiab] OR "Mareen"[tiab] OR "Prudoxin"[tiab] OR "Quitaxon"[tiab] OR "Sinequan"[tiab] OR "Sinquan"[tiab] OR "Zonalon"[tiab] OR "Xepin"[tiab] OR "Aponal"[tiab] OR "Xepin"[tiab] OR "Dothiepin"[Mesh] OR "dothiepin"[tiab] OR "dothiepin*"[tiab] OR "dosulepin"[tiab] OR "Prothiaden"[tiab] OR "Maprotiline"[Mesh] OR "Maprotilin*"[tiab] OR "Novo-Maprotiline"[tiab] OR "Dibencycladine"[tiab] OR "Psymion"[tiab] OR "Ludiomil"[tiab] OR "Maprolu"[tiab] OR "Mirpan"[tiab] OR "Deprilept"[tiab] OR "Imipramine"[Mesh] OR "imipramine*"[tiab] OR "norchlorimipramine"[tiab] OR "imidobenzyle"[tiab] OR "Imizin"[tiab] OR "Tofranil"[tiab] OR "Janimine"[tiab] OR "Melipramine"[tiab] OR "Pryleugan"[tiab])

Lithium

("Lithium"[Mesh] OR "Lithium Compounds"[Mesh] OR "Antimanic Agents"[Mesh] OR "lithium*"[tiab] OR "antimanic*"[tiab] OR "Lithium Carbonate"[Mesh] OR "camcolit"[tiab] OR "candamide"[tiab] OR "carbolit*"[tiab] OR "carbonate lithium"[tiab] OR "ceglution*"[tiab] OR "contemnol"[tiab] OR "cp 15, 467 61"[tiab] OR "cp 15467 61"[tiab] OR "cp15, 467 61"[tiab] OR "cp15467 61"[tiab] OR "duralith"[tiab] OR "eskalith*"[tiab] OR "hynorex*"[tiab] OR "lentolith"[tiab] OR "licab"[tiab] OR "licarb*"[tiab] OR "lidin"[tiab] OR "limas"[tiab] OR "linthane"[tiab] OR "liskonium"[tiab] OR "liskonum"[tiab] OR "lithane"[tiab] OR "litheum 300"[tiab] OR "lithicarb"[tiab] OR "lithionate"[tiab] OR "lithmax"[tiab] OR "lithobid"[tiab] OR "lithocap"[tiab] OR "lithocarb"[tiab] OR "lithonate"[tiab] OR "lithotabs"[tiab] OR "litilent"[tiab] OR "litocarb"[tiab] OR "maniprex"[tiab] OR "neurolepsin"[tiab] OR "nsc 16895"[tiab] OR "nsc 16985"[tiab] OR "phanate"[tiab] OR "plenur"[tiab] OR "priadel*"[tiab] OR "quilonium-r"[tiab] OR "quilonorm*"[tiab] OR "quilonum*"[tiab] OR "teralithe"[tiab] OR "theralite"[tiab] OR "theralithe lp"[tiab])

Stimulants

("Methylphenidate"[Mesh] OR "methylphenidate*"[tiab] OR "methylphenydate*"[tiab] OR "methylphenindate*"[tiab] OR "methylfenidaat"[tiab] OR "methylfenidate"[tiab] OR "methyl phenidate"[tiab] OR "Addepta*"[tiab] OR "adhansia*"[tiab] OR "affenid*"[tiab] OR "aptensio*"[tiab] OR "atenza*"[tiab] OR "attenta"[tiab] OR "benjorna"[tiab] OR "biphentin"[tiab] OR "centedrin"[tiab] OR "concerta*"[tiab] OR "cotempla*"[tiab] OR "daytrana"[tiab] OR "delmosart"[tiab] OR "difumenil"[tiab] OR "equasym*"[tiab] OR "exattent*"[tiab] OR "focusim xl"[tiab] OR "foquest"[tiab] OR "jornay*"[tiab] OR "kinecteen"[tiab] OR "kixel*"[tiab] OR "matoride xl"[tiab] OR "medanef"[tiab] OR "medicebran"[tiab] OR "medikinet*"[tiab] OR "mefinad"[tiab] OR "metadate*"[tiab] OR "methylin*"[tiab] OR "methypatch"[tiab] OR "methysym*"[tiab] OR "metyrol*"[tiab] OR "motiron"[tiab] OR "penid"[tiab] OR "phenidyl*"[tiab] OR "quasym*"[tiab] OR "quillichew*"[tiab] OR "quillivant*"[tiab] OR "relexxii"[tiab] OR "rilatin*"[tiab] OR "ritalin*"[tiab] OR "ritaphen"[tiab] OR "rubicrono"[tiab] OR "rubifen*"[tiab] OR "tranquilyn"[tiab] OR "tsentedrin"[tiab] OR "xaggitin xl"[tiab] OR "xenidate xl"[tiab])

Antihistamines

("Histamine Antagonists"[Mesh] OR "antihistamine*"[tiab] OR "anti-histamine*"[tiab] OR "antihistaminic*"[tiab] OR "anti-histaminic*"[tiab] OR "histamine antagonist*"[tiab] OR "histamine receptor antagonist*"[tiab] OR "histamine block*"[tiab] OR "histamine receptor block*"[tiab] OR "histamine H1 antagonist*"[tiab] OR "histamine H2 antagonist*"[tiab] OR "histamine H3 antagonist*"[tiab] OR "histamine H4 antagonist*"[tiab] OR "histamine H1 receptor antagonist*"[tiab] OR "histamine H2 receptor antagonist*"[tiab] OR "histamine H3 receptor antagonist*"[tiab] OR "histamine H4 receptor antagonist*"[tiab] OR "H1 antagonist*"[tiab] OR "H2 antagonist*"[tiab] OR "H3 antagonist*"[tiab] OR "H4 antagonist*"[tiab] OR "H1 receptor antagonist*"[tiab] OR "H2 receptor antagonist*"[tiab] OR "H3 receptor antagonist*"[tiab] OR "H4 receptor antagonist*"[tiab] OR "histamine H1 receptor block*"[tiab] OR "histamine H2 receptor block*"[tiab] OR "histamine H3 receptor block*"[tiab] OR "histamine H4 receptor block*"[tiab] OR "histamine H1 block*"[tiab] OR "histamine H2 block*"[tiab] OR "histamine H3 block*"[tiab] OR "histamine H4 block*"[tiab] OR "Hydroxyzine"[Mesh] OR "hydroxyzin*"[tiab] OR "Atarax*"[tiab] OR "Aterax"[tiab] OR "Attarax"[tiab] OR "Bestalin"[tiab] OR "Bobsule"[tiab] OR "Centilax"[tiab] OR "Cerax"[tiab] OR "Darax"[tiab] OR "Disron"[tiab] OR "Dormirex"[tiab] OR "Durrax"[tiab] OR "Efidac"[tiab] OR "Hiderax"[tiab] OR "Hizin"[tiab] OR "Hyzine"[tiab] OR "Idroxizina"[tiab] OR "Iremofar"[tiab] OR "Iterax"[tiab] OR "Orgatrax"[tiab] OR "Otarex"[tiab] OR "Paxistil"[tiab] OR "Phymorax"[tiab] OR "Postarax"[tiab] OR "Prurid"[tiab] OR "Qualidrozine"[tiab] OR "Quiess"[tiab] OR "R-Rax"[tiab] OR "Tran-Q"[tiab] OR "Trandozine"[tiab] OR "Tranquijust"[tiab] OR "Ucerax"[tiab] OR "Unamine"[tiab] OR "Vistacot"[tiab] OR "Vistaject-50"[tiab] OR "Vistaril*"[tiab] OR "acrivastine" [Supplementary Concept] OR "acrivastine*"[tiab] OR "Prolert"[tiab] OR "Semprex"[tiab] OR "azelastine" [Supplementary Concept] OR "azelastin*"[tiab] OR "Acatar Allergy"[tiab] OR "Afluon*"[tiab] OR "Alastine"[tiab] OR "Alerdual"[tiab] OR "Alergodil"[tiab] OR "Allergo-azelind"[tiab] OR "Allergodil*"[tiab] OR "Allergodrop"[tiab] OR "Allergospray"[tiab] OR "Allespray"[tiab] OR "Allestin"[tiab] OR "Amelor"[tiab] OR "Antalerg"[tiab] OR "Astelin"[tiab] OR "Astepro*"[tiab] OR "Azedil"[tiab] OR "Azel-drop*"[tiab] OR "Azela vision*"[tiab] OR "Azelamed"[tiab] OR "Azelastin*"[tiab] OR "Azelavision*"[tiab] OR "Azella"[tiab] OR "Azelsan"[tiab] OR "Azep*"[tiab] OR "Carelastin"[tiab] OR "Corifina"[tiab] OR "Lasticom"[tiab] OR "Lastin*"[tiab] OR "Loxin"[tiab] OR "Oculastin"[tiab] OR "Optilast"[tiab] OR "Optivar"[tiab] OR "Pollival"[tiab] OR "Proallergodil"[tiab] OR "Radethacin"[tiab] OR "Radethazin"[tiab] OR "Rhinolast*"[tiab] OR "Rinelaz"[tiab] OR "Snizdil"[tiab] OR "Tebarat"[tiab] OR "Visuzel"[tiab] OR "Vividrin akut*"[tiab] OR "Vivispray"[tiab] OR "azatadine"[Supplementary Concept] OR "azatadin*"[tiab] OR "Idulamine"[tiab] OR "Idulian"[tiab] OR "Lergocil"[tiab] OR "Nalomet"[tiab] OR "Optimine"[tiab] OR "Verben"[tiab] OR "Zadine"[tiab] OR "Brompheniramine"[Mesh] OR "brompheniramin*"[tiab] OR "para-bromdylamine"[tiab] OR "p-Bromdylamine*"[tiab] OR "Babycold"[tiab] OR "Bomex"[tiab] OR "Bomine"[tiab] OR "Brombay"[tiab] OR "Bromdylamine"[tiab] OR "Bromfeniramine"[tiab] OR "Brommine"[tiab] OR "Bromoprophenpyridamine"[tiab] OR "Bromprophenpyridamine"[tiab] OR "Broramin"[tiab] OR "Brovex*"[tiab] OR "Codimal*"[tiab] OR "Colhist"[tiab] OR "Dimegan"[tiab] OR "Dimetane*"[tiab] OR "Dimigan"[tiab] OR "Dimotane"[tiab] OR "Dimetapp*"[tiab] OR "Ilvin"[tiab] OR "Lodrane*"[tiab] OR "Nasahist*"[tiab] OR "Nd stat"[tiab] OR "Oraminic-2"[tiab] OR "Parabromdylamine"[tiab] OR "Veltane"[tiab] OR "carbinoxamin*"[tiab] OR "paracarbinoxamin*"[tiab] OR "Allergefon" [tiab] OR "Polistine" [tiab] OR "Pyrate" [tiab] OR "Shelton" [tiab] OR "Tilford" [tiab] OR "Twiston" [tiab] OR "Ziriton" [tiab] OR "cetirizin*"[tiab] OR "Acidrine"[tiab] OR "Actifed allergie"[tiab] OR "Adezio"[tiab] OR "Agelmin"[tiab] OR "Alairgix*"[tiab] OR "Alercet"[tiab] OR "Alerid"[tiab] OR "Alerlisin"[tiab] OR "Alertop"[tiab] OR "Alerviden"[tiab] OR "Aletir"[tiab] OR "Alled"[tiab] OR "Allertec"[tiab] OR "Alltec"[tiab] OR "Alzytec"[tiab] OR "Bell's healthcare allergy and hayfever relief"[tiab] OR "Benaday"[tiab] OR "Benadryl*"[tiab] OR "Betarhin"[tiab] OR "Cabal"[tiab] OR "Cerazine"[tiab] OR "Cerini"[tiab] OR "Cerotec"[tiab] OR "Cesta"[tiab] OR "Cetalerg"[tiab] OR "Piriteze*"[tiab] OR "Virlix"[tiab] OR "Zetir"[tiab] OR "Zyrtec"[tiab] OR "Reactine"[tiab] OR "Voltric*"[tiab] OR "Zirtek"[tiab] OR "Alerlisin"[tiab] OR "Cetalerg"[tiab] OR "Ceterifug"[tiab] OR "Cethis"[tiab] OR "Cetimin"[tiab] OR "Cetin"[tiab] OR "Cetirax"[tiab] OR "Cetirin"[tiab] OR "Ceti TAD"[tiab] OR "Ceti-Puren"[tiab] OR "Cetiderm"[tiab] OR "Cetidura"[tiab] OR "Cetil von ct"[tiab] OR "CetiLich"[tiab] OR "Cetirigamma"[tiab] OR "Cetirlan"[tiab] OR "Cetrimed"[tiab] OR "Cetrine"[tiab] OR "Cetrizet"[tiab] OR "Cetrizin"[tiab] OR "Cetymin"[tiab] OR "Chlorphed"[tiab] OR "Cistamine"[tiab] OR "Deallergy"[tiab] OR "Drill allergie"[tiab] OR "Em pharma daily hayfever"[tiab] OR "Falergi"[tiab] OR "Finallerg"[tiab] OR "Formistin"[tiab] OR "Generit"[tiab] OR "Histazine"[tiab] OR "Histica"[tiab] OR "Incidal*"[tiab] OR "Lergium"[tiab] OR "Livoreactine"[tiab] OR "Manx allergy and hayfever relief"[tiab] OR "Nosemin"[tiab] OR "Nosmin"[tiab] OR "Ozen"[tiab] OR "Piriteze allergy"[tiab] OR "Pollenase"[tiab] OR "Pollenshield hayfever relief"[tiab] OR "Prixlae"[tiab] OR "Quzyttir"[tiab] OR "Qzytir"[tiab] OR "Raingen"[tiab] OR "Razene"[tiab] OR "Reactine"[tiab] OR "Rhizin"[tiab] OR "Risima"[tiab] OR "Ritecam"[tiab] OR "Ryvel"[tiab] OR "Ryzen"[tiab] OR "Sancotec"[tiab] OR "Selitex"[tiab] OR "Setizin"[tiab] OR "Simtec"[tiab] OR "Sutac"[tiab] OR "Symitec"[tiab] OR "Terizin"[tiab] OR "Terzine"[tiab] OR "Vick-zyrt"[tiab] OR "Virlix"[tiab] OR "Wockhardt allergy and hayfever relief"[tiab] OR "Zenriz"[tiab] OR "Zensil"[tiab] OR "Zeran"[tiab] OR "Zertine"[tiab] OR "Zerviate"[tiab] OR "Zetir"[tiab] OR "Zicet"[tiab] OR "Zinex"[tiab] OR "Ziralton"[tiab] OR "Zyrtec*"[tiab] OR "Zirtec*"[tiab] OR "Zirtek*"[tiab] OR "Zirtin"[tiab] OR "Zyllergy"[tiab] OR "Zymed"[tiab] OR "Zyrac"[tiab] OR "Zyrazine"[tiab] OR "Zyrcon"[tiab] OR "Zyrlex"[tiab] OR "Zyrtek"[tiab] OR "Chlorpheniramine"[Mesh] OR "chlorpheniramin*"[tiab] OR "chlorprophenpyridamine"[tiab] OR "chlorphenamin*"[tiab] OR "Allergex"[tiab] OR "Allergicin"[tiab] OR "Allergosan"[tiab] OR "Amichlorphen"[tiab] OR "Aller-Chlor"[tiab] OR "Chlor-100"[tiab] OR "chlorfeniramine"[tiab] OR "chloropheniramine"[tiab] OR "chloroprofenpyridamine"[tiab] OR "chlorphenamin*"[tiab] OR "chlorpheneramine"[tiab] OR "chlorphenirame"[tiab] OR "chlorpheniramine"[tiab] OR "chlorphenyramine"[tiab] OR "chlorphenizamine"[tiab] OR "chlorprofenpyridamine"[tiab] OR "chlorprophenpyridamide"[tiab] OR "chlorprophenpyridamine"[tiab] OR "Haymine"[tiab] OR "Histafen timed capsule"[tiab] OR "Phenamin retard"[tiab] OR "Trimeton"[tiab] OR "Chlo-Amine"[tiab] OR "Chlorpro"[tiab] OR "Chlorspan"[tiab] OR "Chlortab"[tiab] OR "Chlor-Tripolon"[tiab] OR "Efidac"[tiab] OR "Kloromin*"[tiab] OR "Piriton"[tiab] OR "Teldrin"[tiab] OR "Clemastine"[Mesh] OR "clemastin*"[tiab] OR "Mecloprodin"[tiab] OR "Meclastine"[tiab] OR "Neclastine"[tiab] OR "Tavist"[tiab] OR "Tavegyl*"[tiab] OR "Tavegil*"[tiab] OR "Cinnarizine"[Mesh] OR "cinna*"[tiab] OR "Aplactan"[tiab] OR "Aplexal"[tiab] OR "Apomitere"[tiab] OR "Apotomin"[tiab] OR "Artate"[tiab] OR "Carecin"[tiab] OR "Cerebolan"[tiab] OR "Cerepar"[tiab] OR "Cibine"[tiab] OR "Cimarizine"[tiab] OR "Cinabioquim"[tiab] OR "Cinaperazine"[tiab] OR "Cinazière"[tiab] OR "Cinazyn"[tiab] OR "Cinnipirine"[tiab] OR "Cinniprine"[tiab] OR "Cisaken"[tiab] OR "Corathiem"[tiab] OR "Denapol"[tiab] OR "Dimitron*"[tiab] OR "Eglen"[tiab] OR "Giganten"[tiab] OR "Glanil"[tiab] OR "Hilactan"[tiab] OR "Ixertol"[tiab] OR "Katoseran"[tiab] OR "Labyrin"[tiab] OR "Lazeta"[tiab] OR "Marisan"[tiab] OR "Midronal"[tiab] OR "Mitronal"[tiab] OR "Olamin"[tiab] OR "Processine"[tiab] OR "Roin"[tiab] OR "Sedatromin"[tiab] OR "Sepan"[tiab] OR "Siptazin"[tiab] OR "Spaderizine"[tiab] OR "Stugeron*"[tiab] OR "Stutgeron"[tiab] OR "Stutgin"[tiab] OR "Cyclizine"[Mesh] OR "cyclizin*"[tiab] OR "Collox"[tiab] OR "Echnatol"[tiab] OR "Gotur"[tiab] OR "Marazine"[tiab] OR "Marezine"[tiab] OR "Marzine"[tiab] OR "Neo Devomit"[tiab] OR "Reisfit"[tiab] OR "Valoid"[tiab] OR "Cyproheptadine"[Mesh] OR "cyproheptadin*"[tiab] OR “crypoheptadin*"[tiab] OR “cryoheptidin*"[tiab] OR “Adekin"[tiab] OR "Antergan"[tiab] OR “Antisemin"[tiab] OR “Apeton*"[tiab] OR “Ciplactin"[tiab] OR “Cipractin"[tiab] OR “Cyheptine"[tiab] OR “Cylat"[tiab] OR “Cypraheptidine"[tiab] OR “Cypro h"[tiab] OR “Cyproatin"[tiab] OR “Cyprogin"[tiab] OR “Cyprohaptadine"[tiab] OR “Cyproheptadiene"[tiab] OR “Cyproheptadin*"[tiab] OR “Cypromin"[tiab] OR “Cyprono"[tiab] OR “Cyprosian"[tiab] OR “Cytadine"[tiab] OR “Ciproeptadine"[tiab] OR “Ciproral"[tiab] OR “Ciprotol"[tiab] OR “Ciprovit-a"[tiab] OR “Cytadine"[tiab] OR "Dihexazin"[tiab] OR “Ennamax"[tiab] OR “Glocyp"[tiab] OR “Heptasan"[tiab] OR “Ifrasal"[tiab] OR “Istam-far"[tiab] OR “Klarivitina"[tiab] OR “Kulinet"[tiab] OR “Nuran"[tiab] OR “Periacti*"[tiab] OR “Peritol"[tiab] OR “Petina"[tiab] OR “Pilian"[tiab] OR “Pronicy"[tiab] OR “Sinapdin"[tiab] OR “Trimetabol"[tiab] OR “Vinorex"[tiab] OR “Viternum"[tiab] OR "dexchlorpheniramine" [Supplementary Concept] OR "dexchlorpheniramin*"[tiab] OR "dextrochlorpheniramin*"[tiab] OR "d chlorpheniramin*"[tiab] OR "Isomerine"[tiab] OR "Dexclor"[tiab] OR "Phendextro"[tiab] OR "Polaramin*"[tiab] OR "Polargen TD"[tiab] OR "Polaronil"[tiab] OR "Dimenhydrinate"[Mesh] OR "dimenhydrinate*"[tiab] OR "Amosyt"[tiab] OR "Anautin*"[tiab] OR "Andramine*"[tiab] OR "Andrumin"[tiab] OR "Antemin"[tiab] OR "Antimo"[tiab] OR "Apo-dimenhydrinate"[tiab] OR "Aviomarin"[tiab] OR "Biodramina"[tiab] OR "Bonaling-a"[tiab] OR "Calm-X"[tiab] OR "Cinfamar"[tiab] OR "Contramareo"[tiab] OR "Chloranautine"[tiab] OR "Chloronautine"[tiab] OR "Demodenal"[tiab] OR "Denim"[tiab] OR "Detensor"[tiab] OR "Diamarin"[tiab] OR "Dimate"[tiab] OR "Dimen"[tiab] OR "Dimenate"[tiab] OR "Dimenhydrinat"[tiab] OR "Dimin"[tiab] OR "Dimetabs"[tiab] OR "Dinate"[tiab] OR "DMH"[tiab] OR "diphenhydramine*"[tiab] OR "Divonal"[tiab] OR "Dommanate"[tiab] OR "Dramaban"[tiab] OR "Dramamine"[tiab] OR "Dramanate"[tiab] OR "Dramarin"[tiab] OR "Dramasan"[tiab] OR "Dramyl"[tiab] OR "Drimen"[tiab] OR "Epha*"[tiab] OR "Faston"[tiab] OR "Gravamin"[tiab] OR "Gravol"[tiab] OR "Lomarin"[tiab] OR "Mareol"[tiab] OR "Marmine"[tiab] OR "Menhydrinate"[tiab] OR "Menito"[tiab] OR "Motion-Aid"[tiab] OR "Nausex"[tiab] OR "Nausicalm"[tiab] OR "Novomin"[tiab] OR "Paranausine"[tiab] OR "Pasedol"[tiab] OR "Reisegold"[tiab] OR "Reisetabletten*"[tiab] OR "Rodovan"[tiab] OR "Rubiemen"[tiab] OR "Superpep"[tiab] OR "Travel gum"[tiab] OR "Travelin"[tiab] OR "Travelmin"[tiab] OR "Travel Well"[tiab] OR "Trawell"[tiab] OR "Triptone"[tiab] OR "Valontan"[tiab] OR "Vomacur"[tiab] OR "Vomex*"[tiab] OR "Vomisin"[tiab] OR "Votmine"[tiab] OR "Wehamine"[tiab] OR "Xamamina"[tiab] OR "dexbrompheniramin*" [Supplementary Concept] OR "dexbrompheniramin*" [tiab] OR "Disomer*"[tiab] OR "Disophrol"[tiab] OR "desloratadine" [Supplementary Concept] OR "desloratadin*"[tiab] OR "descarboethoxyloratadin*"[tiab] OR "decarbethoxyloratadin*"[tiab] OR "Aerius"[tiab] OR "Aerogal"[tiab] OR "Alerdin"[tiab] OR "Alergoteva"[tiab] OR "Aleric deslo"[tiab] OR "Allex"[tiab] OR "Aviant"[tiab] OR "Azomyr"[tiab] OR "Claramax"[tiab] OR "Clarinex*"[tiab] OR "Clarus"[tiab] OR "Dasergin"[tiab] OR "Dasmini"[tiab] OR "Dasselta"[tiab] OR "Dasseltino"[tiab] OR "Dassergo"[tiab] OR "Delortan"[tiab] OR "Denosin"[tiab] OR "Desalergo"[tiab] OR "Desalex"[tiab] OR "Deslodyna"[tiab] OR "Deslor*"[tiab] OR "Deslox"[tiab] OR "Destamin"[tiab] OR "Dynid"[tiab] OR "Escontral"[tiab] OR "Flynise"[tiab] OR "Goldesin alerstop"[tiab] OR "Hitaxa"[tiab] OR "Jovesto"[tiab] OR "Kevenix"[tiab] OR "Lentrica"[tiab] OR "Loranopro"[tiab] OR "Lordestin"[tiab] OR "Lotera"[tiab] OR "Neoclaritine"[tiab] OR "Neoclarityn"[tiab] OR "Opulis"[tiab] OR "Sinalerg"[tiab] OR "Spirabel"[tiab] OR "Supraler"[tiab] OR "Teslor"[tiab] OR "Yosqiero"[tiab] OR "Diphenhydramine"[Mesh] OR "diphenhydrami*"[tiab] OR "diphenydrami*"[tiab] OR "diphenylhydramin*"[tiab] OR "diphendramin*"[tiab]OR "Alledryl"[tiab] OR "Allerdryl"[tiab] OR "Allergical"[tiab] OR "Allergina"[tiab] OR "Amidryl"[tiab] OR "Bagodryl"[tiab] OR "Banaril"[tiab] OR "Beldin"[tiab] OR "Belix"[tiab] OR "Benadril"[tiab] OR "Benadrin"[tiab] OR "Benadryl*"[tiab] OR "Benadyl"[tiab] OR "Benapon"[tiab] OR "Benocten"[tiab] OR "Benodin*"[tiab] OR "Benylan"[tiab] OR "Benzhydramine"[tiab] OR "Benhydramin"[tiab] OR "Ben-hydramin"[tiab] OR "Benadryl"[tiab] OR "Benylin"[tiab] OR "Broncho d"[tiab] OR "Caladryl"[tiab] OR "Carphenamine"[tiab] OR "Carphenex"[tiab] OR "Cathejell"[tiab] OR "Compoz"[tiab] OR "Dermistina"[tiab] OR "Desentol"[tiab] OR "Dibadorm n"[tiab] OR "Dibendrin"[tiab] OR "Dibenil"[tiab] OR "Dibondrin"[tiab] OR "Dibrondrin"[tiab] OR "difenhydramin*"[tiab] OR "Dihidral"[tiab] OR "Dimedrol"[tiab] OR "Dimedryl"[tiab] OR "Dimidril"[tiab] OR "Dimiril"[tiab] OR "Diphantine"[tiab] OR "Diphedryl"[tiab] OR "Diphen"[tiab] OR "Diphenacen"[tiab] OR "Dobacen"[tiab] OR "Dormin"[tiab] OR "Dryhistan"[tiab] OR "Dytan"[tiab] OR "emesan"[tiab] OR "histergan"[tiab] OR "hydramine"[tiab] OR "hyrexin"[tiab] OR "ibiodral"[tiab] OR "medidryl"[tiab] OR "ethylamine"[tiab] OR "neosynodorm"[tiab] OR "nytol quickgels"[tiab] OR "o benzhydryldimethylaminoethanol"[tiab] OR "pm 255"[tiab] OR "q-dryl"[tiab] OR "reisegold"[tiab] OR "resmin"[tiab] OR "restamin"[tiab] OR "sediat"[tiab] OR "sedryl"[tiab] OR "silphen"[tiab] OR "sleepeze"[tiab] OR "sominex"[tiab] OR "syntedril"[tiab] OR "trux-adryl"[tiab] OR "tzoali"[tiab] OR "unisom sleepgels"[tiab] OR "valdrene"[tiab] OR "valu-dryl"[tiab] OR "venasmin"[tiab] OR "vertirosan"[tiab] OR "vicks formula 44"[tiab] OR "vilbin"[tiab] OR "wehdryl"[tiab] OR "ziradryl"[tiab] OR "Doxylamine"[Mesh] OR "doxylamin*"[tiab] OR "brompheniramine maleate, pseudoephedrine sulfate drug combination" [Supplementary Concept] OR "Drixoral*"[tiab] OR "Dimethindene"[Mesh] OR "dimethinden*"[tiab] OR "dimetinden*"[tiab] OR "Dimethpyrindene"[tiab] OR "Fenistil"[tiab] OR "Forhistal"[tiab] OR "diphenylpyraline" [Supplementary Concept] OR "diphenylpyralin*"[tiab] OR "Lergobine"[tiab] OR "ebastine" [Supplementary Concept] OR "ebastin*"[tiab] OR "Busidril"[tiab] OR "Kestine"[tiab] OR "Evastel" [tiab] OR "Kestin" [tiab] OR "Bactil" [tiab] OR "Ebastel" [tiab] OR "fexofenadine" [Supplementary Concept] OR "fexofenadine*" [tiab] OR "Allegra" [tiab] OR "Telfast" [tiab] OR "Flunarizine"[Mesh] OR "flunarizin*"[tiab] OR "Sibelium"[tiab] OR "Hydroxyzine"[Mesh] OR "hydroxyzin*"[tiab] OR "Vistaril"[tiab] OR "Orgatrax"[tiab] OR "Durrax"[tiab] OR "Atarax"[tiab] OR "Ketotifen"[Mesh] OR "ketotifen"[tiab] OR "ketotiphen"[tiab] OR "Zaditen"[tiab] OR "levocetirizine" [Supplementary Concept] OR "levocetirizin*" [tiab] OR "Xusal" [tiab] OR "Xyzal" [tiab] OR "levocabastine" [Supplementary Concept] OR "levocabastin*" [tiab] OR "livostin" [tiab] OR "livocab" [tiab] OR "levophta" [tiab] OR "lévophta" [tiab] OR "Bilina" [tiab] OR "Loratadine"[Mesh] OR "loratadin*"[tiab] OR "Claritin"[tiab] OR "Clarityn"[tiab] OR "Alavert"[tiab] OR "Clarium*"[tiab] OR "Methapyrilene"[Mesh] OR "methapyrilen*"[tiab] OR "Thenylpyramine"[tiab] OR "Tenalin"[tiab] OR "Lullamin"[tiab] OR "Restryl"[tiab] OR "Thionylan"[tiab] OR "mequitazine" [Supplementary Concept] OR "mequitazin*" [tiab] OR "Primalan" [tiab] OR "Quitadrill" [tiab] OR "Mircol" [tiab] OR "Anti-Allergic Agents"[Mesh] OR "anti-allergic*"[tiab] OR "antiallergic*"[tiab] OR "anti-allergy*"[tiab] OR "antiallergy*"[tiab] OR "Promethazine"[Mesh] OR "promethazin*"[tiab] OR "prometazin*"[tiab] OR "Proazamine"[tiab] OR "Rumergan"[tiab] OR "Diprazin"[tiab] OR "Isopromethazine"[tiab] OR "Phenergan"[tiab] OR "Phenargan"[tiab] OR "Phensedyl"[tiab] OR "Pipolfen"[tiab] OR "Pipolphen"[tiab] OR "Promet"[tiab] OR "Prothazin"[tiab] OR "Pyrethia"[tiab] OR "Remsed"[tiab] OR "Atosil"[tiab] OR "Diphergan"[tiab] OR "Meclizine"[Mesh] OR "meclizin*"[tiab] OR "Parachloramine"[tiab] OR "Meclozine"[tiab] OR "Antivert"[tiab] OR "Bonamine"[tiab] OR "Bonine"[tiab] OR "Chiclida"[tiab] OR "Histametizyn"[tiab] OR "Ru-Vert-M"[tiab] OR "Agyrax"[tiab] OR "D-Vert"[tiab] OR "DVert"[tiab] OR "methdilazine" [Supplementary Concept] OR "methdilazin*"[tiab] OR "mizolastine" [Supplementary Concept] OR "mizolastin*"[tiab] OR "Mizollen"[tiab] OR "Zolim"[tiab] OR "Mizolen"[tiab] OR "Zolistan"[tiab] OR "Mistamine"[tiab] OR "Mistalin"[tiab] OR "Pyrilamine"[Mesh] OR "pyrilamin*"[tiab] OR "mepyramin*"[tiab] OR "Pyranisamine"[tiab] OR "Kriptin"[tiab] OR "Boots Bite and Sting Relief"[tiab] OR "Anthisan"[tiab] OR "oxatomide" [Supplementary Concept] OR "oxatomid*"[tiab] OR "Tinset"[tiab] OR "Pheniramine"[Mesh] OR "pheniramin*"[tiab] OR "propheniramin*"[tiab] OR "prophenpyridamine"[tiab] OR "Avil"[tiab] OR "Daneral"[tiab] OR "Histapyridamine"[tiab] OR "phenyltoloxamine" [Supplementary Concept] OR "phenyltoloxamin*"[tiab] OR "Tripelennamine"[Mesh] OR "tripelennamine"[tiab] OR "Vetibenzamin"[tiab] OR "Azaron"[tiab] OR "Pyribenzamine"[tiab] OR "Histantin"[tiab] OR "Triprolidine"[Mesh] OR "triprolidin*"[tiab] OR "Histex*"[tiab] OR "Pro Actidil"[tiab] OR "Actidil"[tiab] OR "tritoqualine" [Supplementary Concept] OR "tritoqualin*"[tiab] OR "Hypostamine"[tiab] OR "Inhibostamin"[tiab] OR "Dimotane*"[tiab] OR "Trimeprazine"[Mesh] OR "trimeprazin*"[tiab] OR "Methylpromazine"[tiab] OR "Alimemazine"[tiab] OR "Isobutrazine"[tiab] OR "Variargil"[tiab] OR "Spansule"[tiab] OR "Théralène"[tiab] OR "Repeltin"[tiab] OR "Nedeltran"[tiab] OR "Vallergan"[tiab] OR "Panectyl"[tiab] OR "carebastine" [Supplementary Concept] OR "carebastin*"[tiab] OR "alcaftadine" [Supplementary Concept] OR "alcaftadin*"[tiab] OR "Antazoline"[Mesh] OR "antazolin*"[tiab] OR "Phenazoline"[tiab] OR "Imidamine"[tiab] OR "Arithmin"[tiab] OR "Antasten"[tiab] OR "Analergine"[tiab] OR "Antistine"[tiab] OR "bamipine" [Supplementary Concept] OR "bamipin*"[tiab] OR "Soventol"[tiab] OR "benzonitrile" [Supplementary Concept] OR "benzonitril*"[tiab] OR "chloropyramine" [Supplementary Concept] OR "chloropyramine*"[tiab] OR "halopyramine"[tiab] OR "chlorpyramine"[tiab] OR "Suprastin"[tiab] OR "Avapena"[tiab] OR "dibenzheptropine" [Supplementary Concept] OR "dibenzheptropine*"[tiab] OR "deptropine*"[tiab] OR "Tropane"[tiab] OR "embramine" [Supplementary Concept] OR "embramine*"[tiab] OR "mebrophenhydramine"[tiab] OR "bromadryl"[tiab] OR "emedastine" [Supplementary Concept] OR "emedastine*"[tiab] OR "Emadine"[tiab] OR "fonazine" [Supplementary Concept] OR "fonazine*"[tiab] OR "dimethothiazine"[tiab] OR "dimetotiazine*"[tiab] OR "Promaquid"[tiab] OR "Migristene"[tiab] OR "Banistyl"[tiab] OR "histabudifen" [Supplementary Concept] OR "histabudifen"[tiab] OR "Mianserin"[Mesh] OR "mianserin*"[tiab] OR "Tolvon"[tiab] OR "Lerivon"[tiab] OR "Olopatadine Hydrochloride"[Mesh] OR "olopatadine*"[tiab] OR "Patanol"[tiab] OR "picumast" [Supplementary Concept] OR "picumast"[tiab] OR "protopine" [Supplementary Concept] OR "protopine*"[tiab] OR "fumarine"[tiab] OR "proxicromil" [Supplementary Concept] OR "proxicromil*"[tiab] OR "temelastine"[tiab] OR "tranilast" [Supplementary Concept] OR "tranilast*"[tiab] OR "Rizaben"[tiab] OR "trimeprazine tartrate" [Supplementary Concept] OR "trimeprazine tartrate"[tiab] OR "Theralen"[tiab] OR "Theraline"[tiab] OR "Temaril"[tiab] OR "Cetirizine"[Mesh] OR "cetirizin*"[tiab] OR “acidrine”[tiab] OR “actifed allergie”[tiab] OR “adezio”[tiab] OR “agelmin”[tiab] OR “alairgix*”[tiab] OR “alercet”[tiab] OR “alerid”[tiab] OR “alerlisin”[tiab] OR “alertop”[tiab] OR “alerviden”[tiab] OR “aletir”[tiab] OR “alled”[tiab] OR “allertec”[tiab] OR “aller-tec”[tiab] OR “alltec”[tiab] OR “alzytec”[tiab] OR “benaday”[tiab] OR “Benadryl*”[tiab] OR “betarhin”[tiab] OR “cabal”[tiab] OR “cerazine”[tiab] OR “cerini”[tiab] OR “cerotec”[tiab] OR “cesta”[tiab] OR “cetalerg”[tiab] OR “ceterifug”[tiab] OR “cethis”[tiab] OR “cetiderm”[tiab] OR “cetidura”[tiab] OR “cetilich”[tiab] OR “cetil von ct”[tiab] OR “cetimin”[tiab] OR “cetin”[tiab] OR “ceti-puren”[tiab] OR “cetirax”[tiab] OR “cetirigamma”[tiab] OR “cetirin”[tiab] OR “cetirlan”[tiab] OR “ceti TAD”[tiab] OR “cetizin”[tiab] OR “cetrimed”[tiab] OR “cetrine”[tiab] OR “cetrizet”[tiab] OR “cetrizin”[tiab] OR “cetymin”[tiab] OR “cistamine”[tiab] OR “deallergy”[tiab] OR “drill allergie”[tiab] OR “falergi”[tiab] OR “finallerg”[tiab] OR “formistin”[tiab] OR “generit”[tiab] OR “histazine”[tiab] OR “histica”[tiab] OR “incidal-od”[tiab] OR “lergium”[tiab] OR “livoreactine”[tiab] OR “nosemin”[tiab] OR “nosmin”[tiab] OR “ozen”[tiab] OR “piriteze allergy”[tiab] OR “pollenase”[tiab] OR “pollenshield hayfever relief”[tiab] OR “prixlae”[tiab] OR “quzyttir”[tiab] OR “qzytir”[tiab] OR “raingen”[tiab] OR “razene”[tiab] OR “reactine”[tiab] OR “rhizin”[tiab] OR “risima”[tiab] OR “ritecam”[tiab] OR “ryvel”[tiab] OR “ryzen”[tiab] OR “sancotec”[tiab] OR “selitex”[tiab] OR “setizin”[tiab] OR “simtec”[tiab] OR “sutac”[tiab] OR “symitec”[tiab] OR “terizin”[tiab] OR “terzine”[tiab] OR “vick-zyrt”[tiab] OR “virlix”[tiab] OR “zenriz”[tiab] OR “zensil”[tiab] OR “zeran”[tiab] OR “zertine”[tiab] OR “zerviate”[tiab] OR “zetir”[tiab] OR “zicet”[tiab] OR “zinex”[tiab] OR “ziralton”[tiab] OR “zirtec”[tiab] OR “zirtek”[tiab] OR “zirtin”[tiab] OR “zyllergy”[tiab] OR “zymed”[tiab] OR “zyrac”[tiab] OR “zyrazine”[tiab] OR “zyrcon”[tiab] OR “zyrlex”[tiab] OR “zyrtec*”[tiab] OR “zyrtek”[tiab])

Calcium channel blockers

("Calcium Channel Blockers"[Mesh] OR "calcium channel block*"[tiab] OR "calcium channel antagonist*"[tiab] OR "calcium channel inhibitor*"[tiab] OR "calcium inhibitor*"[tiab] OR "calcium antagonist*"[tiab] OR "calcium block*"[tiab] OR "calcium entry block*"[tiab] OR "Amlodipine"[Mesh] OR "amlodipin*"[tiab] OR "Amloc"[tiab] OR "Amlodis"[tiab] OR "Amlopin"[tiab] OR "Amlor"[tiab] OR "Astudal"[tiab] OR "Istin"[tiab] OR "Norvasc"[tiab] OR "Stamlo"[tiab] OR "Nifedipine"[Mesh] OR "nifedipine*"[tiab] OR "Adalat*"[tiab] OR "Adanif*"[tiab] OR "Adefin*"[tiab] OR "Adipine*"[tiab] OR "Afeditab*"[tiab] OR "Aldipin"[tiab] OR "Alonix-S"[tiab] OR "Alpha-nifedipine retard"[tiab] OR "Amarkor"[tiab] OR "Angibloc"[tiab] OR "Angipec"[tiab] OR "Antiblut"[tiab] OR "Apo-nifed"[tiab] OR "Aponifed"[tiab] OR "Aprical*"[tiab] OR "Atanaal softcap"[tiab] OR "Bay 1040"[tiab] OR "Bay1040"[tiab] OR "Bay a 1040"[tiab] OR "Bay a1040"[tiab] OR "Baya1040"[tiab] OR "Calcheck"[tiab] OR "Calcibloc*"[tiab] OR "Calcigard*"[tiab] OR "Calcilat"[tiab] OR "Calgina"[tiab] OR "Cardifen"[tiab] OR "Cardilat*"[tiab] OR "Cardionorm"[tiab] OR "Chronadalat*"[tiab] OR "Cipilat"[tiab] OR "Citilat"[tiab] OR "Coracten*"[tiab] OR "Cordafen"[tiab] OR "Cordaflex"[tiab] OR "Cordalat"[tiab] OR "Cordicant"[tiab] OR "Cordipen"[tiab] OR "Cordipin*"[tiab] OR "Corinfar"[tiab] OR "Korinfar"[tiab] OR "Coronpin"[tiab] OR "Corotrend"[tiab] OR "Denkifed"[tiab] OR "Depin"[tiab] OR "Dexipress*"[tiab] OR "Dignokonstant"[tiab] OR "Dilafed"[tiab] OR "Dipinkor"[tiab] OR "Duranifin"[tiab] OR "Ecodipin"[tiab] OR "Emaberin"[tiab] OR "Fedcor"[tiab] OR "Fedipin*"[tiab] OR "Fenamon*"[tiab] OR "Fenigidin"[tiab] OR "Fortipine"[tiab] OR "Glopir"[tiab] OR "Hadipine*"[tiab] OR "Herlat"[tiab] OR "Hexadilat"[tiab] OR "Hypan"[tiab] OR "Hypolar Retard"[tiab] OR "Infedipine"[tiab] OR "Jutadilat"[tiab] OR "Kemolat"[tiab] OR "Macorel"[tiab] OR "Megalat"[tiab] OR "Mifedipine"[tiab] OR "Moderat"[tiab] OR "Myogard"[tiab] OR "Nadipine"[tiab] OR "Nedipin"[tiab] OR "Nefedipine"[tiab] OR "Nelapine*"[tiab] OR "Neozipine*"[tiab] OR "Nidef"[tiab] OR "Nifangin"[tiab] OR "Nifar"[tiab] OR "Nifdemin"[tiab] OR "Nife-par"[tiab] OR "Nifebene"[tiab] OR "Nifecard"[tiab] OR "Nifecor"[tiab] OR "Nifedepat"[tiab] OR "Nifedicor*"[tiab] OR "Nifedilat"[tiab] OR "Nifedin*"[tiab] OR "Nifedipat"[tiab] OR "Nifedipin"[tiab] OR "Nifedipres*"[tiab] OR "Nifedirex lp"[tiab] OR "Nifehexal"[tiab] OR "Nifelat*"[tiab] OR "Nifensar*"[tiab] OR "Nifepidine"[tiab] OR "Nifestad"[tiab] OR "Nifical"[tiab] OR "Nificard"[tiab] OR "Nifidine"[tiab] OR "Nifipen"[tiab] OR "Nifopress*"[tiab] OR "Nimodrel*"[tiab] OR "Nipin*"[tiab] OR "Normadil"[tiab] OR "Novo nifedin"[tiab] OR "Novonifedin"[tiab] OR "Nyefax*"[tiab] OR "Nypine"[tiab] OR "Odipin"[tiab] OR "Orix"[tiab] OR "Osmo-adalat"[tiab] OR "Phenygidine"[tiab] OR "Pidilat*"[tiab] OR "Procardia*"[tiab] OR "Rdd 1219"[tiab] OR "Rdd1219"[tiab] OR "Ronian"[tiab] OR "Sepamit"[tiab] OR "Slofedipine*"[tiab] OR "Tensipine*"[tiab] OR "Tibricol"[tiab] OR "Unidipine*"[tiab] OR "Valni*"[tiab] OR "Vascard"[tiab] OR "Vasdalat*"[tiab] OR "Zenusin"[tiab] OR "clevidipine" [Supplementary Concept] OR "clevidipine*"[tiab] OR "Clevelox"[tiab] OR "Cleviprex"[tiab] OR "Nimodipine"[Mesh] OR "nimodipin*"[tiab] OR "Admon"[tiab] OR "Bay 9736"[tiab] OR "Bay736"[tiab]OR "Bay e9736"[tiab] OR "Bay e 9736"[tiab] OR "Bay n 5247"[tiab] OR "Bay n5247"[tiab] OR "Bay n 5248"[tiab] OR "Bay n5248"[tiab] OR "Brainal"[tiab] OR "Calnit"[tiab] OR "Eugerial"[tiab] OR "Grifonimod"[tiab] OR "Kenesil"[tiab] OR "Kenzolol"[tiab] OR "Modus"[tiab] OR "Nidip"[tiab] OR "Nimodilat"[tiab] OR "Nimotop"[tiab] OR "Nisom"[tiab] OR "Nymalize"[tiab] OR "Periplum"[tiab] OR "Remontal"[tiab] OR "Tropocer"[tiab] OR "Vasoflex"[tiab] OR "Vasotop"[tiab] OR "Nitrendipine"[Mesh] OR "Nitrendipin*"[tiab] OR "Balminil"[tiab] OR "Bay e 5009"[tiab] OR "Bay e5009"[tiab] OR "Baylotensin"[tiab] OR "Bayotensin*"[tiab] OR "Baypresol"[tiab] OR "Baypress*"[tiab] OR "Gericin"[tiab] OR "Jutapress"[tiab] OR "Lusopress"[tiab] OR "Nitre AbZ"[tiab] OR "Nidrel"[tiab] OR "Nitregamma"[tiab] OR "Nitren 1A Pharma"[tiab] OR "Nitren Lich"[tiab] OR "Nitre-Puren"[tiab] OR "NitrePuren"[tiab] OR "Niprina"[tiab] OR "Nitrend KSK"[tiab] OR "Nitrendepat"[tiab] OR "Nitren acis"[tiab] OR "Nitrendi Biochemie"[tiab] OR "Nitrendidoc"[tiab] OR "Nitrendimerck"[tiab] OR "Nitrendimerck"[tiab] OR "Nitrendipine"[tiab] OR "Nitrensal"[tiab] OR "Nitrepress"[tiab] OR "Trendinol"[tiab] OR "Tensogradal"[tiab] OR "Vastensium"[tiab] OR "Nicardipine"[Mesh] OR "nicardipine*"[tiab] OR "Angioglebil"[tiab] OR "Antagonil"[tiab] OR "Barizine"[tiab] OR "Bionicard"[tiab] OR "Cardene*"[tiab] OR "Cardepine*"[tiab] OR "Cardibloc"[tiab] OR "Cardipene"[tiab] OR "Dacarel"[tiab] OR "Dafil"[tiab] OR "Dagan"[tiab] OR "Flusemide"[tiab] OR "Karden"[tiab] OR "Lecibral"[tiab] OR "Lincil"[tiab] OR "Loxen"[tiab] OR "Lucenfal"[tiab] OR "Nerdipine"[tiab] OR "Nicaplant"[tiab] OR "Nicardal"[tiab] OR "Nicardil"[tiab] OR "Nicardipino*"[tiab] OR "Nicarpin"[tiab] OR "Nicodel"[tiab] OR "Nimicor"[tiab] OR "Perdipina"[tiab] OR "Perdipine*"[tiab] OR "Ranvil"[tiab] OR "Ridene"[tiab] OR "Roxen"[tiab] OR "rs 69216*"[tiab] OR "rs69216*"[tiab] OR "Rycarden"[tiab] OR "Rydene"[tiab] OR "Saf Card"[tiab] OR "SR Nicardipine"[tiab] OR "Vasodin"[tiab] OR "Vasonase"[tiab] OR "yc 93"[tiab] OR "yc93"[tiab] OR "y 93"[tiab] OR "y93"[tiab] OR "lercanidipine" [Supplementary Concept] OR "lercanidipine*" [tiab] OR "Atover"[tiab] OR "Brunoq"[tiab] OR "Carmen ace"[tiab] OR "Corifeo"[tiab] OR "Coripren"[tiab] OR "Enalapril*"[tiab] OR "Karnidin"[tiab] OR "Ledip"[tiab] OR "Lercadip"[tiab] OR "Lercan"[tiab] OR "Lercanidipine*"[tiab] OR "Lercaprel"[tiab] OR "Lercapress"[tiab] OR "Lercastad"[tiab] OR "Lercatio"[tiab] OR "Lercaton"[tiab] OR "Lerdip"[tiab] OR "Lerez"[tiab] OR "Lerka"[tiab] OR "Lerzam"[tiab] OR "Masnidipine"[tiab] OR "Primacor"[tiab] OR "Rec 15 2375"[tiab] OR "Rec 15-2375"[tiab] OR "Rec 152375"[tiab] OR "Rec15 2375"[tiab] OR "Rec15-2375"[tiab] OR "Rec152375"[tiab] OR "Tjn 324"[tiab] OR "Tjn324"[tiab] OR "Vasodip"[tiab] OR "Zanedip"[tiab] OR "Zan-extra"[tiab] OR "Zaneril"[tiab] OR "Zanextra"[tiab] OR "Zanipress"[tiab] OR "Zanidip"[tiab] OR "Zanipril"[tiab] OR "Zanitek"[tiab] OR "Felodipine"[Mesh] OR "felodipine"[tiab] OR "Agon*"[tiab] OR "Delofine*"[tiab] OR "Dilahex"[tiab] OR "Dilofen*"[tiab] OR "Dilopin"[tiab] OR "Fedil"[tiab] OR "Felim"[tiab] OR "Felo Biochemie"[tiab] OR "Felo ER"[tiab] OR "Felo-basf*"[tiab] OR "Felobal"[tiab] OR "Felobeta"[tiab] OR "Felocor*"[tiab] OR "Felodipin*"[tiab] OR "Felodur*"[tiab] OR "Felogamma*"[tiab] OR "Felogard"[tiab] OR "Felop"[tiab] OR "Felopine-sr"[tiab] OR "Felo-puren"[tiab] OR "Fensel"[tiab] OR "Flodil*"[tiab] OR "h 154-82"[tiab] OR "h 15482"[tiab] OR "h154-82"[tiab] OR "h15482"[tiab] OR "Hydac"[tiab] OR "Keydipin*"[tiab] OR "Lodistad*"[tiab] OR "Modip"[tiab] OR "Munobal*"[tiab] OR "Nirmadil"[tiab] OR "Parmid*"[tiab] OR "Penedil"[tiab] OR "Perfudal"[tiab] OR "Plendil*"[tiab] OR "Preslow"[tiab] OR "Prevex"[tiab] OR "Renedil"[tiab] OR "Selepine"[tiab] OR "Splendil*"[tiab] OR "Versant xr"[tiab] OR 'diltiazem'/exp OR "diltiazem"[tiab] OR "acalix"[tiab] OR "adizem*"[tiab] OR "aldizem"[tiab] OR "altiazem*"[tiab] OR "anginyl"[tiab] OR "angiodrox"[tiab] OR "angiotrofen"[tiab] OR "angiotrofin*"[tiab] OR "angiozem"[tiab] OR "angitil"[tiab] OR "angizem"[tiab] OR "angoral"[tiab] OR "anoheal"[tiab] OR "anotrit"[tiab] OR "anzem"[tiab] OR "apo-diltiazem"[tiab] OR "auscard"[tiab] OR "balcor"[tiab] OR "beatizem"[tiab] OR "bi-tildiem*"[tiab] OR "blocalcin"[tiab] OR "bnp 32762"[tiab] OR "bnp32762"[tiab] OR "britiazim"[tiab] OR "bruzem"[tiab] OR "calcicard"[tiab] OR "calnurs"[tiab] OR "cardcal"[tiab] OR "cardiazem"[tiab] OR "cardiben s.r."[tiab] OR "cardiem"[tiab] OR "cardil*"[tiab] OR "cardiosta*"[tiab] OR "cardium"[tiab] OR "Cardizem*"[tiab] OR "carex"[tiab] OR "carreldon*"[tiab] OR "cartia*"[tiab] OR "cascor*"[tiab] OR "cirilen*"[tiab] OR "cis diltiazem"[tiab] OR "coras"[tiab] OR "cordizem"[tiab] OR "crd 401"[tiab] OR "crd401"[tiab] OR "d2521"[tiab] OR "dazil"[tiab] OR "deltazen"[tiab] OR "diacor*"[tiab] OR "diatal"[tiab] OR "diazem*"[tiab] OR "dilacor*"[tiab] OR "diladel"[tiab] OR "dilatam*"[tiab] OR "dilcard*"[tiab] OR "dilem*"[tiab] OR "dilfar"[tiab] OR "dilgard"[tiab] OR "diloc"[tiab] OR "dilren*"[tiab] OR "dilso"[tiab] OR "dilt-cd"[tiab] OR "diltahexal"[tiab] OR "diltam"[tiab] OR "diltan*"[tiab] OR "diltelan"[tiab] OR "diltia*"[tiab] OR "diltime"[tiab] OR "diltiwas"[tiab] OR "diltstar"[tiab] OR "diltzac"[tiab] OR "dilzanton"[tiab] OR "dilzem*"[tiab] OR "dilzene"[tiab] OR "dilzereal*"[tiab] OR "dilzicardin"[tiab] OR "dinisor*"[tiab] OR "doclis*"[tiab] OR "dodexen*"[tiab] OR "dyalac"[tiab] OR "entrydil"[tiab] OR "etizem"[tiab] OR "filazem"[tiab] OR "gadoserin"[tiab] OR "grifodilzem"[tiab] OR "hagen"[tiab] OR "helsibon"[tiab] OR "herben"[tiab] OR "herbesser*"[tiab] OR "herbessor*"[tiab] OR "hesor"[tiab] OR "incoril*"[tiab] OR "iski-90"[tiab] OR "kaizem"[tiab] OR "kenzem"[tiab] OR "lacerol*"[tiab] OR "levodex"[tiab] OR "levozem"[tiab] OR "lytelsen"[tiab] OR "masdil*"[tiab] OR "miocardie"[tiab] OR "mk 793"[tiab] OR "mono-tildiem*"[tiab] OR "monotildiem"[tiab] OR "myonil*"[tiab] OR "pazeadin"[tiab] OR "presoken"[tiab] OR "slozem"[tiab] OR "slv 324"[tiab] OR "slv324"[tiab] OR "surazem"[tiab] OR "tazem"[tiab] OR "taztia*"[tiab] OR "tiadil"[tiab] OR "tiadylt"[tiab] OR "tiamate"[tiab] OR "tiazac"[tiab] OR "tilazem*"[tiab] OR "tildiem*"[tiab] OR "tilker"[tiab] OR "trans diltiazem"[tiab] OR "uni-masdil"[tiab] OR "vasmulax"[tiab] OR "vasocardol*"[tiab] OR "ven 307"[tiab] OR "ven307"[tiab] OR "wentizem*"[tiab] OR "zandil"[tiab] OR "zatim*"[tiab] OR "zemret*"[tiab] OR "zemtrial"[tiab] OR "zildem"[tiab] OR "ziruvate"[tiab] OR 'verapamil'/exp OR "verapamil"[tiab] OR "apo-verap"[tiab] OR "apoacor"[tiab] OR "arpamyl"[tiab] OR "azupamil"[tiab] OR "berkatens"[tiab] OR "calan*"[tiab] OR "calaptin*"[tiab] OR "cardiagutt"[tiab] OR "cardibeltin"[tiab] OR "cardiolen"[tiab] OR "cardiover"[tiab] OR "caveril"[tiab] OR "cintsu"[tiab] OR "civicor"[tiab] OR "coer 24"[tiab] OR "coraver"[tiab] OR "cordilat"[tiab] OR "cordilox*"[tiab] OR "corpamil"[tiab] OR "covera*"[tiab] OR "cp 16533 1"[tiab] OR "cp 16533-1"[tiab] OR "cp 165331"[tiab] OR "cp16533 1"[tiab] OR "cp16533-1"[tiab] OR "cp165331"[tiab] OR "d 365"[tiab] OR "d365"[tiab] OR "dignover"[tiab] OR "dilacoran*"[tiab] OR "dilacoron"[tiab] OR "durasoptin"[tiab] OR "falicard"[tiab] OR "finoptin"[tiab] OR "flamon"[tiab] OR "geangin"[tiab] OR "hexasoptin*"[tiab] OR "ikacor"[tiab] OR "ikakor"[tiab] OR "ikapress"[tiab] OR "iproveratril"[tiab] OR "iso-card*"[tiab] OR "isoptin*"[tiab] OR "levo verapamil"[tiab] OR "manidon*"[tiab] OR "napamil"[tiab] OR "novapamyl"[tiab] OR "novo-veramil"[tiab] OR "novopressan"[tiab] OR "phynoptin"[tiab] OR "quasar"[tiab] OR "ravamil*"[tiab] OR "securon*"[tiab] OR "univer"[tiab] OR "vasolan"[tiab] OR "vasomil"[tiab] OR "vasopten"[tiab] OR "verabeta"[tiab] OR "veracaps*"[tiab] OR "veracor"[tiab] OR "verahexal"[tiab] OR "veraloc"[tiab] OR "veramex*"[tiab] OR "veramil"[tiab] OR "verapamil*"[tiab] OR "verapin"[tiab] OR "verapress*"[tiab] OR "veratad"[tiab] OR "verdilac"[tiab] OR "verelan*"[tiab] OR "veroptin stada"[tiab] OR "verpamil"[tiab] OR "vetrimil"[tiab] OR "vortac"[tiab] OR "zolvera"[tiab])

Antimalarials

("Antimalarials"[Mesh] OR "antimalarial*"[tiab] OR "anti-malarial*"[tiab] OR "artemisinin" [Supplementary Concept] OR "artemisinin*"[tiab] OR "arteannuin"[tiab] OR "qinghaosu"[tiab] OR "quinghaosu"[tiab] OR "huanghuahaosu"[tiab] OR "qing hao su"[tiab] OR "quing hau sau"[tiab] OR "Sesquiterpenes"[Mesh:NoExp] OR "artemether"[tiab] OR "artesunate"[tiab] OR "Chloroquine"[Mesh] OR "chloroquine*"[tiab] OR "chlorochin"[tiab] OR "Chingamin"[tiab] OR "Khingamin"[tiab] OR "Nivaquine"[tiab] OR "Aralen"[tiab] OR "Arequin"[tiab] OR "Arechine"[tiab] OR "Hydroxychloroquine"[Mesh] OR "hydroxychloroquine*"[tiab] OR "hydroxycloroquine*"[tiab] OR "oxychloroquine*"[tiab] OR "oxycloroquine*"[tiab] OR "hydrochloroquine*"[tiab] OR "hydrocloroquine*"[tiab] OR "hydroxychlorochin"[tiab] OR "oxychlorochin"[tiab] OR "oxychloroquine"[tiab] OR "Plaquenil"[tiab] OR "chloroquinol"[tiab] OR "ercoquin"[tiab] OR "Pyrimethamine"[Mesh] OR "pyrimethamin*"[tiab] OR "pirimethamin*"[tiab] OR "primethamine"[tiab] OR "Malocide"[tiab] OR "Tindurine"[tiab] OR "Chloridin*"[tiab] OR "Daraprim"[tiab] OR "Darapram"[tiab] OR "Darapraim"[tiab] OR "Daraprin"[tiab] OR "Malocide"[tiab] OR "tindurin"[tiab] OR "Proguanil"[Mesh] OR "Balusil"[tiab] OR "Bigumal"[tiab] OR "proguanil*"[tiab] OR "Chloriguane"[tiab] OR "Chlorguanid*"[tiab] OR "Chlorguanil"[tiab] OR "Chloroguanid*"[tiab] OR "Cloroguanid*"[tiab] OR "Chloriguane"[tiab] OR "Diguanil"[tiab] OR "Diguanyl"[tiab] OR "Drinupal"[tiab] OR "Guanatol"[tiab] OR "Paguinal*"[tiab] OR "Paludrin*"[tiab] OR "Palusil"[tiab] OR "Plasin"[tiab] OR "Proguanide"[tiab] OR "Proquanil"[tiab] OR "Tirian"[tiab] OR "Sulfadoxine"[Mesh] OR "sulfadoxin*"[tiab] OR "Fanasil"[tiab] OR "Fanzil"[tiab] OR "Sanasil"[tiab] OR "sulfaorthodimethoxin*"[tiab] OR "sulfonmetoxin*"[tiab] OR "sulfonmethoxin*"[tiab] OR "sulformetoxin*"[tiab] OR "sulformethoxin*"[tiab] OR "sulfon metoxin*"[tiab] OR "sulfon methoxin*"[tiab] OR "sulfor metoxin*"[tiab] OR "sulfor methoxin*"[tiab] OR "sulforthomidin*"[tiab] OR "sulforthodimethoxin*"[tiab] OR "sulfurmetoxin*"[tiab] OR "sulphadoxin*"[tiab] OR "sulphormethoxin*"[tiab] OR "sulphor methoxin*"[tiab] OR "sulphormetoxin*"[tiab] OR "sulphor metoxin*"[tiab] OR "Dapsone"[Mesh] OR "dapson*"[tiab] OR "Aczone"[tiab] OR "Atrisone"[tiab] OR "Avlosulfan"[tiab] OR "Avlosulfon*"[tiab] OR "Croysulfone*"[tiab] OR "Dapsoderm-X"[tiab] OR "DADPS"[tiab] OR "DDS"[tiab] OR "diamino diphenyl sulfone"[tiab] OR "diaminodiphenyl sulfone"[tiab] OR "diaminodiphenylsulfon*"[tiab] OR "diammodiphenylsulfon*"[tiab] OR "diaphenylsulfon*"[tiab] OR "diaphenyl sulfon*"[tiab] OR "diaphenylsulphon*"[tiab] OR "diaphenyl sulphon*"[tiab] OR "Disulone"[tiab] OR "Diphenason*"[tiab] OR "Diphone"[tiab] OR "Dopsan"[tiab] OR "Dumitone"[tiab] OR "Eporal"[tiab] OR "Lepravir"[tiab] OR "Novasulfon"[tiab] OR "Novophone"[tiab] OR "sulfonyldianiline"[tiab] OR "Servidapson*"[tiab] OR "Sulfadione"[tiab] OR "Sulfadoine"[tiab] OR "Sulfona*"[tiab] OR "Sulfone"[tiab] OR "Udolac"[tiab])

Other medications

*Antibiotics*

("Anti-Bacterial Agents"[Mesh] OR "anti-infective*"[tiab] OR "antiinfective*"[tiab] OR "anti-bacterial*"[tiab] OR "antibacterial*"[tiab] OR "anti-mycobacterial*"[tiab] OR "antimycobacterial*"[tiab] OR "bacteriocid*"[tiab] OR "antibiotic*"[tiab] OR "antimicrobial*"[tiab] OR "antiseptic*"[tiab] OR "chemotherapeutic agent"[tiab] OR "chemotherapeutic drug"[tiab] OR "chemotherapeutica"[tiab] OR "microbiological agent"[tiab] OR "Cephalexin"[Mesh] OR "cephalexin*"[tiab] OR "cefalexin"[tiab] OR "Ceporexine"[tiab] OR "Palitrex"[tiab] OR "Cefixime"[Mesh] OR "cefixime*"[tiab] OR "Suprax"[tiab] OR "Cefuroxime"[Mesh] OR "cefuroxime"[tiab] OR "cephuroxime"[tiab] OR "Zinacef"[tiab] OR "Ketocef"[tiab] OR "Ciprofloxacin"[Mesh] OR "cipro*"[tiab] OR "Ciprinol"[tiab] OR "fleroxacin"[tiab] OR "Quinodis"[tiab] OR "Erythromycin"[Mesh] OR "erythromycin*"[tiab] OR "T-Stat"[tiab] OR "TStat"[tiab] OR "Erymax"[tiab] OR "Erycette"[tiab] OR "Ilotycin"[tiab] OR "Gemifloxacin"[Mesh] OR "gemifloxacin*"[tiab] OR "Factive"[tiab] OR "Levofloxacin"[Mesh] OR "levofloxacin*"[tiab] OR "Quixin"[tiab] OR "Levaquin"[tiab] OR "Metronidazole"[Mesh] OR "metronidazole*"[tiab] OR "Satric"[tiab] OR "Trichazol"[tiab] OR "Trichopol"[tiab] OR "Trivazol"[tiab] OR "Vagilen"[tiab] OR "Bayer 5360"[tiab] OR "Danizol"[tiab] OR "Flagyl"[tiab] OR "Gineflavir"[tiab] OR "Metric"[tiab] OR "Metrodzhil"[tiab] OR "MetroGel"[tiab] OR "Metrogyl"[tiab] OR "Clont"[tiab] OR "Spiramycin"[Mesh] OR "spiramycin*"[tiab] OR "Rovamycine"[tiab] OR "Selectomycin"[tiab] OR "Rovamycin"[tiab])

*Antivirals*

("Antiviral Agents"[Mesh] OR "antiviral*"[tiab] OR "anti viral*"[tiab] OR "antivirus*"[tiab] OR "virucidal*"[tiab] OR "viral inhibitor*"[tiab] OR "virus repressor*"[tiab] OR "virostatic*"[tiab] OR "virustatic*"[tiab] OR "virucide*"[tiab] OR "Foscarnet"[Mesh] OR "foscarnet*"[tiab] OR "phosphonoform*"[tiab] OR "Foscavir*"[tiab])

*Anesthetics*

("Anesthetics"[Mesh] OR "anesthetic*"[tiab] OR "anaesthetic*"[tiab] OR "preanaesthetic*"[tiab] OR "preanesthetic*"[tiab] OR "Nitric Oxide"[Mesh] OR "nitric oxide"[tiab] OR "nitrogen monoxide"[tiab] OR "mononitrogen monoxide"[tiab] OR "genosyl"[tiab] OR "inomax"[tiab] OR "noxivent"[tiab] OR "Fentanyl"[Mesh] OR "fentanyl*"[tiab] OR "phentanyl*"[tiab] OR “defent”[tiab] OR “duragesic*”[tiab] OR “durogesic”[tiab] OR “durotep”[tiab] OR “epufen”[tiab] OR “fentalis”[tiab] OR “fentamat”[tiab] OR “fentamyl”[tiab] OR “fentanest”[tiab] OR “fentanex”[tiab] OR “fentanil”[tiab] OR “fentora”[tiab] OR “fetanex”[tiab] OR “fetnanyl”[tiab] OR “leptanal”[tiab] OR “matrifen”[tiab] OR “mezolar matrix”[tiab] OR “pecfent”[tiab] OR “rapinyl”[tiab] OR “recuvyra”[tiab] OR “sublimaze”[tiab] OR “subsys”[tiab] OR “tanyl”[tiab] OR “tilotrans”[tiab] OR “transfenta”[tiab] OR “wafernyl”[tiab] OR "Morphine"[Mesh] OR "morphine*"[tiab] OR “MS Contin”[tiab] OR “Oramorph SR”[tiab] OR “Duramorph”[tiab] OR “anpec”[tiab] OR “duromorph”[tiab] OR “epimorph”[tiab] OR “miro”[tiab] OR “morfin”[tiab] OR “morfine”[tiab] OR “morphia”[tiab] OR “morphin”[tiab] OR “morphinium”[tiab] OR “morphium”[tiab] OR “opso”[tiab] OR “skenan”[tiab] OR "Propofol"[Mesh] OR "propofol*"[tiab] OR “anepol”[tiab] OR “anesia”[tiab] OR “aquafol”[tiab] OR “cryotol”[tiab] OR “diisoprofol”[tiab] OR “diprivan”[tiab] OR “diprofol”[tiab] OR “disoprivan”[tiab] OR “disoprofol”[tiab] OR “fresofol”[tiab] OR “gobbifol”[tiab] OR “hiremon”[tiab] OR “ivofol”[tiab] OR “plofed”[tiab] OR “pofol”[tiab] OR “profast”[tiab] OR “propocam”[tiab] OR “propolipid”[tiab] OR “propoven”[tiab] OR “provive”[tiab] OR “rapinovet”[tiab] OR “rapiva”[tiab] OR “recofol*”[tiab] OR “ripol”[tiab] OR “safol”[tiab] OR “spifol”[tiab] OR “spiva”[tiab] OR “unifol “[tiab] OR "Sevoflurane"[Mesh] OR "sevofluran*"[tiab] OR “petrem”[tiab] OR “sevo-anesteran”[tiab] OR “sevocalm”[tiab] OR “sevoflo”[tiab] OR “sevofrane”[tiab] OR “sevohale”[tiab] OR “sevorane”[tiab] OR “sevotec”[tiab] OR “sojourn”[tiab] OR “ultane*”[tiab])

*Analgesics & antipyretics*

("Analgesics"[Mesh] OR "analgesic*"[tiab] OR "analgetic*"[tiab] OR "antalgic*"[tiab] OR "anodyne*"[tiab] OR "Antinociceptive*"[tiab] OR "Antipyretics"[Mesh] OR "antipyretic*"[tiab] OR "antifebrile*"[tiab] OR "Acetaminophen"[Mesh] OR "acetaminophen*"[tiab] OR "hydroxyacetanilide"[tiab] OR "paracetamol"[tiab] OR "Acetamidophenol"[tiab] OR "Acephen"[tiab] OR "Acetaco"[tiab] OR "Tylenol"[tiab] OR "Anacin*"[tiab] OR "Datril"[tiab] OR "Panadol"[tiab] OR "Acamol"[tiab] OR "Algotropyl"[tiab] OR "Pentazocine"[Mesh] OR "pentazocin*"[tiab] OR "pentozocin*"[tiab] OR "pentacozin*"[tiab] OR "Lexir"[tiab] OR “dolapent”[tiab] OR “fortal”[tiab] OR “fortalgesic”[tiab] OR “fortral*”[tiab] OR “fortwin”[tiab] OR “liticon”[tiab] OR “peltazon”[tiab] OR “pentafen”[tiab] OR “pentagin”[tiab] OR “pentalgina”[tiab] OR “perutagin”[tiab] OR “sosegon”[tiab] OR “sosigon”[tiab] OR “talioin”[tiab] OR “talwin”[tiab])

*Antitussives*

("Antitussive Agents"[Mesh] OR "antitussive*"[tiab] OR "cough suppressant*"[tiab] OR "cough depressant*"[tiab] OR "anticough*"[tiab] OR "butamirate" [Supplementary Concept] OR "butamirate*" [tiab] OR "butamyrate*" [tiab] OR "Sinecod"[tiab] OR "cloperastine" [Supplementary Concept] OR "cloperastine*" [tiab] OR "Codeine"[Mesh] OR "codein*"[tiab] OR "Ardinex"[tiab] OR "Tussicalm"[tiab] OR "Pentuss"[tiab] OR "Dextromethorphan"[Mesh] OR "dextromethorphan*"[tiab] OR "methorphan*"[tiab] OR "racemethorphan*"[tiab] OR "Delsym"[tiab])

*BZD*

("Bromazepam"[Mesh] OR "bromazepam*"[tiab] OR “bromazepam”[tiab] OR “anyrex”[tiab] OR “bartul”[tiab] OR “bromalich”[tiab] OR “bromaz 1A pharma”[tiab] OR “bromazanil”[tiab] OR “bromazep Von Ct”[tiab] OR “compedium”[tiab] OR “creosedi”[tiab] OR “durazanil”[tiab] OR “lectopam”[tiab] OR “lekotam”[tiab] OR “lexamil”[tiab] OR “lexatin”[tiab] OR “lexaurin”[tiab] OR “lexilium”[tiab] OR “lexomil”[tiab] OR “lexotan*”[tiab] OR “normoc”[tiab] OR “sintrogel”[tiab] OR "brotizolam" [Supplementary Concept] OR "brotizolam"[tiab] OR “dormex”[tiab] OR “ladormin”[tiab] OR “lendorm”[tiab] OR “lendormin”[tiab] OR “lindormin”[tiab] OR “mederantil”[tiab] OR “noctilan”[tiab] OR “sintonal”[tiab] OR "Zolpidem"[Mesh] OR "zolpidem*"[tiab] OR "Zolirin"[tiab] OR "Zolpi-Lich"[tiab] OR "Zolpinox"[tiab] OR "Zolpimist"[tiab] OR "Ambien"[tiab] OR "Amsic"[tiab] OR "Bikalm"[tiab] OR "Stilnoct"[tiab] OR "Stilnox"[tiab] OR "Dalparan"[tiab] OR "Zodormdura"[tiab] OR "Zoldem"[tiab])

*Retinoids*

("Retinoids"[Mesh] OR "retinoid*"[tiab] OR "Isotretinoin"[Mesh] OR "isotretinoin"[tiab] OR “a cnotren”[tiab] OR “a-cnotren”[tiab] OR “abosorica”[tiab] OR “absorica*”[tiab] OR “accuran”[tiab] OR “accure”[tiab] OR “accutane*”[tiab] OR “accutin”[tiab] OR “acnal sc”[tiab] OR “acnemin”[tiab] OR “acnenor”[tiab] OR “acnetrait”[tiab] OR “acnetrex”[tiab] OR “acnogen”[tiab] OR “acnotin”[tiab] OR “acnotren”[tiab] OR “actaven”[tiab] OR “aisoskin”[tiab] OR “akinol”[tiab] OR “aknenormin”[tiab] OR “amnesteem”[tiab] OR “axotret”[tiab] OR “ciscumed”[tiab] OR “ciscutan”[tiab] OR “claravis”[tiab] OR “contracne”[tiab] OR “curacne*”[tiab] OR “curatane”[tiab] OR “decutan”[tiab] OR “dercutane”[tiab] OR “flexresan”[tiab] OR “inerta”[tiab] OR “isdiben”[tiab] OR “iso tretinoin”[tiab] OR “isoacne”[tiab] OR “isocural”[tiab] OR “isoderm”[tiab] OR “isogalen”[tiab] OR “isomacne”[tiab] OR “isoretinoic acid”[tiab] OR “isoretinoin”[tiab] OR “isoriac”[tiab] OR “isosupra lidose”[tiab] OR “isotane”[tiab] OR “isotren”[tiab] OR “isotret”[tiab] OR “isotret-hexal”[tiab] OR “isotrex*”[tiab] OR “isotroin”[tiab] OR “izotek”[tiab] OR “izotziaja”[tiab] OR “mayesta”[tiab] OR “medinac”[tiab] OR “myorisan”[tiab] OR “newtinon sc”[tiab] OR “nimegen”[tiab] OR “oratane”[tiab] OR “pharmiso”[tiab] OR “pinple”[tiab] OR “procuta”[tiab] OR “procuta ge”[tiab] OR “reducar”[tiab] OR “reticutan”[tiab] OR “retinoin, iso”[tiab] OR “rizuderm”[tiab] OR “roaccutan*”[tiab] OR “roaccuttan”[tiab] OR “roacnetan”[tiab] OR “roacutan”[tiab] OR “roacuttan”[tiab] OR “rocne”[tiab] OR “rotaxin”[tiab] OR “rotren”[tiab] OR “sotret”[tiab] OR “stiefotrex”[tiab] OR “teriosal”[tiab] OR “tretin”[tiab] OR “tretinex”[tiab] OR “tretiosan”[tiab] OR “tretoskin”[tiab] OR “zenatane”[tiab])

*Anthelminthics*

("Anthelmintics"[Mesh] OR "anthelmintic*"[tiab] OR "anthelminthic*"[tiab] OR "antihelmintic*"[tiab] OR "antihelminthic*"[tiab] OR "anti-helmintic*"[tiab] OR "anti-helminthic*"[tiab] OR "helminthicidal*"[tiab] OR "vermicid*"[tiab] OR "vermifuge*"[tiab] OR "Albendazole"[Mesh] OR "albendazol*"[tiab] OR “abentel”[tiab] OR “adazol”[tiab] OR “albatel”[tiab] OR “alben”[tiab] OR “albendazol”[tiab] OR “albendol”[tiab] OR “albendoral”[tiab] OR “albenza”[tiab] OR “albenzol”[tiab] OR “albex”[tiab] OR “albezole”[tiab] OR “alfuca”[tiab] OR “alminth”[tiab] OR “alzental”[tiab] OR “alzol”[tiab] OR “anthelmex”[tiab] OR “bendapar”[tiab] OR “bendex-400”[tiab] OR “bilutac”[tiab] OR “borotel”[tiab] OR “ceprazol”[tiab] OR “champs d-worm 6”[tiab] OR “ciclopar”[tiab] OR “digezanol”[tiab] OR “disthelm”[tiab] OR “endoplus”[tiab] OR “emanthal”[tiab] OR “eskazole”[tiab] OR “fintel”[tiab] OR “gascop”[tiab] OR “getzol”[tiab] OR “helben”[tiab] OR “helmiben”[tiab] OR “helmindazol”[tiab] OR “kalbend”[tiab] OR “labenda”[tiab] OR “loveral”[tiab] OR “lurdex”[tiab] OR “mebenix”[tiab] OR “metiazol”[tiab] OR “mycotel”[tiab] OR “nemozole”[tiab] OR “pantex”[tiab] OR “paranthil”[tiab] OR “proftril*”[tiab] OR “rotopar”[tiab] OR “rozolex”[tiab] OR “sioban”[tiab] OR “valbantel”[tiab] OR “valbazen”[tiab] OR “valbenzen”[tiab] OR “vastus”[tiab] OR “vemizol”[tiab] OR “vermin plus”[tiab] OR “xadem”[tiab] OR “zeben”[tiab] OR “zela”[tiab] OR “zentab”[tiab] OR “zentel”[tiab])

*Cytostatics*

("Cytostatic Agents"[Mesh] OR "cytostatic*"[tiab] OR "Capecitabine"[Mesh] OR "capecitabine"[tiab] OR “apecitab”[tiab] OR “atubri”[tiab] OR “cacit”[tiab] OR “capcel”[tiab] OR “capebina”[tiab] OR “capecel”[tiab] OR “capecitalox”[tiab] OR “capecite”[tiab] OR “capegard”[tiab] OR “capezam”[tiab] OR “capibine”[tiab] OR “capicet”[tiab] OR “capiibine”[tiab] OR “capnat”[tiab] OR “capoda”[tiab] OR “capostat”[tiab] OR “capsy”[tiab] OR “capxcel”[tiab] OR “caxeta”[tiab] OR “cerex”[tiab] OR “citabin”[tiab] OR “coloxet”[tiab] OR “ecansya”[tiab] OR “kapetral”[tiab] OR “naprocap”[tiab] OR “preveloda”[tiab] OR “xabine”[tiab] OR “xalvobin”[tiab] OR “xecap”[tiab] OR “xelazor”[tiab] OR “xelcip”[tiab] OR “xelocel”[tiab] OR “xeloda”[tiab] OR “zerectum”[tiab] OR “zocitab”[tiab])

*Cholinesterase inhibitors*

("Galantamine"[Mesh] OR "galantamin*"[tiab] OR "galanthamin*"[tiab] OR "acumor*”[tiab] OR "alenzo”[tiab] OR "aneprosil”[tiab] OR "bergal*”[tiab] OR "consion*”[tiab] OR "elmino*”[tiab] OR "gaalin”[tiab] OR "galanaxiro”[tiab] OR "galantex”[tiab] OR "galanthen”[tiab] OR "galanyl”[tiab] OR "galatamin*”[tiab] OR "galema*”[tiab] OR "galnora”[tiab] OR "galsya*”[tiab] OR "galzemic*”[tiab] OR "gamyl”[tiab] OR "gatalin*”[tiab] OR "gazylan*”[tiab] OR "girlamen”[tiab] OR "jilkon”[tiab] OR "lotprosin*”[tiab] OR "loxifren”[tiab] OR "luventa*”[tiab] OR "lycoremin*”[tiab] OR "margal”[tiab] OR "masparen*”[tiab] OR "memo (drug) “[tiab] OR "memoton life”[tiab] OR "memoton-life”[tiab] OR "micol (galantamine)“[tiab] OR "natagal*”[tiab] OR "nivalin*”[tiab] OR "razadyne*”[tiab] OR "reminyl*”[tiab] OR "spegal*”[tiab] OR "tamilin”[tiab] OR "vertusal*”[tiab] OR "zentan”[tiab] OR "zoroflog”[tiab] OR "Rivastigmine"[Mesh] OR "rivastigmin*"[tiab] OR "alzest"[tiab] OR "exelon*"[tiab] OR "kingsmin"[tiab] OR "nimvastid"[tiab] OR "prometax"[tiab] OR "rivastach"[tiab] OR "Donepezil"[Mesh] OR "donepezil*"[tiab] OR "adlarity”[tiab] OR “adonep”[tiab] OR “adtreat”[tiab] OR “alertan”[tiab] OR “alzancer”[tiab] OR “alzepezil”[tiab] OR “alzepil”[tiab] OR “alzil”[tiab] OR “apo-doperil”[tiab] OR “aricept*”[tiab] OR “aripezil”[tiab] OR “aripil”[tiab] OR “asenta”[tiab] OR “blixie”[tiab] OR “caricia”[tiab] OR “cenipil”[tiab] OR “ciclodin”[tiab] OR “cogiton*”[tiab] OR “covolos”[tiab] OR “darezil”[tiab] OR “darizol”[tiab] OR “davia”[tiab] OR “demelan”[tiab] OR “dementis”[tiab] OR “demezilon”[tiab] OR “destezil”[tiab] OR “dezial”[tiab] OR “donebrain”[tiab] OR “donecept”[tiab] OR “donectil*”[tiab] OR “donefien”[tiab] OR “donegal”[tiab] OR “doneliquid geriasan”[tiab] OR “donepesan”[tiab] OR “donepestan”[tiab] OR “donepex”[tiab] OR “doneprion”[tiab] OR “donerion”[tiab] OR “donestad”[tiab] OR “donester”[tiab] OR “donesyn”[tiab] OR “donpethon”[tiab] OR “dospelin”[tiab] OR “dozept”[tiab] OR “dozilax”[tiab] OR “eranz”[tiab] OR “filosept”[tiab] OR “hania”[tiab] OR “kognezil”[tiab] OR “landex”[tiab] OR “lixben”[tiab] OR “lizidra”[tiab] OR “medozil”[tiab] OR “memac”[tiab] OR “memorion”[tiab] OR “memorit”[tiab] OR “navazil”[tiab] OR “nepanizil”[tiab] OR “neperion”[tiab] OR “nepezil”[tiab] OR “nepla”[tiab] OR “oromentis”[tiab] OR “palixid”[tiab] OR “pamigen”[tiab] OR “penezil”[tiab] OR “pezale”[tiab] OR “peziled”[tiab] OR “pezimax”[tiab] OR “promemore”[tiab] OR “rafazil”[tiab] OR “ricordo”[tiab] OR “sulbenin”[tiab] OR “tuadin”[tiab] OR “uxazen”[tiab] OR “venaxen”[tiab] OR “yasnal”[tiab] OR “yasnoro”[tiab] OR “zakalmer”[tiab] OR “zauton”[tiab] OR “zinocept”[tiab] OR “zopitel”[tiab])

**EMBASE**

Dystonia

('dystonia'/exp OR 'dystonia*':ti,ab,kw OR 'myodystonia':ti,ab,kw OR 'myodystony':ti,ab,kw OR 'torticollis*':ti,ab,kw OR 'retrocollis*':ti,ab,kw OR 'anterocollis*':ti,ab,kw OR 'laterocollis*':ti,ab,kw OR 'wryneck':ti,ab,kw OR 'wry neck':ti,ab,kw OR 'loxia':ti,ab,kw OR 'muscle tonus*':ti,ab,kw OR 'motor dysfunction*':ti,ab,kw OR 'torsion spasm*':ti,ab,kw OR 'Meige*':ti,ab,kw OR 'Brueghel*':ti,ab,kw OR 'blepharospasm*':ti,ab,kw OR 'spasmodic dysphonia*':ti,ab,kw)

Antiemetics and gastrointestinal drugs

('antiemetic agent'/exp OR 'antiemetic*':ti,ab,kw OR 'anti-emetic*':ti,ab,kw OR 'anti emetic*':ti,ab,kw OR 'serotonin 3 antagonist'/exp OR 'serotonin 3 antagonist*':ti,ab,kw OR '5-HT3 receptor antagonist*':ti,ab,kw OR '5-HT3 receptor block*':ti,ab,kw OR '5-HT3 antagonist*':ti,ab,kw OR '5-HT 3 antagonist*':ti,ab,kw OR '5-HT3 block*':ti,ab,kw OR 'dopamine 2 receptor blocking agent'/exp OR 'dopamine 2 receptor blocking agent*':ti,ab,kw OR 'dopamine D2 receptor antagonist*':ti,ab,kw OR 'dopamine 2 receptor antagonist*':ti,ab,kw OR 'dopamine D2 receptor blocker*':ti,ab,kw OR 'dopamine 2 receptor block*':ti,ab,kw OR 'dopamine D2 antagonist*':ti,ab,kw OR 'dopamine 2 antagonist*':ti,ab,kw OR 'dopamine D2 block*':ti,ab,kw OR 'neurokinin 1 receptor antagonist'/exp OR 'neurokinin 1 receptor antagonist*':ti,ab,kw OR 'neurokinin 1 antagonist*':ti,ab,kw OR 'NK1 antagonist*':ti,ab,kw OR 'neurokinin-1 receptor block*':ti,ab,kw OR 'tachykinin receptor 1 antagonist*':ti,ab,kw OR 'tachykinin receptor 1 blocker*':ti,ab,kw OR 'substance P receptor antagonist*':ti,ab,kw OR 'substance P receptor blocker*':ti,ab,kw OR 'corticosteroid'/exp OR 'adrenal cortex hormone*':ti,ab,kw OR 'adrenal cortical hormone*':ti,ab,kw OR 'adrenocortical hormone*':ti,ab,kw OR 'adrenocortical steroid*':ti,ab,kw OR 'adrenocorticosteroid*':ti,ab,kw OR 'cortical steroid*':ti,ab,kw OR 'adreno cortical steroid*':ti,ab,kw OR 'adrenal cortical steroid*':ti,ab,kw OR 'adreno corticosteroid*':ti,ab,kw OR 'adrenal steroid*':ti,ab,kw OR 'corticosteroid*':ti,ab,kw OR 'cortico steroid*':ti,ab,kw OR 'corticoid*':ti,ab,kw OR 'histamine H1 receptor antagonist'/exp OR 'H1 receptor antagonist*':ti,ab,kw OR 'Histamine 1 receptor antagonist*':ti,ab,kw OR 'Histamine 1 receptor block*':ti,ab,kw OR 'H1 antihistamine*':ti,ab,kw OR 'H1 receptor block*':ti,ab,kw OR 'H1 antagonist*':ti,ab,kw OR 'H1 block*':ti,ab,kw OR 'H1 receptor blockader*':ti,ab,kw OR 'cholinergic receptor blocking agent'/exp OR 'cholinergic receptor antagonist*':ti,ab,kw OR 'cholinergic receptor block*':ti,ab,kw OR 'cholinergic antagonist*':ti,ab,kw OR 'cholinergic block*':ti,ab,kw OR 'cholinergic blocking agent*':ti,ab,kw OR 'cholinolytic*':ti,ab,kw OR 'acetylcholine antagonist*':ti,ab,kw OR 'acetylcholine receptor block*':ti,ab,kw OR 'acetylcholine receptor inhibitor*':ti,ab,kw OR 'AChR inhibitor*':ti,ab,kw OR 'acetylcholine block*':ti,ab,kw OR 'acetylcholine blocking agent*':ti,ab,kw OR 'anticholinergic*':ti,ab,kw OR 'anti-cholinergic*':ti,ab,kw OR 'parasympathetic agent*':ti,ab,kw OR 'parasympathetic drug*':ti,ab,kw OR 'parasympathetic block*':ti,ab,kw OR 'parasympatholytic agent*':ti,ab,kw OR 'parasympatholytic drug*':ti,ab,kw OR 'parasympatholytic block*':ti,ab,kw OR 'parasympatholytic*':ti,ab,kw OR 'parasympathicolytic agent*':ti,ab,kw OR 'parasympathicolytic drug*':ti,ab,kw OR 'parasympathicolytic block*':ti,ab,kw OR 'cholinolytic agent*':ti,ab,kw OR 'atropinic agent*':ti,ab,kw OR 'atropinic drug*':ti,ab,kw OR 'ondansetron'/exp OR 'ondansetron*':ti,ab,kw OR 'ad 04':ti,ab,kw OR 'ad04':ti,ab,kw OR 'avessaron':ti,ab,kw OR 'bryterol':ti,ab,kw OR 'cedantron':ti,ab,kw OR 'cellondan*':ti,ab,kw OR 'ceramos':ti,ab,kw OR 'emeset':ti,ab,kw OR 'gr 38032':ti,ab,kw OR 'gr 38032f':ti,ab,kw OR 'gr c507 75':ti,ab,kw OR 'gr38032':ti,ab,kw OR 'gr38032f':ti,ab,kw OR 'modifical':ti,ab,kw OR 'narfoz':ti,ab,kw OR 'onsia':ti,ab,kw OR 'sakisozin':ti,ab,kw OR 'setofilm':ti,ab,kw OR 'sn 307':ti,ab,kw OR 'sud 002':ti,ab,kw OR 'sud002':ti,ab,kw OR 'suda 002':ti,ab,kw OR 'suda002':ti,ab,kw OR 'vomceran':ti,ab,kw OR 'zensana':ti,ab,kw OR 'zetron':ti,ab,kw OR 'zofrene':ti,ab,kw OR 'zofron':ti,ab,kw OR 'zondamist':ti,ab,kw OR 'zofran*':ti,ab,kw OR 'zophran':ti,ab,kw OR 'zophren':ti,ab,kw OR 'zuplenz':ti,ab,kw OR 'granisetron'/exp OR 'granisetron':ti,ab,kw OR 'apf 530':ti,ab,kw OR 'apf530':ti,ab,kw OR 'axigran':ti,ab,kw OR 'az 010':ti,ab,kw OR 'az010':ti,ab,kw OR 'brl 43694':ti,ab,kw OR 'brl 43694a':ti,ab,kw OR 'brl43694':ti,ab,kw OR 'brl43694a':ti,ab,kw OR 'cam 2047':ti,ab,kw OR 'cam2047':ti,ab,kw OR 'emegar':ti,ab,kw OR 'eutrom':ti,ab,kw OR 'granegis':ti,ab,kw OR 'grani-denk':ti,ab,kw OR 'granicip':ti,ab,kw OR 'granigen':ti,ab,kw OR 'granisol':ti,ab,kw OR 'granissetrom':ti,ab,kw OR 'granitron':ti,ab,kw OR 'granizetron':ti,ab,kw OR 'kevatril':ti,ab,kw OR 'kytril':ti,ab,kw OR 'pandiol':ti,ab,kw OR 'rasetron':ti,ab,kw OR 'sancuso':ti,ab,kw OR 'sp 01':ti,ab,kw OR 'sp01':ti,ab,kw OR 'sustol':ti,ab,kw OR 'taraz':ti,ab,kw OR 'viatrinil':ti,ab,kw OR 'dolasetron mesilate'/exp OR 'dolasetron*':ti,ab,kw OR 'Anemet':ti,ab,kw OR 'Anzemet':ti,ab,kw OR 'mdl 73147':ti,ab,kw OR 'mdl 73147ef':ti,ab,kw OR 'mdl73147':ti,ab,kw OR 'mdl73147ef':ti,ab,kw OR 'zamanon':ti,ab,kw OR 'palonosetron'/exp OR 'palonosetron*':ti,ab,kw OR 'Aloxi':ti,ab,kw OR 'nuowei':ti,ab,kw OR 'onicit':ti,ab,kw OR 'paloxi':ti,ab,kw OR 'rs 25259':ti,ab,kw OR 'rs 25259 197':ti,ab,kw OR 'rs 25259-197':ti,ab,kw OR 'rs25259':ti,ab,kw OR 'rs25259 197':ti,ab,kw OR 'rs25259-197':ti,ab,kw OR 'ruo shan':ti,ab,kw OR 'tropisetron'/exp OR 'tropisetron*':ti,ab,kw OR 'endoprol':ti,ab,kw OR 'endostem':ti,ab,kw OR 'ics 205 930':ti,ab,kw OR 'ics 205, 930':ti,ab,kw OR 'ics 205-930':ti,ab,kw OR 'ics 205930':ti,ab,kw OR 'ics-205930':ti,ab,kw OR 'Navoban':ti,ab,kw OR 'novaban':ti,ab,kw OR 'novoban':ti,ab,kw OR 'metoclopramide'/exp OR 'metoclopramide':ti,ab,kw OR ‘ahr 3070 c’:ti,ab,kw OR ‘ahr 3070c’:ti,ab,kw OR ‘ahr3070c’:ti,ab,kw OR ‘ametic’:ti,ab,kw OR ‘anausin’:ti,ab,kw OR ‘apo-metoclop’:ti,ab,kw OR ‘aputern’:ti,ab,kw OR ‘betaclopramide’:ti,ab,kw OR ‘bondigest’:ti,ab,kw OR ‘carnotprim primperan’:ti,ab,kw OR ‘cerucal’:ti,ab,kw OR ‘clodilion’:ti,ab,kw OR ‘clopamon’:ti,ab,kw OR ‘clopan’:ti,ab,kw OR ‘clopra’:ti,ab,kw OR ‘clopra-yellow’:ti,ab,kw OR ‘clopram’:ti,ab,kw OR ‘degan’:ti,ab,kw OR ‘del 1267’:ti,ab,kw OR ‘del1267’:ti,ab,kw OR ‘dibertil’:ti,ab,kw OR ‘duraclamid’:ti,ab,kw OR ‘emenil’:ti,ab,kw OR ‘emetal’:ti,ab,kw OR ‘emetard’:ti,ab,kw OR ‘emitasol’:ti,ab,kw OR ‘emperal’:ti,ab,kw OR ‘encil’:ti,ab,kw OR ‘enzimar’:ti,ab,kw OR ‘gastro timelets’:ti,ab,kw OR ‘gastrobi*’:ti,ab,kw OR ‘gastronerton’:ti,ab,kw OR ‘gastrosil’:ti,ab,kw OR ‘gastrotem’:ti,ab,kw OR ‘gastrotimelets’:ti,ab,kw OR ‘gavistal’:ti,ab,kw OR ‘gensil’:ti,ab,kw OR ‘gimoli’:ti,ab,kw OR ‘gimoti’:ti,ab,kw OR ‘hemesis’:ti,ab,kw OR ‘hyrin’:ti,ab,kw OR ‘imperan’:ti,ab,kw OR ‘m 813’:ti,ab,kw OR ‘m813’:ti,ab,kw OR ‘maalox nausea’:ti,ab,kw OR ‘maril’:ti,ab,kw OR ‘maxeran’:ti,ab,kw OR ‘maxeron’:ti,ab,kw OR ‘maxolan’:ti,ab,kw OR ‘maxolon’:ti,ab,kw OR ‘mcp-beta tropfen’:ti,ab,kw OR ‘meclomid’:ti,ab,kw OR ‘meclopamide’:ti,ab,kw OR ‘meclopramide’:ti,ab,kw OR ‘meclopran’:ti,ab,kw OR ‘megaldrate’:ti,ab,kw OR ‘meramide’:ti,ab,kw OR ‘metaclopramide’:ti,ab,kw OR ‘metadrate’:ti,ab,kw OR ‘metagliz’:ti,ab,kw OR ‘metamide’:ti,ab,kw OR ‘methochlopramide’:ti,ab,kw OR ‘methoclopramide’:ti,ab,kw OR ‘methoclopramine’:ti,ab,kw OR ‘metlazel’:ti,ab,kw OR ‘metochlopramide’:ti,ab,kw OR ‘metoclopamide’:ti,ab,kw OR ‘metoclopramine’:ti,ab,kw OR ‘metoclopranide hydrochloride’:ti,ab,kw OR ‘metoclor’:ti,ab,kw OR ‘metoclorpramide’:ti,ab,kw OR ‘metocobil’:ti,ab,kw OR ‘metocyl’:ti,ab,kw OR ‘metodopramide’:ti,ab,kw OR ‘metolon’:ti,ab,kw OR ‘metopram’:ti,ab,kw OR ‘metox’:ti,ab,kw OR ‘metozolv*’:ti,ab,kw OR ‘metpamid’:ti,ab,kw OR ‘metram’:ti,ab,kw OR ‘moriperan’:ti,ab,kw OR ‘mygdalon’:ti,ab,kw OR ‘nausil’:ti,ab,kw OR ‘neopramiel’:ti,ab,kw OR ‘netaf’:ti,ab,kw OR ‘neu sensamide’:ti,ab,kw OR ‘nic 001’:ti,ab,kw OR ‘nic001’:ti,ab,kw OR ‘nilatika’:ti,ab,kw OR ‘normastin’:ti,ab,kw OR ‘octamide’:ti,ab,kw OR ‘opram’:ti,ab,kw OR ‘paspertin*’:ti,ab,kw OR ‘perinorm’:ti,ab,kw OR ‘pharmyork’:ti,ab,kw OR ‘plasil’:ti,ab,kw OR ‘pramidin’:ti,ab,kw OR ‘pramin’:ti,ab,kw OR ‘pramotel’:ti,ab,kw OR ‘primperan’:ti,ab,kw OR ‘primperil’:ti,ab,kw OR ‘prinparl’:ti,ab,kw OR ‘prokinyl lp’:ti,ab,kw OR ‘prowel’:ti,ab,kw OR ‘pulin’:ti,ab,kw OR ‘reclomide’:ti,ab,kw OR ‘reglan*’:ti,ab,kw OR ‘reliveran’:ti,ab,kw OR ‘rimetin’:ti,ab,kw OR ‘sensamide’:ti,ab,kw OR ‘sotatic-10’:ti,ab,kw OR ‘terperan’:ti,ab,kw OR ‘tomid’:ti,ab,kw OR ‘vertivom’:ti,ab,kw OR ‘vomitrol’:ti,ab,kw OR ‘zumatrol’:ti,ab,kw OR 'prochlorperazine'/exp OR 'prochlorperazine':ti,ab,kw OR ‘6140 rp’:ti,ab,kw OR ‘antinaus’:ti,ab,kw OR ‘az 001’:ti,ab,kw OR ‘az001’:ti,ab,kw OR ‘bayer a 173’:ti,ab,kw OR ‘bayer a173’:ti,ab,kw OR ‘capazine’:ti,ab,kw OR ‘chlormeprazine’:ti,ab,kw OR ‘chlorpeazine’:ti,ab,kw OR ‘chlorperazine’:ti,ab,kw OR ‘compro’:ti,ab,kw OR ‘dicopal’:ti,ab,kw OR ‘emelent’:ti,ab,kw OR ‘klometil’:ti,ab,kw OR ‘kronocin’:ti,ab,kw OR ‘meterazine’:ti,ab,kw OR ‘metherazine’:ti,ab,kw OR ‘nautisol’:ti,ab,kw OR ‘nipodal’:ti,ab,kw OR ‘normalmin’:ti,ab,kw OR ‘pasotomin’:ti,ab,kw OR ‘prochlor’:ti,ab,kw OR ‘prochlor perazine’:ti,ab,kw OR ‘prochlorpemazine’:ti,ab,kw OR ‘prochlorperacine’:ti,ab,kw OR ‘prochlorperzine’:ti,ab,kw OR ‘prochlorpromazine’:ti,ab,kw OR ‘proclorperazine’:ti,ab,kw OR ‘rp 6140’:ti,ab,kw OR ‘rp6140’:ti,ab,kw OR ‘sk and f 4657’:ti,ab,kw OR ‘skf 4657’:ti,ab,kw OR ‘skf4657’:ti,ab,kw OR ‘tementil’:ti,ab,kw OR ‘temetil’:ti,ab,kw OR 'droperidol'/exp OR 'droperidol':ti,ab,kw OR ‘dehidrobenzoperidol’:ti,ab,kw OR ‘dehydrobenzoperidol’:ti,ab,kw OR ‘dehydrobenzoperiol’:ti,ab,kw OR ‘dehydrobenzperidol’:ti,ab,kw OR ‘dehydrobenzperidolum’:ti,ab,kw OR ‘dridol’:ti,ab,kw OR ‘droleptan’:ti,ab,kw OR ‘droperol’:ti,ab,kw OR ‘halkan’:ti,ab,kw OR ‘inaprine’:ti,ab,kw OR ‘inapsin*’:ti,ab,kw OR ‘mcn jr 4749’:ti,ab,kw OR ‘mcn r 4749’:ti,ab,kw OR ‘oridol’:ti,ab,kw OR ‘r 4749’:ti,ab,kw OR ‘sintodian’:ti,ab,kw OR ‘troperidol’:ti,ab,kw OR ‘xomolix’:ti,ab,kw OR 'aprepitant'/exp OR 'aprepitant':ti,ab,kw OR ‘aponvie’:ti,ab,kw OR ‘aprepilor’:ti,ab,kw OR ‘atanto’:ti,ab,kw OR ‘cinvanti’:ti,ab,kw OR ‘emend’:ti,ab,kw OR ‘ht 001’:ti,ab,kw OR ‘ht001’:ti,ab,kw OR ‘htx 019’:ti,ab,kw OR ‘htx019’:ti,ab,kw OR ‘inepitant’:ti,ab,kw OR ‘l 754030’:ti,ab,kw OR ‘l754030’:ti,ab,kw OR ‘lorpitan’:ti,ab,kw OR ‘mk 0869’:ti,ab,kw OR ‘mk 869’:ti,ab,kw OR ‘mk0869’:ti,ab,kw OR ‘mk869’:ti,ab,kw OR ‘ono 7436’:ti,ab,kw OR ‘ono7436’:ti,ab,kw OR ‘tiperant’:ti,ab,kw OR ‘weg 232’:ti,ab,kw OR ‘weg232’:ti,ab,kw OR 'rolapitant'/exp OR 'rolapitant*':ti,ab,kw OR ‘sch 619734’:ti,ab,kw OR ‘sch619734’:ti,ab,kw OR ‘varubi’:ti,ab,kw OR ‘varuby’:ti,ab,kw OR 'dexamethasone'/exp OR 'dexamethason*':ti,ab,kw OR ‘adrecort’:ti,ab,kw OR ‘adrenocot’:ti,ab,kw OR ‘aeroseb*’:ti,ab,kw OR ‘aflucoson*’:ti,ab,kw OR ‘alfalyl’:ti,ab,kw OR ‘anaflogistico*’:ti,ab,kw OR ‘aphtasolon’:ti,ab,kw OR ‘arcodexan*’:ti,ab,kw OR ‘artrosone’:ti,ab,kw OR ‘auxiron’:ti,ab,kw OR ‘azium’:ti,ab,kw OR ‘baycadron’:ti,ab,kw OR ‘bidexol’:ti,ab,kw OR ‘bisu ds’:ti,ab,kw OR ‘calonat’:ti,ab,kw OR ‘cebedex’:ti,ab,kw OR ‘cetadexon’:ti,ab,kw OR ‘colofoam’:ti,ab,kw OR ‘corsona’:ti,ab,kw OR ‘corsone’:ti,ab,kw OR ‘cortastat*’:ti,ab,kw OR ‘cortidex’:ti,ab,kw OR ‘cortidexason’:ti,ab,kw OR ‘cortidrona’:ti,ab,kw OR ‘cortidrone’:ti,ab,kw OR ‘cortisumman’:ti,ab,kw OR ‘dacortina fuerte’:ti,ab,kw OR ‘dacortine fuerte’:ti,ab,kw OR ‘dalalone*’:ti,ab,kw OR ‘danasone’:ti,ab,kw OR ‘de-sone la’:ti,ab,kw OR ‘decacortin’:ti,ab,kw OR ‘decadeltoson*’:ti,ab,kw OR ‘decaderm’:ti,ab,kw OR ‘decadion’:ti,ab,kw OR ‘decadran’:ti,ab,kw OR ‘decadron*’:ti,ab,kw OR ‘decaesadril’:ti,ab,kw OR ‘decagel’:ti,ab,kw OR ‘decaject*’:ti,ab,kw OR ‘decalix’:ti,ab,kw OR ‘decameth*’:ti,ab,kw OR ‘decasone’:ti,ab,kw OR ‘decaspray’:ti,ab,kw OR ‘decasterolone’:ti,ab,kw OR ‘decdan’:ti,ab,kw OR ‘decilone*’:ti,ab,kw OR ‘decofluor’:ti,ab,kw OR ‘dectancyl’:ti,ab,kw OR ‘dekacort’:ti,ab,kw OR ‘delladec’:ti,ab,kw OR ‘deltafluoren*’:ti,ab,kw OR ‘dergramin’:ti,ab,kw OR ‘deronil’:ti,ab,kw OR ‘desacort*’:ti,ab,kw OR ‘desadrene’:ti,ab,kw OR ‘desalark’:ti,ab,kw OR ‘desameton*’:ti,ab,kw OR ‘desigdron’:ti,ab,kw OR ‘dexa cortisyl’:ti,ab,kw OR ‘dexa dabrosan’:ti,ab,kw OR ‘dexa korti’:ti,ab,kw OR ‘dexa scherosan’:ti,ab,kw OR ‘dexa scherozon*’:ti,ab,kw OR ‘dexa-p’:ti,ab,kw OR ‘dexacen 4’:ti,ab,kw OR ‘dexachel’:ti,ab,kw OR ‘dexacollyre’:ti,ab,kw OR ‘dexacort*’:ti,ab,kw OR ‘dexadabroson’:ti,ab,kw OR ‘dexadecadrol’:ti,ab,kw OR ‘dexadrol’:ti,ab,kw OR ‘dexafarma’:ti,ab,kw OR ‘dexagel’:ti,ab,kw OR ‘dexagen’:ti,ab,kw OR ‘dexahelvacort’:ti,ab,kw OR ‘dexakorti’:ti,ab,kw OR ‘dexalien’:ti,ab,kw OR ‘dexaliquid geriasan’:ti,ab,kw OR ‘dexalocal’:ti,ab,kw OR ‘dexame’:ti,ab,kw OR ‘dexamecortin’:ti,ab,kw OR ‘dexameson*’:ti,ab,kw OR ‘dexametason*’:ti,ab,kw OR ‘dexameth*’:ti,ab,kw OR ‘dexamonozon’:ti,ab,kw OR ‘dexan’:ti,ab,kw OR ‘dexane’:ti,ab,kw OR ‘dexano’:ti,ab,kw OR ‘dexapolcort’:ti,ab,kw OR ‘dexapot’:ti,ab,kw OR ‘dexaschero*’:ti,ab,kw OR ‘dexason*’:ti,ab,kw OR ‘dexinoral’:ti,ab,kw OR ‘dexionil’:ti,ab,kw OR ‘dexmethsone’:ti,ab,kw OR ‘dexona’:ti,ab,kw OR ‘dexone*’:ti,ab,kw OR ‘dexpak*’:ti,ab,kw OR ‘dextelan’:ti,ab,kw OR ‘dextenza’:ti,ab,kw OR ‘dextrasone’:ti,ab,kw OR ‘dexycu*’:ti,ab,kw OR ‘dezone’:ti,ab,kw OR ‘dibasona’:ti,ab,kw OR ‘doxamethasone’:ti,ab,kw OR ‘esacortene’:ti,ab,kw OR ‘ex s1’:ti,ab,kw OR ‘exadion*’:ti,ab,kw OR ‘firmalone’:ti,ab,kw OR ‘fluormethyl*’:ti,ab,kw OR ‘fluormone’:ti,ab,kw OR ‘fluorocort’:ti,ab,kw OR ‘fluorodelta’:ti,ab,kw OR ‘fluoromethylprednisolone’:ti,ab,kw OR ‘fortecortin’:ti,ab,kw OR ‘gammacorten*’:ti,ab,kw OR ‘glensoludex’:ti,ab,kw OR ‘grosodexon*’:ti,ab,kw OR ‘hemady’:ti,ab,kw OR ‘hexadecadiol’:ti,ab,kw OR ‘hexadecadrol’:ti,ab,kw OR ‘hexadiol’:ti,ab,kw OR ‘hexadrol’:ti,ab,kw OR ‘isicort’:ti,ab,kw OR ‘isnacort’:ti,ab,kw OR ‘isopto*’:ti,ab,kw OR ‘isv 305’:ti,ab,kw OR ‘isv305’:ti,ab,kw OR ‘lokalison f’:ti,ab,kw OR ‘loverine’:ti,ab,kw OR ‘luxazone’:ti,ab,kw OR ‘marvidione’:ti,ab,kw OR ‘maxidex’:ti,ab,kw OR ‘mediamethasone’:ti,ab,kw OR ‘megacortin’:ti,ab,kw OR ‘mephameson*’:ti,ab,kw OR ‘metasolon*’:ti,ab,kw OR ‘methazon*’:ti,ab,kw OR ‘methylfluorprednisolone’:ti,ab,kw OR ‘metisone lafi’:ti,ab,kw OR ‘mexasone’:ti,ab,kw OR ‘millicorten*’:ti,ab,kw OR ‘mk 125’:ti,ab,kw OR ‘mk125’:ti,ab,kw OR ‘mymethasone’:ti,ab,kw OR ‘neoforderx’:ti,ab,kw OR ‘neofordex’:ti,ab,kw OR ‘nisomethasona’:ti,ab,kw OR ‘nodexon’:ti,ab,kw OR ‘novocort’:ti,ab,kw OR ‘nsc 34521’:ti,ab,kw OR ‘nsc34521’:ti,ab,kw OR ‘oftan-dexa’:ti,ab,kw OR ‘opticorten’:ti,ab,kw OR ‘opticortinol’:ti,ab,kw OR ‘oradexan’:ti,ab,kw OR ‘oradexon*’:ti,ab,kw OR ‘orgadrone’:ti,ab,kw OR ‘oto 104’:ti,ab,kw OR ‘oto104’:ti,ab,kw OR ‘ozurdex’:ti,ab,kw OR ‘perazone’:ti,ab,kw OR ‘pidexon’:ti,ab,kw OR ‘policort’:ti,ab,kw OR ‘posurdex’:ti,ab,kw OR ‘predni-f*’:ti,ab,kw OR ‘prednisolone f’:ti,ab,kw OR ‘prodexona’:ti,ab,kw OR ‘prodexone’:ti,ab,kw OR ‘sanamethasone’:ti,ab,kw OR ‘santenson’:ti,ab,kw OR ‘santeson’:ti,ab,kw OR ‘sawasone’:ti,ab,kw OR ‘sk 0503’:ti,ab,kw OR ‘sk0503’:ti,ab,kw OR ‘solurex*’:ti,ab,kw OR ‘spoloven’:ti,ab,kw OR ‘spt 2101’:ti,ab,kw OR ‘spt2101’:ti,ab,kw OR ‘sterasone’:ti,ab,kw OR ‘thilodexine’:ti,ab,kw OR ‘triamcimetil’:ti,ab,kw OR ‘vexamet’:ti,ab,kw OR ‘visumetazone’:ti,ab,kw OR ‘visumethazone’:ti,ab,kw OR ‘zeqmelit’:ti,ab,kw OR 'scopolamine'/exp OR 'scopolamine':ti,ab,kw OR ‘atrochin’:ti,ab,kw OR ‘atroquin’:ti,ab,kw OR ‘atroscine’:ti,ab,kw OR ‘Boro-Scopol’:ti,ab,kw OR ‘dl atroscine’:ti,ab,kw OR ‘hyosceine’:ti,ab,kw OR ‘hyoscine*’:ti,ab,kw OR ‘hysco’:ti,ab,kw OR ‘kimite-patch’:ti,ab,kw OR ‘Kwells’:ti,ab,kw OR ‘l epoxytropine tropate’:ti,ab,kw OR ‘l hyoscine’:ti,ab,kw OR ‘n methylhyoscine’:ti,ab,kw OR ‘oscine’:ti,ab,kw OR ‘Scoburen’:ti,ab,kw OR ‘Scopace’:ti,ab,kw OR ‘scopalamine*’:ti,ab,kw OR ‘scopine tropate’:ti,ab,kw OR ‘Scopoderm*’:ti,ab,kw OR ‘transcop’:ti,ab,kw OR ‘Transderm*’:ti,ab,kw OR ‘Travacalm*’:ti,ab,kw OR ‘tropic acid ester with scopine’:ti,ab,kw OR 'ranitidine'/exp OR 'ranitidin*':ti,ab,kw OR 'acidex':ti,ab,kw OR 'aciloc':ti,ab,kw OR 'acloral':ti,ab,kw OR 'acran':ti,ab,kw OR 'aldin':ti,ab,kw OR 'alquen':ti,ab,kw OR 'anistal':ti,ab,kw OR 'antagonin':ti,ab,kw OR 'ardoral':ti,ab,kw OR 'atural':ti,ab,kw OR 'ausran':ti,ab,kw OR 'avintac':ti,ab,kw OR 'axoban':ti,ab,kw OR 'azantac':ti,ab,kw OR 'baroxal':ti,ab,kw OR 'biotidin':ti,ab,kw OR 'consec':ti,ab,kw OR 'coralen':ti,ab,kw OR 'cygran':ti,ab,kw OR 'duractin':ti,ab,kw OR 'eltidine':ti,ab,kw OR 'eu-ran':ti,ab,kw OR 'ezopta':ti,ab,kw OR 'galidrin':ti,ab,kw OR 'gastran':ti,ab,kw OR 'gastrial':ti,ab,kw OR 'gastridina':ti,ab,kw OR 'gastrosedol':ti,ab,kw OR 'hexer':ti,ab,kw OR 'histac':ti,ab,kw OR 'histak':ti,ab,kw OR 'hyzan':ti,ab,kw OR 'incid':ti,ab,kw OR 'iqfadina':ti,ab,kw OR 'kemoranin':ti,ab,kw OR 'kiradin':ti,ab,kw OR 'lumaren':ti,ab,kw OR 'lydin':ti,ab,kw OR 'microtid':ti,ab,kw OR 'midaven':ti,ab,kw OR 'nadine':ti,ab,kw OR 'neoceptin-r':ti,ab,kw OR 'novo-ranidine':ti,ab,kw OR 'pilorex':ti,ab,kw OR 'ponaltin':ti,ab,kw OR 'ptinolin':ti,ab,kw OR 'quantor':ti,ab,kw OR 'quicran':ti,ab,kw OR 'r-loc':ti,ab,kw OR 'radinat':ti,ab,kw OR 'radine':ti,ab,kw OR 'rafitaz':ti,ab,kw OR 'ranacid':ti,ab,kw OR 'rancet':ti,ab,kw OR 'randin':ti,ab,kw OR 'rani 2':ti,ab,kw OR 'ranial':ti,ab,kw OR 'raniben':ti,ab,kw OR 'ranibloc':ti,ab,kw OR 'ranicalm':ti,ab,kw OR 'ranidil':ti,ab,kw OR 'ranidine':ti,ab,kw OR 'ranidura':ti,ab,kw OR 'ranigast*':ti,ab,kw OR 'ranihexal':ti,ab,kw OR 'ranimex':ti,ab,kw OR 'ranin':ti,ab,kw OR 'raniogas':ti,ab,kw OR 'raniplex':ti,ab,kw OR 'ranisan':ti,ab,kw OR 'ranisen':ti,ab,kw OR 'ranitab':ti,ab,kw OR 'ranital':ti,ab,kw OR 'ranitax':ti,ab,kw OR 'ranitil':ti,ab,kw OR 'ranitin*':ti,ab,kw OR 'ranolta':ti,ab,kw OR 'rantac':ti,ab,kw OR 'rantacid':ti,ab,kw OR 'rantin':ti,ab,kw OR 'ranuber':ti,ab,kw OR 'ranzac':ti,ab,kw OR 'ratic':ti,ab,kw OR 'raticina':ti,ab,kw OR 'raxide':ti,ab,kw OR 'retamin':ti,ab,kw OR 'rolan':ti,ab,kw OR 'rosimol':ti,ab,kw OR 'simetac':ti,ab,kw OR 'sostril':ti,ab,kw OR 'tanidina':ti,ab,kw OR 'taural':ti,ab,kw OR 'terodul':ti,ab,kw OR 'toriol':ti,ab,kw OR 'ulcaid':ti,ab,kw OR 'ulceran':ti,ab,kw OR 'ulceranin':ti,ab,kw OR 'ulcex':ti,ab,kw OR 'ulcin':ti,ab,kw OR 'ulcocur':ti,ab,kw OR 'ulsal':ti,ab,kw OR 'ultak':ti,ab,kw OR 'ultidine':ti,ab,kw OR 'urantac':ti,ab,kw OR 'verlost':ti,ab,kw OR 'vesyca':ti,ab,kw OR 'vizerul':ti,ab,kw OR 'weichilin':ti,ab,kw OR 'weidos':ti,ab,kw OR 'xanidine':ti,ab,kw OR 'zantab':ti,ab,kw OR 'zantac*':ti,ab,kw OR 'zantadin':ti,ab,kw OR 'zantic':ti,ab,kw OR 'zinetac':ti,ab,kw OR 'cisapride'/exp OR 'cisapride':ti,ab,kw OR 'acenalin':ti,ab,kw OR 'acpulsif':ti,ab,kw OR 'alimix*':ti,ab,kw OR 'alipride':ti,ab,kw OR 'cipride':ti,ab,kw OR 'cisamod':ti,ab,kw OR 'cisaprid*':ti,ab,kw OR 'cisapron':ti,ab,kw OR 'cisawal':ti,ab,kw OR 'disflux':ti,ab,kw OR 'dizmoprida':ti,ab,kw OR 'enteropride':ti,ab,kw OR 'eriken':ti,ab,kw OR 'esorid':ti,ab,kw OR 'ethiprid':ti,ab,kw OR 'gastromet':ti,ab,kw OR 'guarposid':ti,ab,kw OR 'kineprid':ti,ab,kw OR 'kinestase':ti,ab,kw OR 'metison':ti,ab,kw OR 'motilar':ti,ab,kw OR 'palcid':ti,ab,kw OR 'prepulsid':ti,ab,kw OR 'presiston':ti,ab,kw OR 'prider':ti,ab,kw OR 'pridesia':ti,ab,kw OR 'prisic':ti,ab,kw OR 'propulsid*':ti,ab,kw OR 'propulsin':ti,ab,kw OR 'propulsit':ti,ab,kw OR 'pulsar':ti,ab,kw OR 'refluxin':ti,ab,kw OR 'risamol':ti,ab,kw OR 'saprid':ti,ab,kw OR 'stimulit':ti,ab,kw OR 'syspride':ti,ab,kw OR 'tono-cis':ti,ab,kw OR 'unamol':ti,ab,kw OR 'unipride':ti,ab,kw OR 'viprasen':ti,ab,kw OR 'vomipride':ti,ab,kw OR 'wepride':ti,ab,kw OR 'cimetidine'/exp OR 'cimetidine':ti,ab,kw OR 'aci-med':ti,ab,kw OR 'acibilin':ti,ab,kw OR 'acidnor':ti,ab,kw OR 'acinil':ti,ab,kw OR 'aidar':ti,ab,kw OR 'altramet':ti,ab,kw OR 'antag':ti,ab,kw OR 'apo-cimetidine':ti,ab,kw OR 'asaurex':ti,ab,kw OR 'azucimet':ti,ab,kw OR 'belomet':ti,ab,kw OR 'biomag':ti,ab,kw OR 'biomet':ti,ab,kw OR 'brumetidina':ti,ab,kw OR 'campanex':ti,ab,kw OR 'cemedin*':ti,ab,kw OR 'cementin':ti,ab,kw OR 'ciclem':ti,ab,kw OR 'cigamet':ti,ab,kw OR 'cignatin':ti,ab,kw OR 'ciket m':ti,ab,kw OR 'cimal':ti,ab,kw OR 'cimbene':ti,ab,kw OR 'cimedine':ti,ab,kw OR 'cimehexal':ti,ab,kw OR 'cimeldine':ti,ab,kw OR 'cimeloc':ti,ab,kw OR 'cimet*':ti,ab,kw OR 'cimetum':ti,ab,kw OR 'cimewell':ti,ab,kw OR 'cimlok':ti,ab,kw OR 'cimulcer':ti,ab,kw OR 'cinadine':ti,ab,kw OR 'cinulcus':ti,ab,kw OR 'cismetin':ti,ab,kw OR 'citidine':ti,ab,kw OR 'citius':ti,ab,kw OR 'contracid':ti,ab,kw OR 'corsamet':ti,ab,kw OR 'cylock':ti,ab,kw OR 'cytine':ti,ab,kw OR 'dispamet':ti,ab,kw OR 'duomet':ti,ab,kw OR 'dyspamet':ti,ab,kw OR 'edalene':ti,ab,kw OR 'eladene':ti,ab,kw OR 'erlmetin':ti,ab,kw OR 'eureceptor':ti,ab,kw OR 'fremet':ti,ab,kw OR 'gastab':ti,ab,kw OR 'gastidine':ti,ab,kw OR 'gastrobitan':ti,ab,kw OR 'gastroprotect':ti,ab,kw OR 'gawei':ti,ab,kw OR 'getidin':ti,ab,kw OR 'haldin':ti,ab,kw OR 'hexamet':ti,ab,kw OR 'himetin':ti,ab,kw OR 'histodil':ti,ab,kw OR 'inesfay':ti,ab,kw OR 'lenamet*':ti,ab,kw OR 'lock 2':ti,ab,kw OR 'magicul':ti,ab,kw OR 'manomet':ti,ab,kw OR 'maritidine':ti,ab,kw OR 'med-gastramet':ti,ab,kw OR 'meticon':ti,ab,kw OR 'neutromed':ti,ab,kw OR 'neutronorm*':ti,ab,kw OR 'novocimetine':ti,ab,kw OR 'nulcer':ti,ab,kw OR 'peptol':ti,ab,kw OR 'piovalen':ti,ab,kw OR 'pondarmett':ti,ab,kw OR 'powegon':ti,ab,kw OR 'promet':ti,ab,kw OR 'sanmetidin':ti,ab,kw OR 'secapine':ti,ab,kw OR 'senoba':ti,ab,kw OR 'sertidine':ti,ab,kw OR 'shintamet':ti,ab,kw OR 'siamidine':ti,ab,kw OR 'sigmetadine':ti,ab,kw OR 'simaglen':ti,ab,kw OR 'stogamet':ti,ab,kw OR 'stomakon':ti,ab,kw OR 'stomedine':ti,ab,kw OR 'stomet':ti,ab,kw OR 'tagagel':ti,ab,kw OR 'tagamet*':ti,ab,kw OR 'tagma':ti,ab,kw OR 'tametin':ti,ab,kw OR 'tobymet':ti,ab,kw OR 'ulcedin*':ti,ab,kw OR 'ulcemet':ti,ab,kw OR 'ulcenon':ti,ab,kw OR 'ulcerfen':ti,ab,kw OR 'ulcidine':ti,ab,kw OR 'ulcim*':ti,ab,kw OR 'ulcodina':ti,ab,kw OR 'ulcolind h2':ti,ab,kw OR 'ulcomedina':ti,ab,kw OR 'ulcomet':ti,ab,kw OR 'ulcumet':ti,ab,kw OR 'ulsikur':ti,ab,kw OR 'umamett':ti,ab,kw OR 'weisdin':ti,ab,kw OR 'xepamet':ti,ab,kw OR 'zymerol':ti,ab,kw)

Antiepileptics

('anticonvulsive agent'/exp OR 'anticonvuls*':ti,ab,kw OR 'anti-convuls*':ti,ab,kw OR 'AED':ti,ab,kw OR 'AEDs':ti,ab,kw OR 'antiepilep*':ti,ab,kw OR 'anti-epilep*':ti,ab,kw OR 'anti-seizure':ti,ab,kw OR 'antiseizure':ti,ab,kw OR 'benzodiazepine'/exp OR 'benzodiazepin*':ti,ab,kw OR 'barbituric acid derivative'/exp OR 'barbiturat*':ti,ab,kw OR 'brivaracetam'/exp OR 'brivaracetam':ti,ab,kw OR 'Briviact':ti,ab,kw OR 'brivlera':ti,ab,kw OR 'nubriveo':ti,ab,kw OR 'rikelta':ti,ab,kw OR 'midazolam'/exp OR 'midazolam*':ti,ab,kw OR 'midazolam*':ti,ab,kw OR 'Dormicum':ti,ab,kw OR 'Dormire':ti,ab,kw OR 'Epistatus':ti,ab,kw OR 'Fulsed':ti,ab,kw OR 'Garen':ti,ab,kw OR 'Hypnovel':ti,ab,kw OR 'Ipnovel':ti,ab,kw OR 'Versed':ti,ab,kw OR 'Dalam':ti,ab,kw OR 'Nocturna':ti,ab,kw OR 'Setam':ti,ab,kw OR 'propofol'/exp OR 'clonazepam'/exp OR 'diazepam'/exp OR 'carbamazepine'/exp OR 'valproic acid'/exp OR 'levetiracetam'/exp OR 'phenobarbital'/exp OR 'lamotrigine'/exp OR 'lacosamide'/exp OR 'cannabidiol'/exp OR 'cenobamate'/exp OR 'clobazam'/exp OR 'eslicarbazepine'/exp OR 'ethosuximide'/exp OR 'retigabine'/exp OR 'felbamate'/exp OR 'fenfluramine'/exp OR 'gabapentin'/exp OR 'lacosamide'/exp OR 'lorazepam'/exp OR 'oxcarbazepine'/exp OR 'perampanel'/exp OR 'phenytoin'/exp OR 'pregabalin'/exp OR 'primidone'/exp OR 'rufinamide'/exp OR 'stiripentol'/exp OR 'tiagabine'/exp OR 'topiramate'/exp OR 'vigabatrin'/exp OR 'zonisamide'/exp OR ‘Propofol*’:ti,ab,kw OR ‘Disoprofol’:ti,ab,kw OR ‘Diprivan’:ti,ab,kw OR ‘Disoprivan’:ti,ab,kw OR ‘Fresofol’:ti,ab,kw OR ‘Ivofol’:ti,ab,kw OR ‘Recofol’:ti,ab,kw OR ‘Aquafol’:ti,ab,kw OR ‘Recofol’:ti,ab,kw OR ‘Anepol’:ti,ab,kw OR ‘Hypro’:ti,ab,kw OR ‘Lipuro’:ti,ab,kw OR ‘Plofed’:ti,ab,kw OR ‘Profol’:ti,ab,kw OR ‘Propofil’:ti,ab,kw OR ‘Propolipid’:ti,ab,kw OR ‘Propovan’:ti,ab,kw OR ‘Propoven’:ti,ab,kw OR ‘Provive’:ti,ab,kw OR ‘clonazepam*’:ti,ab,kw OR ‘Rivotril’:ti,ab,kw OR ‘Klonopin’:ti,ab,kw OR ‘Antelepsin’:ti,ab,kw OR ‘diazepam*’:ti,ab,kw OR ‘Diazemuls’:ti,ab,kw OR ‘Faustan’:ti,ab,kw OR ‘Valium’:ti,ab,kw OR ‘Seduxen’:ti,ab,kw OR ‘Sibazon’:ti,ab,kw OR ‘Stesolid’:ti,ab,kw OR ‘Apaurin’:ti,ab,kw OR ‘Relanium’:ti,ab,kw OR ‘carbamazepin*’:ti,ab,kw OR ‘Carbazepin’:ti,ab,kw OR ‘Epitol’:ti,ab,kw OR ‘Finlepsin’:ti,ab,kw OR ‘Neurotol’:ti,ab,kw OR ‘Tegretol’:ti,ab,kw OR ‘Amizepine’:ti,ab,kw OR ‘valproic*’:ti,ab,kw OR ‘Divalproex*’:ti,ab,kw OR ‘Depakene’:ti,ab,kw OR ‘Depakine’:ti,ab,kw OR ‘Convulsofin’:ti,ab,kw OR ‘Depakote’:ti,ab,kw OR ‘Dipropyl Acetate’:ti,ab,kw OR ‘valproate*’:ti,ab,kw OR ‘Vupral’:ti,ab,kw OR ‘Propylisopropylacetic Acid’:ti,ab,kw OR ‘Ergenyl’:ti,ab,kw OR ‘levetiracetam*’:ti,ab,kw OR ‘Etiracetam’:ti,ab,kw OR ‘Keppra’:ti,ab,kw OR ‘phenobarbit*’:ti,ab,kw OR ‘Phenylbarbital’:ti,ab,kw OR ‘Phenylethylbarbituric Acid’:ti,ab,kw OR ‘Phenemal’:ti,ab,kw OR ‘Phenobarbitone’:ti,ab,kw OR ‘Hysteps’:ti,ab,kw OR ‘Luminal’:ti,ab,kw OR ‘Gardenal’:ti,ab,kw OR ‘lamotrigi*’:ti,ab,kw OR ‘Lamictal’:ti,ab,kw OR ‘Crisomet’:ti,ab,kw OR ‘Lamiktal’:ti,ab,kw OR ‘Labileno’:ti,ab,kw OR ‘lacosamide’:ti,ab,kw OR ‘Vimpat’:ti,ab,kw OR ‘cannabidiol’:ti,ab,kw OR ‘Epidiolex’:ti,ab,kw OR ‘cenobamat*’:ti,ab,kw OR ‘XCOPRI’:ti,ab,kw OR ‘clobazam*’:ti,ab,kw OR ‘Onfi’:ti,ab,kw OR ‘Urbanyl’:ti,ab,kw OR ‘Frisium’:ti,ab,kw OR ‘eslicarbazepine’:ti,ab,kw OR ‘ethosuximid*’:ti,ab,kw OR ‘ethosuccimid*’:ti,ab,kw OR ‘ethylmethylsuccimide’:ti,ab,kw OR ‘Ethymal’:ti,ab,kw OR ‘Zarontin’:ti,ab,kw OR ‘Petnidan’:ti,ab,kw OR ‘Pyknolepsinum’:ti,ab,kw OR ‘Suksilep’:ti,ab,kw OR ‘Suxilep’:ti,ab,kw OR ‘Emeside’:ti,ab,kw OR ‘Etosuximida Faes’:ti,ab,kw OR ‘ezogabine’:ti,ab,kw OR ‘retigabin*’:ti,ab,kw OR ‘Potiga’:ti,ab,kw OR ‘felbamat*’:ti,ab,kw OR ‘Taloxa’:ti,ab,kw OR ‘Felbatol’:ti,ab,kw OR ‘Felbamyl’:ti,ab,kw OR ‘fenfluramin*’:ti,ab,kw OR ‘Pondimin’:ti,ab,kw OR ‘Isomeride’:ti,ab,kw OR ‘Fintepla’:ti,ab,kw OR ‘gabapenti*’:ti,ab,kw OR ‘NovoGabapentin’:ti,ab,kw OR ‘ApoGabapentin’:ti,ab,kw OR ‘Neurontin’:ti,ab,kw OR ‘Convalis’:ti,ab,kw OR ‘lacosamid*’:ti,ab,kw OR ‘Vimpat’:ti,ab,kw OR ‘lorazepam*’:ti,ab,kw OR ‘Ativan’:ti,ab,kw OR ‘Orfidal Wyeth’:ti,ab,kw OR ‘Témesta’:ti,ab,kw OR ‘Tolid’:ti,ab,kw OR ‘Donix’:ti,ab,kw OR ‘Duralozam’:ti,ab,kw OR ‘Durazolam’:ti,ab,kw OR ‘Idalprem’:ti,ab,kw OR ‘Laubeel’:ti,ab,kw OR ‘Lorazep Von Ct’:ti,ab,kw OR ‘Novo-Lorazem’:ti,ab,kw OR ‘Nu-Loraz’:ti,ab,kw OR ‘Sedicepan’:ti,ab,kw OR ‘Sinestron’:ti,ab,kw OR ‘Somagerol’:ti,ab,kw OR ‘Temesta’:ti,ab,kw OR ‘oxcarbazepine*’:ti,ab,kw OR ‘Trileptal’:ti,ab,kw OR ‘Timox’:ti,ab,kw OR ‘perampanel*’:ti,ab,kw OR ‘Fycompa’:ti,ab,kw OR ‘phenytoin*’:ti,ab,kw OR ‘Fenitoin’:ti,ab,kw OR ‘Diphenylhydantoin’:ti,ab,kw OR ‘Difenin’:ti,ab,kw OR ‘Dihydan’:ti,ab,kw OR ‘Phenhydan’:ti,ab,kw OR ‘Epamin’:ti,ab,kw OR ‘Epanutin’:ti,ab,kw OR ‘Hydantol’:ti,ab,kw OR ‘Sodium Diphenylhydantoinate’:ti,ab,kw OR ‘Antisacer’:ti,ab,kw OR ‘Dilantin’:ti,ab,kw OR ‘pregabalin*’:ti,ab,kw OR ‘Lyrica’:ti,ab,kw OR ‘primidon*’:ti,ab,kw OR ‘primidon*’:ti,ab,kw OR ‘Liskantin’:ti,ab,kw OR ‘Mylepsinum’:ti,ab,kw OR ‘Resimatil’:ti,ab,kw OR ‘Desoxyphenobarbital’:ti,ab,kw OR ‘Mizodin’:ti,ab,kw OR ‘Mysoline’:ti,ab,kw OR ‘Primaclone’:ti,ab,kw OR ‘Sertan’:ti,ab,kw OR ‘Misodine’:ti,ab,kw OR ‘rufinamid*’:ti,ab,kw OR ‘Inovelon’:ti,ab,kw OR ‘stiripentol*’:ti,ab,kw OR ‘Diacomit’:ti,ab,kw OR ‘tiagabin*’:ti,ab,kw OR ‘Gabitril’:ti,ab,kw OR ‘topiramat*’:ti,ab,kw OR ‘Epitomax’:ti,ab,kw OR ‘Topamax’:ti,ab,kw OR ‘vigabatrin*’:ti,ab,kw OR ‘Sabrilex’:ti,ab,kw OR ‘Sabril’:ti,ab,kw OR ‘zonisamid*’:ti,ab,kw OR ‘Zonegran’:ti,ab,kw)

Antidepressants

('antidepressant agent'/exp OR 'antidepressive*':ti,ab,kw OR 'antidepressant*':ti,ab,kw OR 'antidepression*':ti,ab,kw OR 'anti depressant*':ti,ab,kw OR 'serotonin uptake inhibitor'/exp OR 'adrenergic receptor affecting agent'/exp OR 'serotonin noradrenalin reuptake inhibitor'/exp OR 'mirtazapine'/exp OR 'amfebutamone'/exp OR 'duloxetine'/exp OR 'venlafaxine'/exp OR 'sertraline'/exp OR 'fluoxetine'/exp OR 'paroxetine'/exp OR 'fluvoxamine'/exp OR 'escitalopram'/exp OR 'citalopram'/exp OR 'trazodone'/exp OR 'nortriptyline'/exp OR 'amitriptyline'/exp OR 'clomipramine'/exp OR 'doxepin'/exp OR 'dosulepin'/exp OR 'maprotiline'/exp OR 'imipramine'/exp OR ‘serotonin reuptake inhibitor*’:ti,ab,kw OR ‘serotonin specific reuptake inhibitor*’:ti,ab,kw OR ‘serotonin uptake inhibitor*’:ti,ab,kw OR ‘5-hydroxytryptamine uptake inhibitor*’:ti,ab,kw OR ‘5-HT uptake inhibitor*’:ti,ab,kw OR ‘SSRI*’:ti,ab,kw OR ‘second-generation antidepress*’:ti,ab,kw OR ‘atypical antidepress*’:ti,ab,kw OR ‘serotonin and noradrenaline reuptake inhibitor*’:ti,ab,kw OR ‘serotonin and noradrenaline uptake inhibitor*’:ti,ab,kw OR ‘serotonin noradrenaline reuptake inhibitor*’:ti,ab,kw OR ‘serotonin noradrenaline uptake inhibitor*’:ti,ab,kw OR ‘dual reuptake inhibitor*’:ti,ab,kw OR ‘dual uptake inhibitor*’:ti,ab,kw OR ‘citalopram’:ti,ab,kw OR ‘escitalopram’:ti,ab,kw OR ‘fluvoxamine’:ti,ab,kw OR ‘paroxetine’:ti,ab,kw OR ‘fluoxetine’:ti,ab,kw OR ‘sertraline’:ti,ab,kw OR ‘serotonin and norepinephrine reuptake inhibitor*’:ti,ab,kw OR ‘serotonin and norepinephrine uptake inhibitor*’:ti,ab,kw OR ‘serotonin norepinephrine reuptake inhibitor*’:ti,ab,kw OR ‘serotonin norepinephrine uptake inhibitor*’:ti,ab,kw OR ‘SNRI*’:ti,ab,kw OR ‘SSNRI*’:ti,ab,kw OR ‘venlafaxine’:ti,ab,kw OR ‘duloxetine’:ti,ab,kw OR ‘mirtazapine’:ti,ab,kw OR ‘trazodone’:ti,ab,kw OR ‘bupropion’:ti,ab,kw OR ‘amfebutamone’:ti,ab,kw OR ‘nortriptyline’:ti,ab,kw OR ‘desitriptyline’:ti,ab,kw OR ‘desmethylamitriptylin’:ti,ab,kw OR ‘Allegron’:ti,ab,kw OR ‘Aventyl’:ti,ab,kw OR ‘Paxtibi’:ti,ab,kw OR ‘Nortrilen’:ti,ab,kw OR ‘Pamelor’:ti,ab,kw OR ‘Norfenazin’:ti,ab,kw OR ‘amitrip*’:ti,ab,kw OR ‘Tryptine’:ti,ab,kw OR ‘Amineurin’:ti,ab,kw OR ‘Amitrip’:ti,ab,kw OR ‘Amitrol’:ti,ab,kw OR ‘Anapsique’:ti,ab,kw OR ‘Damilen’:ti,ab,kw OR ‘Domical’:ti,ab,kw OR ‘Laroxyl’:ti,ab,kw OR ‘Lentizol’:ti,ab,kw OR ‘Novoprotect’:ti,ab,kw OR ‘Saroten’:ti,ab,kw OR ‘Sarotex’:ti,ab,kw OR ‘Syneudon’:ti,ab,kw OR ‘Triptafen’:ti,ab,kw OR ‘Endep’:ti,ab,kw OR ‘Tryptizol’:ti,ab,kw OR ‘Elavil’:ti,ab,kw OR ‘Tryptanol’:ti,ab,kw OR ‘clomipramine*’:ti,ab,kw OR ‘chlomipramine’:ti,ab,kw OR ‘chlorimipramine’:ti,ab,kw OR ‘Hydiphen’:ti,ab,kw OR ‘Anafranil’:ti,ab,kw OR ‘doxepi*’:ti,ab,kw OR ‘Deptran’:ti,ab,kw OR ‘Desidox’:ti,ab,kw OR ‘ Doneurin’:ti,ab,kw OR ‘Espadox’:ti,ab,kw OR ‘Mareen’:ti,ab,kw OR ‘Prudoxin’:ti,ab,kw OR ‘Quitaxon’:ti,ab,kw OR ‘Sinequan’:ti,ab,kw OR ‘Sinquan’:ti,ab,kw OR ‘Zonalon’:ti,ab,kw OR ‘Xepin’:ti,ab,kw OR ‘Aponal’:ti,ab,kw OR ‘Xepin’:ti,ab,kw OR ‘dothiepin’:ti,ab,kw OR ‘dothiepin*’:ti,ab,kw OR ‘dosulepin’:ti,ab,kw OR ‘Prothiaden’:ti,ab,kw OR ‘Maprotilin*’:ti,ab,kw OR ‘Novo-Maprotiline’:ti,ab,kw OR ‘Dibencycladine’:ti,ab,kw OR ‘Psymion’:ti,ab,kw OR ‘Ludiomil’:ti,ab,kw OR ‘Maprolu’:ti,ab,kw OR ‘Mirpan’:ti,ab,kw OR ‘Deprilept’:ti,ab,kw OR ‘imipramine*’:ti,ab,kw OR ‘norchlorimipramine’:ti,ab,kw OR ‘imidobenzyle’:ti,ab,kw OR ‘Imizin’:ti,ab,kw OR ‘Tofranil’:ti,ab,kw OR ‘Janimine’:ti,ab,kw OR ‘Melipramine’:ti,ab,kw OR ‘Pryleugan’:ti,ab,kw)

Lithium

('lithium'/exp OR 'lithium derivative'/exp OR 'tranquilizer'/exp OR 'lithium':ti,ab,kw OR 'antimanic*':ti,ab,kw OR 'lithium carbonate'/exp OR ‘camcolit’:ti,ab,kw OR ‘candamide’:ti,ab,kw OR ‘carbolit*’:ti,ab,kw OR ‘carbonate lithium’:ti,ab,kw OR ‘ceglution*’:ti,ab,kw OR ‘contemnol’:ti,ab,kw OR ‘cp 15, 467 61’:ti,ab,kw OR ‘cp 15467 61’:ti,ab,kw OR ‘cp15, 467 61’:ti,ab,kw OR ‘cp15467 61’:ti,ab,kw OR ‘duralith’:ti,ab,kw OR ‘eskalith*’:ti,ab,kw OR ‘hynorex*’:ti,ab,kw OR ‘lentolith’:ti,ab,kw OR ‘licab’:ti,ab,kw OR ‘licarb*’:ti,ab,kw OR ‘lidin’:ti,ab,kw OR ‘limas’:ti,ab,kw OR ‘linthane’:ti,ab,kw OR ‘liskonium’:ti,ab,kw OR ‘liskonum’:ti,ab,kw OR ‘lithane’:ti,ab,kw OR ‘litheum 300’:ti,ab,kw OR ‘lithicarb’:ti,ab,kw OR ‘lithionate’:ti,ab,kw OR ‘lithmax’:ti,ab,kw OR ‘lithobid’:ti,ab,kw OR ‘lithocap’:ti,ab,kw OR ‘lithocarb’:ti,ab,kw OR ‘lithonate’:ti,ab,kw OR ‘lithotabs’:ti,ab,kw OR ‘litilent’:ti,ab,kw OR ‘litocarb’:ti,ab,kw OR ‘maniprex’:ti,ab,kw OR ‘neurolepsin’:ti,ab,kw OR ‘nsc 16895’:ti,ab,kw OR ‘nsc 16985’:ti,ab,kw OR ‘phanate’:ti,ab,kw OR ‘plenur’:ti,ab,kw OR ‘priadel*’:ti,ab,kw OR ‘quilonium-r’:ti,ab,kw OR ‘quilonorm*’:ti,ab,kw OR ‘quilonum*’:ti,ab,kw OR ‘teralithe’:ti,ab,kw OR ‘theralite’:ti,ab,kw OR ‘theralithe lp’:ti,ab,kw)

Stimulants

('methylphenidate'/exp OR 'methylphenidate*':ti,ab,kw OR 'methylphenydate*':ti,ab,kw OR 'methylphenindate*':ti,ab,kw OR 'methylfenidaat':ti,ab,kw OR 'methylfenidate':ti,ab,kw OR 'methyl phenidate':ti,ab,kw OR 'Addepta*':ti,ab,kw OR 'adhansia*':ti,ab,kw OR 'affenid*':ti,ab,kw OR 'aptensio*':ti,ab,kw OR 'atenza*':ti,ab,kw OR 'attenta':ti,ab,kw OR 'benjorna':ti,ab,kw OR 'biphentin':ti,ab,kw OR 'centedrin':ti,ab,kw OR 'concerta*':ti,ab,kw OR 'cotempla*':ti,ab,kw OR 'daytrana':ti,ab,kw OR 'delmosart':ti,ab,kw OR 'difumenil':ti,ab,kw OR 'equasym*':ti,ab,kw OR 'exattent*':ti,ab,kw OR 'focusim xl':ti,ab,kw OR 'foquest':ti,ab,kw OR 'jornay*':ti,ab,kw OR 'kinecteen':ti,ab,kw OR 'kixel*':ti,ab,kw OR 'matoride xl':ti,ab,kw OR 'medanef':ti,ab,kw OR 'medicebran':ti,ab,kw OR 'medikinet*':ti,ab,kw OR 'mefinad':ti,ab,kw OR 'metadate*':ti,ab,kw OR 'methylin*':ti,ab,kw OR 'methypatch':ti,ab,kw OR 'methysym*':ti,ab,kw OR 'metyrol*':ti,ab,kw OR 'motiron':ti,ab,kw OR 'penid':ti,ab,kw OR 'phenidyl*':ti,ab,kw OR 'quasym*':ti,ab,kw OR 'quillichew*':ti,ab,kw OR 'quillivant*':ti,ab,kw OR 'relexxii':ti,ab,kw OR 'rilatin*':ti,ab,kw OR 'ritalin*':ti,ab,kw OR 'ritaphen':ti,ab,kw OR 'rubicrono':ti,ab,kw OR 'rubifen*':ti,ab,kw OR 'tranquilyn':ti,ab,kw OR 'tsentedrin':ti,ab,kw OR 'xaggitin xl':ti,ab,kw OR 'xenidate xl':ti,ab,kw)

Antihistamines

('antihistaminic agent'/exp OR 'antihistamine*':ti,ab,kw OR 'anti-histamine*':ti,ab,kw OR 'antihistaminic*':ti,ab,kw OR 'anti-histaminic*':ti,ab,kw OR 'histamine antagonist*':ti,ab,kw OR 'histamine receptor antagonist*':ti,ab,kw OR 'histamine block*':ti,ab,kw OR 'histamine receptor block*':ti,ab,kw OR 'histamine H1 antagonist*':ti,ab,kw OR 'histamine H2 antagonist*':ti,ab,kw OR 'histamine H3 antagonist*':ti,ab,kw OR 'histamine H4 antagonist*':ti,ab,kw OR 'histamine H1 receptor antagonist*':ti,ab,kw OR 'histamine H2 receptor antagonist*':ti,ab,kw OR 'histamine H3 receptor antagonist*':ti,ab,kw OR 'histamine H4 receptor antagonist*':ti,ab,kw OR 'H1 antagonist*':ti,ab,kw OR 'H2 antagonist*':ti,ab,kw OR 'H3 antagonist*':ti,ab,kw OR 'H4 antagonist*':ti,ab,kw OR 'H1 receptor antagonist*':ti,ab,kw OR 'H2 receptor antagonist*':ti,ab,kw OR 'H3 receptor antagonist*':ti,ab,kw OR 'H4 receptor antagonist*':ti,ab,kw OR 'histamine H1 receptor block*':ti,ab,kw OR 'histamine H2 receptor block*':ti,ab,kw OR 'histamine H3 receptor block*':ti,ab,kw OR 'histamine H4 receptor block*':ti,ab,kw OR 'histamine H1 block*':ti,ab,kw OR 'histamine H2 block*':ti,ab,kw OR 'histamine H3 block*':ti,ab,kw OR 'histamine H4 block*':ti,ab,kw OR 'hydroxyzine'/exp OR 'hydroxyzin*':ti,ab,kw OR 'Atarax*':ti,ab,kw OR 'Aterax':ti,ab,kw OR 'Attarax':ti,ab,kw OR 'Bestalin':ti,ab,kw OR 'Bobsule':ti,ab,kw OR 'Centilax':ti,ab,kw OR 'Cerax':ti,ab,kw OR 'Darax':ti,ab,kw OR 'Disron':ti,ab,kw OR 'Dormirex':ti,ab,kw OR 'Durrax':ti,ab,kw OR 'Efidac':ti,ab,kw OR 'Hiderax':ti,ab,kw OR 'Hizin':ti,ab,kw OR 'Hyzine':ti,ab,kw OR 'Idroxizina':ti,ab,kw OR 'Iremofar':ti,ab,kw OR 'Iterax':ti,ab,kw OR 'Orgatrax':ti,ab,kw OR 'Otarex':ti,ab,kw OR 'Paxistil':ti,ab,kw OR 'Phymorax':ti,ab,kw OR 'Postarax':ti,ab,kw OR 'Prurid':ti,ab,kw OR 'Qualidrozine':ti,ab,kw OR 'Quiess':ti,ab,kw OR 'R-Rax':ti,ab,kw OR 'Tran-Q':ti,ab,kw OR 'Trandozine':ti,ab,kw OR 'Tranquijust':ti,ab,kw OR 'Ucerax':ti,ab,kw OR 'Unamine':ti,ab,kw OR 'Vistacot':ti,ab,kw OR 'Vistaject-50':ti,ab,kw OR 'Vistaril*':ti,ab,kw OR 'acrivastine'/exp OR 'acrivastine*':ti,ab,kw OR 'Prolert':ti,ab,kw OR 'Semprex':ti,ab,kw OR 'azelastine'/exp OR 'azelastin*':ti,ab,kw OR 'Acatar Allergy':ti,ab,kw OR 'Afluon*':ti,ab,kw OR 'Alastine':ti,ab,kw OR 'Alerdual':ti,ab,kw OR 'Alergodil':ti,ab,kw OR 'Allergo-azelind':ti,ab,kw OR 'Allergodil*':ti,ab,kw OR 'Allergodrop':ti,ab,kw OR 'Allergospray':ti,ab,kw OR 'Allespray':ti,ab,kw OR 'Allestin':ti,ab,kw OR 'Amelor':ti,ab,kw OR 'Antalerg':ti,ab,kw OR 'Astelin':ti,ab,kw OR 'Astepro*':ti,ab,kw OR 'Azedil':ti,ab,kw OR 'Azel-drop*':ti,ab,kw OR 'Azela vision*':ti,ab,kw OR 'Azelamed':ti,ab,kw OR 'Azelastin*':ti,ab,kw OR 'Azelavision*':ti,ab,kw OR 'Azella':ti,ab,kw OR 'Azelsan':ti,ab,kw OR 'Azep*':ti,ab,kw OR 'Carelastin':ti,ab,kw OR 'Corifina':ti,ab,kw OR 'Lasticom':ti,ab,kw OR 'Lastin*':ti,ab,kw OR 'Loxin':ti,ab,kw OR 'Oculastin':ti,ab,kw OR 'Optilast':ti,ab,kw OR 'Optivar':ti,ab,kw OR 'Pollival':ti,ab,kw OR 'Proallergodil':ti,ab,kw OR 'Radethacin':ti,ab,kw OR 'Radethazin':ti,ab,kw OR 'Rhinolast*':ti,ab,kw OR 'Rinelaz':ti,ab,kw OR 'Snizdil':ti,ab,kw OR 'Tebarat':ti,ab,kw OR 'Visuzel':ti,ab,kw OR 'Vividrin akut*':ti,ab,kw OR 'Vivispray':ti,ab,kw OR 'azatadine'/exp OR 'azatadin*':ti,ab,kw OR 'Idulamine':ti,ab,kw OR 'Idulian':ti,ab,kw OR 'Lergocil':ti,ab,kw OR 'Nalomet':ti,ab,kw OR 'Optimine':ti,ab,kw OR 'Verben':ti,ab,kw OR 'Zadine':ti,ab,kw OR 'brompheniramine'/exp OR 'brompheniramin*':ti,ab,kw OR 'para-bromdylamine':ti,ab,kw OR 'p-Bromdylamine*':ti,ab,kw OR 'Babycold':ti,ab,kw OR 'Bomex':ti,ab,kw OR 'Bomine':ti,ab,kw OR 'Brombay':ti,ab,kw OR 'Bromdylamine':ti,ab,kw OR 'Bromfeniramine':ti,ab,kw OR 'Brommine':ti,ab,kw OR 'Bromoprophenpyridamine':ti,ab,kw OR 'Bromprophenpyridamine':ti,ab,kw OR 'Broramin':ti,ab,kw OR 'Brovex*':ti,ab,kw OR 'Codimal*':ti,ab,kw OR 'Colhist':ti,ab,kw OR 'Dimegan':ti,ab,kw OR 'Dimetane*':ti,ab,kw OR 'Dimigan':ti,ab,kw OR 'Dimotane':ti,ab,kw OR 'Dimetapp*':ti,ab,kw OR 'Ilvin':ti,ab,kw OR 'Lodrane*':ti,ab,kw OR 'Nasahist*':ti,ab,kw OR 'Nd stat':ti,ab,kw OR 'Oraminic-2':ti,ab,kw OR 'Parabromdylamine':ti,ab,kw OR 'Veltane':ti,ab,kw OR 'carbinoxamine'/exp OR 'carbinoxamin*':ti,ab,kw OR 'paracarbinoxamin*':ti,ab,kw OR 'Allergefon':ti,ab,kw OR 'Polistine':ti,ab,kw OR 'Pyrate':ti,ab,kw OR 'Shelton':ti,ab,kw OR 'Tilford':ti,ab,kw OR 'Twiston':ti,ab,kw OR 'Ziriton':ti,ab,kw OR 'cetirizine'/exp OR ‘cetirizin*’:ti,ab,kw OR ‘Acidrine’:ti,ab,kw OR ‘Actifed allergie’:ti,ab,kw OR ‘Adezio’:ti,ab,kw OR ‘Agelmin’:ti,ab,kw OR ‘Alairgix*’:ti,ab,kw OR ‘Alercet’:ti,ab,kw OR ‘Alerid’:ti,ab,kw OR ‘Alerlisin’:ti,ab,kw OR ‘Alertop’:ti,ab,kw OR ‘Alerviden’:ti,ab,kw OR ‘Aletir’:ti,ab,kw OR ‘Alled’:ti,ab,kw OR ‘Allertec’:ti,ab,kw OR ‘Alltec’:ti,ab,kw OR ‘Alzytec’:ti,ab,kw OR ‘Benaday’:ti,ab,kw OR ‘Benadryl*’:ti,ab,kw OR ‘Betarhin’:ti,ab,kw OR ‘Cabal’:ti,ab,kw OR ‘Cerazine’:ti,ab,kw OR ‘Cerini’:ti,ab,kw OR ‘Cerotec’:ti,ab,kw OR ‘Cesta’:ti,ab,kw OR ‘Cetalerg’:ti,ab,kw OR ‘Piriteze*’:ti,ab,kw OR ‘Virlix’:ti,ab,kw OR ‘Zetir’:ti,ab,kw OR ‘Zyrtec’:ti,ab,kw OR ‘Reactine’:ti,ab,kw OR ‘Voltric*’:ti,ab,kw OR ‘Zirtek’:ti,ab,kw OR ‘Alerlisin’:ti,ab,kw OR ‘Cetalerg’:ti,ab,kw OR ‘Ceterifug’:ti,ab,kw OR ‘Cethis’:ti,ab,kw OR ‘Cetimin’:ti,ab,kw OR ‘Cetin’:ti,ab,kw OR ‘Cetirax’:ti,ab,kw OR ‘Cetirin’:ti,ab,kw OR ‘Ceti TAD’:ti,ab,kw OR ‘Ceti-Puren’:ti,ab,kw OR ‘Cetiderm’:ti,ab,kw OR ‘Cetidura’:ti,ab,kw OR ‘Cetil von ct’:ti,ab,kw OR ‘CetiLich’:ti,ab,kw OR ‘Cetirigamma’:ti,ab,kw OR ‘Cetirlan’:ti,ab,kw OR ‘Cetrimed’:ti,ab,kw OR ‘Cetrine’:ti,ab,kw OR ‘Cetrizet’:ti,ab,kw OR ‘Cetrizin’:ti,ab,kw OR ‘Cetymin’:ti,ab,kw OR ‘Chlorphed’:ti,ab,kw OR ‘Cistamine’:ti,ab,kw OR ‘Deallergy’:ti,ab,kw OR ‘Drill allergie’:ti,ab,kw OR ‘Em pharma daily hayfever’:ti,ab,kw OR ‘Falergi’:ti,ab,kw OR ‘Finallerg’:ti,ab,kw OR ‘Formistin’:ti,ab,kw OR ‘Generit’:ti,ab,kw OR ‘Histazine’:ti,ab,kw OR ‘Histica’:ti,ab,kw OR ‘Incidal*’:ti,ab,kw OR ‘Lergium’:ti,ab,kw OR ‘Livoreactine’:ti,ab,kw OR ‘Manx allergy and hayfever relief’:ti,ab,kw OR ‘Nosemin’:ti,ab,kw OR ‘Nosmin’:ti,ab,kw OR ‘Ozen’:ti,ab,kw OR ‘Piriteze allergy’:ti,ab,kw OR ‘Pollenase’:ti,ab,kw OR ‘Pollenshield hayfever relief’:ti,ab,kw OR ‘Prixlae’:ti,ab,kw OR ‘Quzyttir’:ti,ab,kw OR ‘Qzytir’:ti,ab,kw OR ‘Raingen’:ti,ab,kw OR ‘Razene’:ti,ab,kw OR ‘Reactine’:ti,ab,kw OR ‘Rhizin’:ti,ab,kw OR ‘Risima’:ti,ab,kw OR ‘Ritecam’:ti,ab,kw OR ‘Ryvel’:ti,ab,kw OR ‘Ryzen’:ti,ab,kw OR ‘Sancotec’:ti,ab,kw OR ‘Selitex’:ti,ab,kw OR ‘Setizin’:ti,ab,kw OR ‘Simtec’:ti,ab,kw OR ‘Sutac’:ti,ab,kw OR ‘Symitec’:ti,ab,kw OR ‘Terizin’:ti,ab,kw OR ‘Terzine’:ti,ab,kw OR ‘Vick-zyrt’:ti,ab,kw OR ‘Virlix’:ti,ab,kw OR ‘Wockhardt allergy and hayfever relief’:ti,ab,kw OR ‘Zenriz’:ti,ab,kw OR ‘Zensil’:ti,ab,kw OR ‘Zeran’:ti,ab,kw OR ‘Zertine’:ti,ab,kw OR ‘Zerviate’:ti,ab,kw OR ‘Zetir’:ti,ab,kw OR ‘Zicet’:ti,ab,kw OR ‘Zinex’:ti,ab,kw OR ‘Ziralton’:ti,ab,kw OR ‘Zyrtec*’:ti,ab,kw OR ‘Zirtec*’:ti,ab,kw OR ‘Zirtek*’:ti,ab,kw OR ‘Zirtin’:ti,ab,kw OR ‘Zyllergy’:ti,ab,kw OR ‘Zymed’:ti,ab,kw OR ‘Zyrac’:ti,ab,kw OR ‘Zyrazine’:ti,ab,kw OR ‘Zyrcon’:ti,ab,kw OR ‘Zyrlex’:ti,ab,kw OR ‘Zyrtek’:ti,ab,kw OR 'chlorpheniramine'/exp OR 'chlorpheniramin*':ti,ab,kw OR 'chlorprophenpyridamine’:ti,ab,kw OR 'chlorphenamin*’:ti,ab,kw OR 'Allergex’:ti,ab,kw OR 'Allergicin’:ti,ab,kw OR 'Allergosan’:ti,ab,kw OR 'Amichlorphen’:ti,ab,kw OR 'Aller-Chlor’:ti,ab,kw OR 'Chlor-100’:ti,ab,kw OR 'chlorfeniramine’:ti,ab,kw OR 'chloropheniramine’:ti,ab,kw OR 'chloroprofenpyridamine’:ti,ab,kw OR 'chlorphenamin*’:ti,ab,kw OR 'chlorpheneramine’:ti,ab,kw OR 'chlorphenirame’:ti,ab,kw OR 'chlorpheniramine’:ti,ab,kw OR 'chlorphenyramine’:ti,ab,kw OR 'chlorphenizamine’:ti,ab,kw OR "chlorprofenpyridamine’:ti,ab,kw OR 'chlorprophenpyridamide’:ti,ab,kw OR 'chlorprophenpyridamine’:ti,ab,kw OR 'Haymine’:ti,ab,kw OR 'Histafen timed capsule’:ti,ab,kw OR 'Phenamin retard’:ti,ab,kw OR 'Trimeton’:ti,ab,kw OR 'Chlo-Amine’:ti,ab,kw OR 'Chlorpro’:ti,ab,kw OR 'Chlorspan’:ti,ab,kw OR 'Chlortab’:ti,ab,kw OR 'Chlor-Tripolon’:ti,ab,kw OR 'Efidac’:ti,ab,kw OR 'Kloromin*’:ti,ab,kw OR 'Piriton’:ti,ab,kw OR 'Teldrin’:ti,ab,kw OR 'clemastine'/exp OR 'clemastin*':ti,ab,kw OR 'Mecloprodin’:ti,ab,kw OR 'Meclastine’:ti,ab,kw OR 'Neclastine’:ti,ab,kw OR 'Tavist’:ti,ab,kw OR 'Tavegyl*’:ti,ab,kw OR 'Tavegil*’:ti,ab,kw OR 'cinnarizine'/exp OR 'cinna*':ti,ab,kw OR 'Aplactan':ti,ab,kw OR 'Aplexal':ti,ab,kw OR 'Apomitere':ti,ab,kw OR 'Apotomin':ti,ab,kw OR 'Artate':ti,ab,kw OR 'Carecin':ti,ab,kw OR 'Cerebolan':ti,ab,kw OR 'Cerepar':ti,ab,kw OR 'Cibine':ti,ab,kw OR 'Cimarizine':ti,ab,kw OR 'Cinabioquim':ti,ab,kw OR 'Cinaperazine':ti,ab,kw OR 'Cinazière':ti,ab,kw OR 'Cinazyn':ti,ab,kw OR 'Cinnipirine':ti,ab,kw OR 'Cinniprine':ti,ab,kw OR 'Cisaken':ti,ab,kw OR 'Corathiem':ti,ab,kw OR 'Denapol':ti,ab,kw OR 'Dimitron*':ti,ab,kw OR 'Eglen':ti,ab,kw OR 'Giganten':ti,ab,kw OR 'Glanil':ti,ab,kw OR 'Hilactan':ti,ab,kw OR 'Ixertol':ti,ab,kw OR 'Katoseran':ti,ab,kw OR 'Labyrin':ti,ab,kw OR 'Lazeta':ti,ab,kw OR 'Marisan':ti,ab,kw OR 'Midronal':ti,ab,kw OR 'Mitronal':ti,ab,kw OR 'Olamin':ti,ab,kw OR 'Processine':ti,ab,kw OR 'Roin':ti,ab,kw OR 'Sedatromin':ti,ab,kw OR 'Sepan':ti,ab,kw OR 'Siptazin':ti,ab,kw OR 'Spaderizine':ti,ab,kw OR 'Stugeron*':ti,ab,kw OR 'Stutgeron':ti,ab,kw OR 'Stutgin':ti,ab,kw OR 'cyclizine'/exp OR 'cyclizin*':ti,ab,kw OR 'Collox':ti,ab,kw OR 'Echnatol':ti,ab,kw OR 'Gotur':ti,ab,kw OR 'Marazine':ti,ab,kw OR 'Marezine':ti,ab,kw OR 'Marzine':ti,ab,kw OR 'Neo Devomit':ti,ab,kw OR 'Reisfit':ti,ab,kw OR 'Valoid':ti,ab,kw OR 'cyproheptadine'/exp OR 'cyproheptadin*':ti,ab,kw OR 'crypoheptadin*':ti,ab,kw OR 'cryoheptidin*':ti,ab,kw OR 'Adekin':ti,ab,kw OR 'Antergan':ti,ab,kw OR 'Antisemin':ti,ab,kw OR 'Apeton*':ti,ab,kw OR 'Ciplactin':ti,ab,kw OR 'Cipractin':ti,ab,kw OR 'Cyheptine':ti,ab,kw OR 'Cylat':ti,ab,kw OR 'Cypraheptidine':ti,ab,kw OR 'Cypro h':ti,ab,kw OR 'Cyproatin':ti,ab,kw OR 'Cyprogin':ti,ab,kw OR 'Cyprohaptadine':ti,ab,kw OR 'Cyproheptadiene':ti,ab,kw OR 'Cyproheptadin*':ti,ab,kw OR 'Cypromin':ti,ab,kw OR 'Cyprono':ti,ab,kw OR 'Cyprosian':ti,ab,kw OR 'Cytadine':ti,ab,kw OR 'Ciproeptadine':ti,ab,kw OR 'Ciproral':ti,ab,kw OR 'Ciprotol':ti,ab,kw OR 'Ciprovit-a':ti,ab,kw OR 'Cytadine':ti,ab,kw OR 'Dihexazin':ti,ab,kw OR 'Ennamax':ti,ab,kw OR 'Glocyp':ti,ab,kw OR 'Heptasan':ti,ab,kw OR 'Ifrasal':ti,ab,kw OR 'Istam-far':ti,ab,kw OR 'Klarivitina':ti,ab,kw OR 'Kulinet':ti,ab,kw OR 'Nuran':ti,ab,kw OR 'Periacti*':ti,ab,kw OR 'Peritol':ti,ab,kw OR 'Petina':ti,ab,kw OR 'Pilian':ti,ab,kw OR 'Pronicy':ti,ab,kw OR 'Sinapdin':ti,ab,kw OR 'Trimetabol':ti,ab,kw OR 'Vinorex':ti,ab,kw OR 'Viternum':ti,ab,kw OR 'dexchlorpheniramine'/exp OR 'dexchlorpheniramin*':ti,ab,kw OR 'dextrochlorpheniramin*':ti,ab,kw OR 'd chlorpheniramin*':ti,ab,kw OR 'Isomerine':ti,ab,kw OR 'Dexclor':ti,ab,kw OR 'Phendextro':ti,ab,kw OR 'Polaramin*':ti,ab,kw OR 'Polargen TD':ti,ab,kw OR 'Polaronil':ti,ab,kw OR 'dimenhydrinate'/exp OR 'dimenhydrinate*':ti,ab,kw OR 'Amosyt':ti,ab,kw OR 'Anautin*':ti,ab,kw OR 'Andramine*':ti,ab,kw OR 'Andrumin':ti,ab,kw OR 'Antemin':ti,ab,kw OR 'Antimo':ti,ab,kw OR 'Apo-dimenhydrinate':ti,ab,kw OR 'Aviomarin':ti,ab,kw OR 'Biodramina':ti,ab,kw OR 'Bonaling-a':ti,ab,kw OR 'Calm-X':ti,ab,kw OR 'Cinfamar':ti,ab,kw OR 'Contramareo':ti,ab,kw OR 'Chloranautine':ti,ab,kw OR 'Chloronautine':ti,ab,kw OR 'Demodenal':ti,ab,kw OR 'Denim':ti,ab,kw OR 'Detensor':ti,ab,kw OR 'Diamarin':ti,ab,kw OR 'Dimate':ti,ab,kw OR 'Dimen':ti,ab,kw OR 'Dimenate':ti,ab,kw OR 'Dimenhydrinat':ti,ab,kw OR 'Dimin':ti,ab,kw OR 'Dimetabs':ti,ab,kw OR 'Dinate':ti,ab,kw OR 'DMH':ti,ab,kw OR 'diphenhydramine*':ti,ab,kw OR 'Divonal':ti,ab,kw OR 'Dommanate':ti,ab,kw OR 'Dramaban':ti,ab,kw OR 'Dramamine':ti,ab,kw OR 'Dramanate':ti,ab,kw OR 'Dramarin':ti,ab,kw OR 'Dramasan':ti,ab,kw OR 'Dramyl':ti,ab,kw OR 'Drimen':ti,ab,kw OR 'Epha*':ti,ab,kw OR 'Faston':ti,ab,kw OR 'Gravamin':ti,ab,kw OR 'Gravol':ti,ab,kw OR 'Lomarin':ti,ab,kw OR 'Mareol':ti,ab,kw OR 'Marmine':ti,ab,kw OR 'Menhydrinate':ti,ab,kw OR 'Menito':ti,ab,kw OR 'Motion-Aid':ti,ab,kw OR 'Nausex':ti,ab,kw OR 'Nausicalm':ti,ab,kw OR 'Novomin':ti,ab,kw OR 'Paranausine':ti,ab,kw OR 'Pasedol':ti,ab,kw OR 'Reisegold':ti,ab,kw OR 'Reisetabletten*':ti,ab,kw OR 'Rodovan':ti,ab,kw OR 'Rubiemen':ti,ab,kw OR 'Superpep':ti,ab,kw OR 'Travel gum':ti,ab,kw OR 'Travelin':ti,ab,kw OR 'Travelmin':ti,ab,kw OR 'Travel Well':ti,ab,kw OR 'Trawell':ti,ab,kw OR 'Triptone':ti,ab,kw OR 'Valontan':ti,ab,kw OR 'Vomacur':ti,ab,kw OR 'Vomex*':ti,ab,kw OR 'Vomisin':ti,ab,kw OR 'Votmine':ti,ab,kw OR 'Wehamine':ti,ab,kw OR 'Xamamina':ti,ab,kw OR 'dexbrompheniramine'/exp OR 'dexbrompheniramin*':ti,ab,kw OR 'Disomer*':ti,ab,kw OR 'Disophrol':ti,ab,kw OR 'desloratadine'/exp OR 'desloratadin*':ti,ab,kw OR 'descarboethoxyloratadin*':ti,ab,kw OR 'decarbethoxyloratadin*':ti,ab,kw OR 'Aerius':ti,ab,kw OR 'Aerogal':ti,ab,kw OR 'Alerdin':ti,ab,kw OR 'Alergoteva':ti,ab,kw OR 'Aleric deslo':ti,ab,kw OR 'Allex':ti,ab,kw OR 'Aviant':ti,ab,kw OR 'Azomyr':ti,ab,kw OR 'Claramax':ti,ab,kw OR 'Clarinex*':ti,ab,kw OR 'Clarus':ti,ab,kw OR 'Dasergin':ti,ab,kw OR 'Dasmini':ti,ab,kw OR 'Dasselta':ti,ab,kw OR 'Dasseltino':ti,ab,kw OR 'Dassergo':ti,ab,kw OR 'Delortan':ti,ab,kw OR 'Denosin':ti,ab,kw OR 'Desalergo':ti,ab,kw OR 'Desalex':ti,ab,kw OR 'Deslodyna':ti,ab,kw OR 'Deslor*':ti,ab,kw OR 'Deslox':ti,ab,kw OR 'Destamin':ti,ab,kw OR 'Dynid':ti,ab,kw OR 'Escontral':ti,ab,kw OR 'Flynise':ti,ab,kw OR 'Goldesin alerstop':ti,ab,kw OR 'Hitaxa':ti,ab,kw OR 'Jovesto':ti,ab,kw OR 'Kevenix':ti,ab,kw OR 'Lentrica':ti,ab,kw OR 'Loranopro':ti,ab,kw OR 'Lordestin':ti,ab,kw OR 'Lotera':ti,ab,kw OR 'Neoclaritine':ti,ab,kw OR 'Neoclarityn':ti,ab,kw OR 'Opulis':ti,ab,kw OR 'Sinalerg':ti,ab,kw OR 'Spirabel':ti,ab,kw OR 'Supraler':ti,ab,kw OR 'Teslor':ti,ab,kw OR 'Yosqiero':ti,ab,kw OR 'diphenhydramine'/exp OR 'diphenhydrami*':ti,ab,kw OR 'diphenylhydrami*':ti,ab,kw OR ‘diphenydrami*’:ti,ab,kw OR ‘diphendramin*’:ti,ab,kw OR ‘Alledryl’:ti,ab,kw OR ‘Allerdryl’:ti,ab,kw OR ‘Allergical’:ti,ab,kw OR ‘Allergina’:ti,ab,kw OR ‘Amidryl’:ti,ab,kw OR ‘Bagodryl’:ti,ab,kw OR ‘Banaril’:ti,ab,kw OR ‘Beldin’:ti,ab,kw OR ‘Belix’:ti,ab,kw OR ‘Benadril’:ti,ab,kw OR ‘Benadrin’:ti,ab,kw OR ‘Benadryl*’:ti,ab,kw OR ‘Benadyl’:ti,ab,kw OR ‘Benapon’:ti,ab,kw OR ‘Benocten’:ti,ab,kw OR ‘Benodin*’:ti,ab,kw OR ‘Benylan’:ti,ab,kw OR ‘Benzhydramine’:ti,ab,kw OR ‘Benhydramin’:ti,ab,kw OR ‘Ben-hydramin’:ti,ab,kw OR ‘Benadryl’:ti,ab,kw OR ‘Benylin’:ti,ab,kw OR ‘Broncho d’:ti,ab,kw OR ‘Caladryl’:ti,ab,kw OR ‘Carphenamine’:ti,ab,kw OR ‘Carphenex’:ti,ab,kw OR ‘Cathejell’:ti,ab,kw OR ‘Compoz’:ti,ab,kw OR ‘Dermistina’:ti,ab,kw OR ‘Desentol’:ti,ab,kw OR ‘Dibadorm n’:ti,ab,kw OR ‘Dibendrin’:ti,ab,kw OR ‘Dibenil’:ti,ab,kw OR ‘Dibondrin’:ti,ab,kw OR ‘Dibrondrin’:ti,ab,kw OR ‘difenhydramin*’:ti,ab,kw OR ‘Dihidral’:ti,ab,kw OR ‘Dimedrol’:ti,ab,kw OR ‘Dimedryl’:ti,ab,kw OR ‘Dimidril’:ti,ab,kw OR ‘Dimiril’:ti,ab,kw OR ‘Diphantine’:ti,ab,kw OR ‘Diphedryl’:ti,ab,kw OR ‘Diphen’:ti,ab,kw OR ‘Diphenacen’:ti,ab,kw OR ‘Dobacen’:ti,ab,kw OR ‘Dormin’:ti,ab,kw OR ‘Dryhistan’:ti,ab,kw OR ‘Dytan’:ti,ab,kw OR ‘emesan’:ti,ab,kw OR ‘histergan’:ti,ab,kw OR ‘hydramine’:ti,ab,kw OR ‘hyrexin’:ti,ab,kw OR ‘ibiodral’:ti,ab,kw OR ‘medidryl’:ti,ab,kw OR ‘ethylamine’:ti,ab,kw OR ‘neosynodorm’:ti,ab,kw OR ‘nytol quickgels’:ti,ab,kw OR ‘o benzhydryldimethylaminoethanol’:ti,ab,kw OR ‘pm 255’:ti,ab,kw OR ‘q-dryl’:ti,ab,kw OR ‘reisegold’:ti,ab,kw OR ‘resmin’:ti,ab,kw OR ‘restamin’:ti,ab,kw OR ‘sediat’:ti,ab,kw OR ‘sedryl’:ti,ab,kw OR ‘silphen’:ti,ab,kw OR ‘sleepeze’:ti,ab,kw OR ‘sominex’:ti,ab,kw OR ‘syntedril’:ti,ab,kw OR ‘trux-adryl’:ti,ab,kw OR ‘tzoali’:ti,ab,kw OR ‘unisom sleepgels’:ti,ab,kw OR ‘valdrene’:ti,ab,kw OR ‘valu-dryl’:ti,ab,kw OR ‘venasmin’:ti,ab,kw OR ‘vertirosan’:ti,ab,kw OR ‘vicks formula 44’:ti,ab,kw OR ‘vilbin’:ti,ab,kw OR ‘wehdryl’:ti,ab,kw OR ‘ziradryl’:ti,ab,kw OR 'cetirizine'/exp OR 'cetirizine':ti,ab,kw OR 'acidrine':ti,ab,kw OR 'actifed allergie':ti,ab,kw OR 'adezio':ti,ab,kw OR 'agelmin':ti,ab,kw OR 'alairgix*':ti,ab,kw OR 'alercet':ti,ab,kw OR 'alerid':ti,ab,kw OR 'alerlisin':ti,ab,kw OR 'alertop':ti,ab,kw OR 'alerviden':ti,ab,kw OR 'aletir':ti,ab,kw OR 'alled':ti,ab,kw OR 'allertec':ti,ab,kw OR 'aller-tec':ti,ab,kw OR 'alltec':ti,ab,kw OR 'alzytec':ti,ab,kw OR 'benaday':ti,ab,kw OR 'Benadryl*':ti,ab,kw OR 'betarhin':ti,ab,kw OR 'cabal':ti,ab,kw OR 'cerazine':ti,ab,kw OR 'cerini':ti,ab,kw OR 'cerotec':ti,ab,kw OR 'cesta':ti,ab,kw OR 'cetalerg':ti,ab,kw OR 'ceterifug':ti,ab,kw OR 'cethis':ti,ab,kw OR 'cetiderm':ti,ab,kw OR 'cetidura':ti,ab,kw OR 'cetilich':ti,ab,kw OR 'cetil von ct':ti,ab,kw OR 'cetimin':ti,ab,kw OR 'cetin':ti,ab,kw OR 'ceti-puren':ti,ab,kw OR 'cetirax':ti,ab,kw OR 'cetirigamma':ti,ab,kw OR 'cetirin':ti,ab,kw OR 'cetirlan':ti,ab,kw OR 'ceti TAD':ti,ab,kw OR 'cetizin':ti,ab,kw OR 'cetrimed':ti,ab,kw OR 'cetrine':ti,ab,kw OR 'cetrizet':ti,ab,kw OR 'cetrizin':ti,ab,kw OR 'cetymin':ti,ab,kw OR 'cistamine':ti,ab,kw OR 'deallergy':ti,ab,kw OR 'drill allergie':ti,ab,kw OR 'falergi':ti,ab,kw OR 'finallerg':ti,ab,kw OR 'formistin':ti,ab,kw OR 'generit':ti,ab,kw OR 'histazine':ti,ab,kw OR 'histica':ti,ab,kw OR 'incidal-od':ti,ab,kw OR 'lergium':ti,ab,kw OR 'livoreactine':ti,ab,kw OR 'nosemin':ti,ab,kw OR 'nosmin':ti,ab,kw OR 'ozen':ti,ab,kw OR 'piriteze allergy':ti,ab,kw OR 'pollenase':ti,ab,kw OR 'pollenshield hayfever relief':ti,ab,kw OR 'prixlae':ti,ab,kw OR 'quzyttir':ti,ab,kw OR 'qzytir':ti,ab,kw OR 'raingen':ti,ab,kw OR 'razene':ti,ab,kw OR 'reactine':ti,ab,kw OR 'rhizin':ti,ab,kw OR 'risima':ti,ab,kw OR 'ritecam':ti,ab,kw OR 'ryvel':ti,ab,kw OR 'ryzen':ti,ab,kw OR 'sancotec':ti,ab,kw OR 'selitex':ti,ab,kw OR 'setizin':ti,ab,kw OR 'simtec':ti,ab,kw OR 'sutac':ti,ab,kw OR 'symitec':ti,ab,kw OR 'terizin':ti,ab,kw OR 'terzine':ti,ab,kw OR 'vick-zyrt':ti,ab,kw OR 'virlix':ti,ab,kw OR 'zenriz':ti,ab,kw OR 'zensil':ti,ab,kw OR 'zeran':ti,ab,kw OR 'zertine':ti,ab,kw OR 'zerviate':ti,ab,kw OR 'zetir':ti,ab,kw OR 'zicet':ti,ab,kw OR 'zinex':ti,ab,kw OR 'ziralton':ti,ab,kw OR 'zirtec':ti,ab,kw OR 'zirtek':ti,ab,kw OR 'zirtin':ti,ab,kw OR 'zyllergy':ti,ab,kw OR 'zymed':ti,ab,kw OR 'zyrac':ti,ab,kw OR 'zyrazine':ti,ab,kw OR 'zyrcon':ti,ab,kw OR 'zyrlex':ti,ab,kw OR 'zyrtec*':ti,ab,kw OR 'zyrtek':ti,ab,kw)

Calcium channel blockers

('calcium channel blocking agent'/exp OR 'calcium channel block*':ti,ab,kw OR 'calcium channel antagonist*':ti,ab,kw OR 'calcium channel inhibitor*':ti,ab,kw OR 'calcium inhibitor*':ti,ab,kw OR 'calcium antagonist*':ti,ab,kw OR 'calcium block*':ti,ab,kw OR 'calcium entry block*':ti,ab,kw OR 'amlodipine'/exp OR 'amlodipin*':ti,ab,kw OR 'Amloc':ti,ab,kw OR 'Amlodis':ti,ab,kw OR 'Amlopin':ti,ab,kw OR 'Amlor':ti,ab,kw OR 'Astudal':ti,ab,kw OR 'Istin':ti,ab,kw OR 'Norvasc':ti,ab,kw OR 'Stamlo':ti,ab,kw OR 'nifedipine'/exp OR 'nifedipine*':ti,ab,kw OR 'Adalat*':ti,ab,kw OR 'Adanif*':ti,ab,kw OR 'Adefin*':ti,ab,kw OR 'Adipine*':ti,ab,kw OR 'Afeditab*':ti,ab,kw OR 'Aldipin':ti,ab,kw OR 'Alonix-S':ti,ab,kw OR 'Alpha-nifedipine retard':ti,ab,kw OR 'Amarkor':ti,ab,kw OR 'Angibloc':ti,ab,kw OR 'Angipec':ti,ab,kw OR 'Antiblut':ti,ab,kw OR 'Apo-nifed':ti,ab,kw OR 'Aponifed':ti,ab,kw OR 'Aprical*':ti,ab,kw OR 'Atanaal softcap':ti,ab,kw OR 'Bay 1040':ti,ab,kw OR 'Bay1040':ti,ab,kw OR 'Bay a 1040':ti,ab,kw OR 'Bay a1040':ti,ab,kw OR 'Baya1040':ti,ab,kw OR 'Calcheck':ti,ab,kw OR 'Calcibloc*':ti,ab,kw OR 'Calcigard*':ti,ab,kw OR 'Calcilat':ti,ab,kw OR 'Calgina':ti,ab,kw OR 'Cardifen':ti,ab,kw OR 'Cardilat*':ti,ab,kw OR 'Cardionorm':ti,ab,kw OR 'Chronadalat*':ti,ab,kw OR 'Cipilat':ti,ab,kw OR 'Citilat':ti,ab,kw OR 'Coracten*':ti,ab,kw OR 'Cordafen':ti,ab,kw OR 'Cordaflex':ti,ab,kw OR 'Cordalat':ti,ab,kw OR 'Cordicant':ti,ab,kw OR 'Cordipen':ti,ab,kw OR 'Cordipin*':ti,ab,kw OR 'Corinfar':ti,ab,kw OR 'Korinfar':ti,ab,kw OR 'Coronpin':ti,ab,kw OR 'Corotrend':ti,ab,kw OR 'Denkifed':ti,ab,kw OR 'Depin':ti,ab,kw OR 'Dexipress*':ti,ab,kw OR 'Dignokonstant':ti,ab,kw OR 'Dilafed':ti,ab,kw OR 'Dipinkor':ti,ab,kw OR 'Duranifin':ti,ab,kw OR 'Ecodipin':ti,ab,kw OR 'Emaberin':ti,ab,kw OR 'Fedcor':ti,ab,kw OR 'Fedipin*':ti,ab,kw OR 'Fenamon*':ti,ab,kw OR 'Fenigidin':ti,ab,kw OR 'Fortipine':ti,ab,kw OR 'Glopir':ti,ab,kw OR 'Hadipine*':ti,ab,kw OR 'Herlat':ti,ab,kw OR 'Hexadilat':ti,ab,kw OR 'Hypan':ti,ab,kw OR 'Hypolar Retard':ti,ab,kw OR 'Infedipine':ti,ab,kw OR 'Jutadilat':ti,ab,kw OR 'Kemolat':ti,ab,kw OR 'Macorel':ti,ab,kw OR 'Megalat':ti,ab,kw OR 'Mifedipine':ti,ab,kw OR 'Moderat':ti,ab,kw OR 'Myogard':ti,ab,kw OR 'Nadipine':ti,ab,kw OR 'Nedipin':ti,ab,kw OR 'Nefedipine':ti,ab,kw OR 'Nelapine*':ti,ab,kw OR 'Neozipine*':ti,ab,kw OR 'Nidef':ti,ab,kw OR 'Nifangin':ti,ab,kw OR 'Nifar':ti,ab,kw OR 'Nifdemin':ti,ab,kw OR 'Nife-par':ti,ab,kw OR 'Nifebene':ti,ab,kw OR 'Nifecard':ti,ab,kw OR 'Nifecor':ti,ab,kw OR 'Nifedepat':ti,ab,kw OR 'Nifedicor*':ti,ab,kw OR 'Nifedilat':ti,ab,kw OR 'Nifedin*':ti,ab,kw OR 'Nifedipat':ti,ab,kw OR 'Nifedipin':ti,ab,kw OR 'Nifedipres*':ti,ab,kw OR 'Nifedirex lp':ti,ab,kw OR 'Nifehexal':ti,ab,kw OR 'Nifelat*':ti,ab,kw OR 'Nifensar*':ti,ab,kw OR 'Nifepidine*':ti,ab,kw OR 'Nifestad':ti,ab,kw OR 'Nifical':ti,ab,kw OR 'Nificard':ti,ab,kw OR 'Nifidine':ti,ab,kw OR 'Nifipen':ti,ab,kw OR 'Nifopress*':ti,ab,kw OR 'Nimodrel*':ti,ab,kw OR 'Nipin*':ti,ab,kw OR 'Normadil':ti,ab,kw OR 'Novo nifedin':ti,ab,kw OR 'Novonifedin':ti,ab,kw OR 'Nyefax*':ti,ab,kw OR 'Nypine':ti,ab,kw OR 'Odipin':ti,ab,kw OR 'Orix':ti,ab,kw OR 'Osmo-adalat':ti,ab,kw OR 'Phenygidine':ti,ab,kw OR 'Pidilat*':ti,ab,kw OR 'Procardia*':ti,ab,kw OR 'Rdd 1219':ti,ab,kw OR 'Rdd1219':ti,ab,kw OR 'Ronian':ti,ab,kw OR 'Sepamit':ti,ab,kw OR 'Slofedipine*':ti,ab,kw OR 'Tensipine*':ti,ab,kw OR 'Tibricol':ti,ab,kw OR 'Unidipine*':ti,ab,kw OR 'Valni*':ti,ab,kw OR 'Vascard':ti,ab,kw OR 'Vasdalat*':ti,ab,kw OR 'Zenusin':ti,ab,kw OR 'clevidipine'/exp OR 'clevidipine*':ti,ab,kw OR 'Clevelox':ti,ab,kw OR 'Cleviprex':ti,ab,kw OR 'nimodipine'/exp OR 'nimodipin*':ti,ab,kw OR 'Admon':ti,ab,kw OR 'Bay 9736':ti,ab,kw OR 'Bay9736':ti,ab,kw OR 'Bay e 9736':ti,ab,kw OR 'Bay e9736':ti,ab,kw OR 'Bay n5247':ti,ab,kw OR 'Bay n 5247':ti,ab,kw OR 'Bay n5248':ti,ab,kw OR 'Bay n 5248':ti,ab,kw OR 'Brainal':ti,ab,kw OR 'Calnit':ti,ab,kw OR 'Eugerial':ti,ab,kw OR 'Grifonimod':ti,ab,kw OR 'Kenesil':ti,ab,kw OR 'Kenzolol':ti,ab,kw OR 'Modus':ti,ab,kw OR 'Nidip':ti,ab,kw OR 'Nimodilat':ti,ab,kw OR 'Nimotop':ti,ab,kw OR 'Nisom':ti,ab,kw OR 'Nymalize':ti,ab,kw OR 'Periplum':ti,ab,kw OR 'Remontal':ti,ab,kw OR 'Tropocer':ti,ab,kw OR 'Vasoflex':ti,ab,kw OR 'Vasotop':ti,ab,kw OR 'nitrendipine'/exp OR 'nitrendipin*':ti,ab,kw OR 'Balminil':ti,ab,kw OR 'Bay e 5009':ti,ab,kw OR 'Bay e5009':ti,ab,kw OR 'Baylotensin':ti,ab,kw OR 'Bayotensin*':ti,ab,kw OR 'Baypresol':ti,ab,kw OR 'Baypress*':ti,ab,kw OR 'Gericin':ti,ab,kw OR 'Jutapress':ti,ab,kw OR 'Lusopress':ti,ab,kw OR 'Nitre AbZ':ti,ab,kw OR 'Nidrel':ti,ab,kw OR 'Nitregamma':ti,ab,kw OR 'Nitren 1A Pharma':ti,ab,kw OR 'Nitren Lich':ti,ab,kw OR 'Nitre-Puren':ti,ab,kw OR 'NitrePuren':ti,ab,kw OR 'Niprina':ti,ab,kw OR 'Nitrend KSK':ti,ab,kw OR 'Nitrendepat':ti,ab,kw OR 'Nitren acis':ti,ab,kw OR 'Nitrendi Biochemie':ti,ab,kw OR 'Nitrendidoc':ti,ab,kw OR 'Nitrendimerck':ti,ab,kw OR 'Nitrendimerck':ti,ab,kw OR 'Nitrendipine':ti,ab,kw OR 'Nitrensal':ti,ab,kw OR 'Nitrepress':ti,ab,kw OR 'Trendinol':ti,ab,kw OR 'Tensogradal':ti,ab,kw OR 'Vastensium':ti,ab,kw OR 'nicardipine'/exp OR 'nicardipine*':ti,ab,kw OR 'Angioglebil':ti,ab,kw OR 'Antagonil':ti,ab,kw OR 'Barizine':ti,ab,kw OR 'Bionicard':ti,ab,kw OR 'Cardene*':ti,ab,kw OR 'Cardepine*':ti,ab,kw OR 'Cardibloc':ti,ab,kw OR 'Cardipene':ti,ab,kw OR 'Dacarel':ti,ab,kw OR 'Dafil':ti,ab,kw OR 'Dagan':ti,ab,kw OR 'Flusemide':ti,ab,kw OR 'Karden':ti,ab,kw OR 'Lecibral':ti,ab,kw OR 'Lincil':ti,ab,kw OR 'Loxen':ti,ab,kw OR 'Lucenfal':ti,ab,kw OR 'Nerdipine':ti,ab,kw OR 'Nicaplant':ti,ab,kw OR 'Nicardal':ti,ab,kw OR 'Nicardil':ti,ab,kw OR 'Nicardipino*':ti,ab,kw OR 'Nicarpin':ti,ab,kw OR 'Nicodel':ti,ab,kw OR 'Nimicor':ti,ab,kw OR 'Perdipina':ti,ab,kw OR 'Perdipine*':ti,ab,kw OR 'Ranvil':ti,ab,kw OR 'Ridene':ti,ab,kw OR 'Roxen':ti,ab,kw OR 'rs 69216*':ti,ab,kw OR 'rs69216*':ti,ab,kw OR 'Rycarden':ti,ab,kw OR 'Rydene':ti,ab,kw OR 'Saf Card':ti,ab,kw OR 'SR Nicardipine':ti,ab,kw OR 'Vasodin':ti,ab,kw OR 'Vasonase':ti,ab,kw OR 'yc 93':ti,ab,kw OR 'yc93':ti,ab,kw OR 'y 93':ti,ab,kw OR 'y93':ti,ab,kw OR 'lercanidipine'/exp OR 'lercanidipine*':ti,ab,kw OR 'Atover':ti,ab,kw OR 'Brunoq':ti,ab,kw OR 'Carmen*':ti,ab,kw OR 'Corifeo':ti,ab,kw OR 'Coripren':ti,ab,kw OR 'Enalapril*':ti,ab,kw OR 'Karnidin':ti,ab,kw OR 'Ledip':ti,ab,kw OR 'Lercadip':ti,ab,kw OR 'Lercan':ti,ab,kw OR 'Lercaprel':ti,ab,kw OR 'Lercapress':ti,ab,kw OR 'Lercastad':ti,ab,kw OR 'Lerdip':ti,ab,kw OR 'Lerez':ti,ab,kw OR 'Lercatio':ti,ab,kw OR 'Lercaton':ti,ab,kw OR 'Lerka':ti,ab,kw OR 'Lerzam':ti,ab,kw OR 'Masnidipine':ti,ab,kw OR 'Primacor':ti,ab,kw OR 'REC 15-2375':ti,ab,kw OR 'REC 152375':ti,ab,kw OR 'REC15-2375':ti,ab,kw OR 'REC152375':ti,ab,kw OR 'TJN 324':ti,ab,kw OR 'TJN324':ti,ab,kw OR 'Vasodip':ti,ab,kw OR 'Zan-extra':ti,ab,kw OR 'Zaneril':ti,ab,kw OR 'Zanextra':ti,ab,kw OR 'Zanipress':ti,ab,kw OR 'Zanedip':ti,ab,kw OR 'Zanidip':ti,ab,kw OR 'Zanipril':ti,ab,kw OR 'Zanitek':ti,ab,kw OR 'felodipine'/exp OR 'felodipine':ti,ab,kw OR 'Agon*':ti,ab,kw OR 'Delofine*':ti,ab,kw OR 'Dilahex':ti,ab,kw OR 'Dilofen*':ti,ab,kw OR 'Dilopin':ti,ab,kw OR 'Fedil':ti,ab,kw OR 'Felim':ti,ab,kw OR 'Felo Biochemie':ti,ab,kw OR 'Felo ER':ti,ab,kw OR 'Felo-basf*':ti,ab,kw OR 'Felobal':ti,ab,kw OR 'Felobeta':ti,ab,kw OR 'Felocor*':ti,ab,kw OR 'Felodipin*':ti,ab,kw OR 'Felodur*':ti,ab,kw OR 'Felogamma*':ti,ab,kw OR 'Felogard':ti,ab,kw OR 'Felop':ti,ab,kw OR 'Felopine-sr':ti,ab,kw OR 'Felo-puren':ti,ab,kw OR 'Fensel':ti,ab,kw OR 'Flodil*':ti,ab,kw OR 'h 154-82':ti,ab,kw OR 'h 15482':ti,ab,kw OR 'h154-82':ti,ab,kw OR 'h15482':ti,ab,kw OR 'Hydac':ti,ab,kw OR 'Keydipin*':ti,ab,kw OR 'Lodistad*':ti,ab,kw OR 'Modip':ti,ab,kw OR 'Munobal*':ti,ab,kw OR 'Nirmadil':ti,ab,kw OR 'Parmid*':ti,ab,kw OR 'Penedil':ti,ab,kw OR 'Perfudal':ti,ab,kw OR 'Plendil*':ti,ab,kw OR 'Preslow':ti,ab,kw OR 'Prevex':ti,ab,kw OR 'Renedil':ti,ab,kw OR 'Selepine':ti,ab,kw OR 'Splendil*':ti,ab,kw OR 'Versant xr':ti,ab,kw OR 'diltiazem'/exp OR ‘diltiazem’:ti,ab,kw OR ‘acalix’:ti,ab,kw OR ‘adizem*’:ti,ab,kw OR ‘aldizem’:ti,ab,kw OR ‘altiazem*’:ti,ab,kw OR ‘anginyl’:ti,ab,kw OR ‘angiodrox’:ti,ab,kw OR ‘angiotrofen’:ti,ab,kw OR ‘angiotrofin*’:ti,ab,kw OR ‘angiozem’:ti,ab,kw OR ‘angitil’:ti,ab,kw OR ‘angizem’:ti,ab,kw OR ‘angoral’:ti,ab,kw OR ‘anoheal’:ti,ab,kw OR ‘anotrit’:ti,ab,kw OR ‘anzem’:ti,ab,kw OR ‘apo-diltiazem’:ti,ab,kw OR ‘auscard’:ti,ab,kw OR ‘balcor’:ti,ab,kw OR ‘beatizem’:ti,ab,kw OR ‘bi-tildiem*’:ti,ab,kw OR ‘blocalcin’:ti,ab,kw OR ‘bnp 32762’:ti,ab,kw OR ‘bnp32762’:ti,ab,kw OR ‘britiazim’:ti,ab,kw OR ‘bruzem’:ti,ab,kw OR ‘calcicard’:ti,ab,kw OR ‘calnurs’:ti,ab,kw OR ‘cardcal’:ti,ab,kw OR ‘cardiazem’:ti,ab,kw OR ‘cardiben s.r.’:ti,ab,kw OR ‘cardiem’:ti,ab,kw OR ‘cardil*’:ti,ab,kw OR ‘cardiosta*’:ti,ab,kw OR ‘cardium’:ti,ab,kw OR ‘Cardizem*’:ti,ab,kw OR ‘carex’:ti,ab,kw OR ‘carreldon*’:ti,ab,kw OR ‘cartia*’:ti,ab,kw OR ‘cascor*’:ti,ab,kw OR ‘cirilen*’:ti,ab,kw OR ‘cis diltiazem’:ti,ab,kw OR ‘coras’:ti,ab,kw OR ‘cordizem’:ti,ab,kw OR ‘crd 401’:ti,ab,kw OR ‘crd401’:ti,ab,kw OR ‘d2521’:ti,ab,kw OR ‘dazil’:ti,ab,kw OR ‘deltazen’:ti,ab,kw OR ‘diacor*’:ti,ab,kw OR ‘diatal’:ti,ab,kw OR ‘diazem*’:ti,ab,kw OR ‘dilacor*’:ti,ab,kw OR ‘diladel’:ti,ab,kw OR ‘dilatam*’:ti,ab,kw OR ‘dilcard*’:ti,ab,kw OR ‘dilem*’:ti,ab,kw OR ‘dilfar’:ti,ab,kw OR ‘dilgard’:ti,ab,kw OR ‘diloc’:ti,ab,kw OR ‘dilren*’:ti,ab,kw OR ‘dilso’:ti,ab,kw OR ‘dilt-cd’:ti,ab,kw OR ‘diltahexal’:ti,ab,kw OR ‘diltam’:ti,ab,kw OR ‘diltan*’:ti,ab,kw OR ‘diltelan’:ti,ab,kw OR ‘diltia*’:ti,ab,kw OR ‘diltime’:ti,ab,kw OR ‘diltiwas’:ti,ab,kw OR ‘diltstar’:ti,ab,kw OR ‘diltzac’:ti,ab,kw OR ‘dilzanton’:ti,ab,kw OR ‘dilzem*’:ti,ab,kw OR ‘dilzene’:ti,ab,kw OR ‘dilzereal*’:ti,ab,kw OR ‘dilzicardin’:ti,ab,kw OR ‘dinisor*’:ti,ab,kw OR ‘doclis*’:ti,ab,kw OR ‘dodexen*’:ti,ab,kw OR ‘dyalac’:ti,ab,kw OR ‘entrydil’:ti,ab,kw OR ‘etizem’:ti,ab,kw OR ‘filazem’:ti,ab,kw OR ‘gadoserin’:ti,ab,kw OR ‘grifodilzem’:ti,ab,kw OR ‘hagen’:ti,ab,kw OR ‘helsibon’:ti,ab,kw OR ‘herben’:ti,ab,kw OR ‘herbesser*’:ti,ab,kw OR ‘herbessor*’:ti,ab,kw OR ‘hesor’:ti,ab,kw OR ‘incoril*’:ti,ab,kw OR ‘iski-90’:ti,ab,kw OR ‘kaizem’:ti,ab,kw OR ‘kenzem’:ti,ab,kw OR ‘lacerol*’:ti,ab,kw OR ‘levodex’:ti,ab,kw OR ‘levozem’:ti,ab,kw OR ‘lytelsen’:ti,ab,kw OR ‘masdil*’:ti,ab,kw OR ‘miocardie’:ti,ab,kw OR ‘mk 793’:ti,ab,kw OR ‘mono-tildiem*’:ti,ab,kw OR ‘monotildiem’:ti,ab,kw OR ‘myonil*’:ti,ab,kw OR ‘pazeadin’:ti,ab,kw OR ‘presoken’:ti,ab,kw OR ‘slozem’:ti,ab,kw OR ‘slv 324’:ti,ab,kw OR ‘slv324’:ti,ab,kw OR ‘surazem’:ti,ab,kw OR ‘tazem’:ti,ab,kw OR ‘taztia*’:ti,ab,kw OR ‘tiadil’:ti,ab,kw OR ‘tiadylt’:ti,ab,kw OR ‘tiamate’:ti,ab,kw OR ‘tiazac’:ti,ab,kw OR ‘tilazem*’:ti,ab,kw OR ‘tildiem*’:ti,ab,kw OR ‘tilker’:ti,ab,kw OR ‘trans diltiazem’:ti,ab,kw OR ‘uni-masdil’:ti,ab,kw OR ‘vasmulax’:ti,ab,kw OR ‘vasocardol*’:ti,ab,kw OR ‘ven 307’:ti,ab,kw OR ‘ven307’:ti,ab,kw OR ‘wentizem*’:ti,ab,kw OR ‘zandil’:ti,ab,kw OR ‘zatim*’:ti,ab,kw OR ‘zemret*’:ti,ab,kw OR ‘zemtrial’:ti,ab,kw OR ‘zildem’:ti,ab,kw OR ‘ziruvate’:ti,ab,kw OR 'verapamil'/exp OR ‘verapamil’:ti,ab,kw OR ‘apo-verap’:ti,ab,kw OR ‘apoacor’:ti,ab,kw OR ‘arpamyl’:ti,ab,kw OR ‘azupamil’:ti,ab,kw OR ‘berkatens’:ti,ab,kw OR ‘calan*’:ti,ab,kw OR ‘calaptin*’:ti,ab,kw OR ‘cardiagutt’:ti,ab,kw OR ‘cardibeltin’:ti,ab,kw OR ‘cardiolen’:ti,ab,kw OR ‘cardiover’:ti,ab,kw OR ‘caveril’:ti,ab,kw OR ‘cintsu’:ti,ab,kw OR ‘civicor’:ti,ab,kw OR ‘coer 24’:ti,ab,kw OR ‘coraver’:ti,ab,kw OR ‘cordilat’:ti,ab,kw OR ‘cordilox*’:ti,ab,kw OR ‘corpamil’:ti,ab,kw OR ‘covera*’:ti,ab,kw OR ‘cp 16533 1’:ti,ab,kw OR ‘cp 16533-1’:ti,ab,kw OR ‘cp 165331’:ti,ab,kw OR ‘cp16533 1’:ti,ab,kw OR ‘cp16533-1’:ti,ab,kw OR ‘cp165331’:ti,ab,kw OR ‘d 365’:ti,ab,kw OR ‘d365’:ti,ab,kw OR ‘dignover’:ti,ab,kw OR ‘dilacoran*’:ti,ab,kw OR ‘dilacoron’:ti,ab,kw OR ‘durasoptin’:ti,ab,kw OR ‘falicard’:ti,ab,kw OR ‘finoptin’:ti,ab,kw OR ‘flamon’:ti,ab,kw OR ‘geangin’:ti,ab,kw OR ‘hexasoptin*’:ti,ab,kw OR ‘ikacor’:ti,ab,kw OR ‘ikakor’:ti,ab,kw OR ‘ikapress’:ti,ab,kw OR ‘iproveratril’:ti,ab,kw OR ‘iso-card*’:ti,ab,kw OR ‘isoptin*’:ti,ab,kw OR ‘levo verapamil’:ti,ab,kw OR ‘manidon*’:ti,ab,kw OR ‘napamil’:ti,ab,kw OR ‘novapamyl’:ti,ab,kw OR ‘novo-veramil’:ti,ab,kw OR ‘novopressan’:ti,ab,kw OR ‘phynoptin’:ti,ab,kw OR ‘quasar’:ti,ab,kw OR ‘ravamil*’:ti,ab,kw OR ‘securon*’:ti,ab,kw OR ‘univer’:ti,ab,kw OR ‘vasolan’:ti,ab,kw OR ‘vasomil’:ti,ab,kw OR ‘vasopten’:ti,ab,kw OR ‘verabeta’:ti,ab,kw OR ‘veracaps*’:ti,ab,kw OR ‘veracor’:ti,ab,kw OR ‘verahexal’:ti,ab,kw OR ‘veraloc’:ti,ab,kw OR ‘veramex*’:ti,ab,kw OR ‘veramil’:ti,ab,kw OR ‘verapamil*’:ti,ab,kw OR ‘verapin’:ti,ab,kw OR ‘verapress*’:ti,ab,kw OR ‘veratad’:ti,ab,kw OR ‘verdilac’:ti,ab,kw OR ‘verelan*’:ti,ab,kw OR ‘veroptin stada’:ti,ab,kw OR ‘verpamil’:ti,ab,kw OR ‘vetrimil’:ti,ab,kw OR ‘vortac’:ti,ab,kw OR ‘zolvera’:ti,ab,kw)

Antimalarials

('antimalarial agent'/exp OR 'antimalarial*':ti,ab,kw OR 'anti-malarial*':ti,ab,kw OR 'artemisinin*':ti,ab,kw OR 'arteannuin':ti,ab,kw OR 'qinghaosu':ti,ab,kw OR 'quinghaosu':ti,ab,kw OR 'huanghuahaosu':ti,ab,kw OR 'qing hao su':ti,ab,kw OR 'quing hau sau':ti,ab,kw OR 'sesquiterpene'/exp OR 'artemether':ti,ab,kw OR 'artesunate':ti,ab,kw OR 'chloroquine*':ti,ab,kw OR 'chlorochin':ti,ab,kw OR 'Chingamin':ti,ab,kw OR 'Khingamin':ti,ab,kw OR 'Nivaquine':ti,ab,kw OR 'Aralen':ti,ab,kw OR 'Arequin':ti,ab,kw OR 'Arechine':ti,ab,kw OR 'hydroxychloroquine*':ti,ab,kw OR 'hydroxycloroquine*':ti,ab,kw OR 'hydrochloroquine*':ti,ab,kw OR 'hydrocloroquine*':ti,ab,kw OR 'oxychloroquine*':ti,ab,kw OR 'oxycloroquine*':ti,ab,kw OR 'hydroxychlorochin':ti,ab,kw OR 'oxychlorochin':ti,ab,kw OR 'oxychloroquine':ti,ab,kw OR 'Plaquenil':ti,ab,kw OR 'chloroquinol':ti,ab,kw OR 'ercoquin':ti,ab,kw OR 'pyrimethamin*':ti,ab,kw OR 'pirimethamin*':ti,ab,kw OR 'primethamine':ti,ab,kw OR 'Malocide':ti,ab,kw OR 'Tindurine':ti,ab,kw OR 'Chloridin*':ti,ab,kw OR 'Daraprim':ti,ab,kw OR 'Darapram':ti,ab,kw OR 'Darapraim':ti,ab,kw OR 'Malocide':ti,ab,kw OR 'tindurin':ti,ab,kw OR 'proguanil'/exp OR 'proguanil*':ti,ab,kw OR 'Balusil':ti,ab,kw OR 'Bigumal':ti,ab,kw OR 'Chlorguanid*':ti,ab,kw OR 'Chloriguane*':ti,ab,kw OR 'Chlorguanil':ti,ab,kw OR 'Chloroguanid*':ti,ab,kw OR 'Cloroguanid*':ti,ab,kw OR 'Diguanil':ti,ab,kw OR 'Diguanyl':ti,ab,kw OR 'Drinupal':ti,ab,kw OR 'Guanatol':ti,ab,kw OR 'Paguinal':ti,ab,kw OR 'Paludrin*':ti,ab,kw OR 'Palusil':ti,ab,kw OR 'Plasin':ti,ab,kw OR 'Proguanide':ti,ab,kw OR 'Proquanil':ti,ab,kw OR 'Tirian':ti,ab,kw OR 'sulfadoxine'/exp OR 'sulfadoxin*':ti,ab,kw OR 'Fanasil':ti,ab,kw OR 'Fanzil':ti,ab,kw OR 'Sanasil':ti,ab,kw 'sulfaorthodimethoxin*':ti,ab,kw OR 'sulfonmetoxin*':ti,ab,kw OR 'sulfonmethoxin*':ti,ab,kw OR 'sulformetoxin*':ti,ab,kw OR 'sulformethoxin*':ti,ab,kw OR 'sulfon metoxin*':ti,ab,kw OR 'sulfon methoxin*':ti,ab,kw OR 'sulfor metoxin*':ti,ab,kw OR 'sulfor methoxin*':ti,ab,kw OR 'sulforthomidin*':ti,ab,kw OR 'sulforthodimethoxin*':ti,ab,kw OR 'sulfurmetoxin*':ti,ab,kw OR 'sulphadoxin*':ti,ab,kw OR 'sulphormethoxin*':ti,ab,kw OR 'sulphor methoxin*':ti,ab,kw OR 'sulphormetoxin*':ti,ab,kw OR 'sulphor metoxin*':ti,ab,kw OR 'dapsone'/exp OR 'dapson*':ti,ab,kw OR 'Aczone':ti,ab,kw OR 'Atrisone':ti,ab,kw OR 'Avlosulfan':ti,ab,kw OR 'Avlosulfon*':ti,ab,kw OR 'Croysulfone*':ti,ab,kw OR 'Dapsoderm-X':ti,ab,kw OR 'DADPS':ti,ab,kw OR 'DDS':ti,ab,kw OR 'diamino diphenyl sulfone':ti,ab,kw OR 'diaminodiphenyl sulfone':ti,ab,kw OR 'diaminodiphenylsulfon*':ti,ab,kw OR 'diammodiphenylsulfon*':ti,ab,kw OR 'diaphenylsulfon*':ti,ab,kw OR 'diaphenyl sulfon*':ti,ab,kw OR 'diaphenylsulphon*':ti,ab,kw OR 'diaphenyl sulphon*':ti,ab,kw OR 'Disulone':ti,ab,kw OR 'Diphenason*':ti,ab,kw OR 'Diphone':ti,ab,kw OR 'Dopsan':ti,ab,kw OR 'Dumitone':ti,ab,kw OR 'Eporal':ti,ab,kw OR 'Lepravir':ti,ab,kw OR 'Novasulfon':ti,ab,kw OR 'Novophone':ti,ab,kw OR 'sulfonyldianiline':ti,ab,kw OR 'Servidapson*':ti,ab,kw OR 'Sulfadione':ti,ab,kw OR 'Sulfadoine':ti,ab,kw OR 'Sulfona*':ti,ab,kw OR 'Sulfone':ti,ab,kw OR 'Udolac':ti,ab,kw)

Other medications

*Antibiotics*

('antiinfective agent'/exp OR 'anti-infective*':ti,ab,kw OR 'antiinfective*':ti,ab,kw OR 'anti-bacterial*':ti,ab,kw OR 'antibacterial*':ti,ab,kw OR 'anti-myobacterial*':ti,ab,kw OR 'antimyobacterial*':ti,ab,kw OR 'bacteriocid*':ti,ab,kw OR 'antibiotic*':ti,ab,kw OR 'antimicrobial*':ti,ab,kw OR 'antiseptic*':ti,ab,kw OR 'chemotherapeutic agent':ti,ab,kw OR 'chemotherapeutic drug':ti,ab,kw OR 'chemotherapeutica':ti,ab,kw OR 'microbiological agent':ti,ab,kw OR 'cefalexin'/exp OR 'cephalexin*':ti,ab,kw OR 'cefalexin':ti,ab,kw OR 'Ceporexine':ti,ab,kw OR 'Palitrex':ti,ab,kw OR 'cefixime'/exp OR 'cefixime*':ti,ab,kw OR 'Suprax':ti,ab,kw OR 'cefuroxime'/exp OR 'cefuroxime':ti,ab,kw OR 'cephuroxime':ti,ab,kw OR 'Zinacef':ti,ab,kw OR 'Ketocef':ti,ab,kw OR 'ciprofloxacin'/exp OR 'cipro*':ti,ab,kw OR 'Ciprinol':ti,ab,kw OR 'fleroxacin':ti,ab,kw OR 'Quinodis':ti,ab,kw OR 'erythromycin'/exp OR 'erythromycin*':ti,ab,kw OR 'T-Stat':ti,ab,kw OR 'TStat':ti,ab,kw OR 'Erymax':ti,ab,kw OR 'Erycette':ti,ab,kw OR 'Ilotycin':ti,ab,kw OR 'gemifloxacin'/exp OR 'gemifloxacin*':ti,ab,kw OR 'Factive':ti,ab,kw OR 'levofloxacin'/exp OR 'levofloxacin*':ti,ab,kw OR 'Quixin':ti,ab,kw OR 'Levaquin':ti,ab,kw OR 'metronidazole'/exp OR 'metronidazole*':ti,ab,kw OR 'Satric':ti,ab,kw OR 'Trichazol':ti,ab,kw OR 'Trichopol':ti,ab,kw OR 'Trivazol':ti,ab,kw OR 'Vagilen':ti,ab,kw OR 'Bayer 5360':ti,ab,kw OR 'Danizol':ti,ab,kw OR 'Flagy':ti,ab,kw OR 'Gineflavir':ti,ab,kw OR 'Metric':ti,ab,kw OR 'Metrodzhil':ti,ab,kw OR 'MetroGel':ti,ab,kw OR 'Metrogyl':ti,ab,kw OR 'Clont':ti,ab,kw OR 'spiramycin'/exp OR 'spiramycin*':ti,ab,kw OR 'Rovamycine':ti,ab,kw OR 'Selectomycin':ti,ab,kw OR 'Rovamycin':ti,ab,kw)

*Antivirals*

('antivirus agent'/exp OR 'antivirus*':ti,ab,kw OR 'antiviral*':ti,ab,kw OR 'anti viral*':ti,ab,kw OR 'virucidal*':ti,ab,kw OR 'viral inhibitor*':ti,ab,kw OR 'virus repressor*':ti,ab,kw OR 'virostatic*':ti,ab,kw OR 'virustatic*':ti,ab,kw OR 'virucide*':ti,ab,kw OR 'foscarnet'/exp OR 'foscarnet':ti,ab,kw OR 'phosphonoform*':ti,ab,kw OR 'foscavir':ti,ab,kw)

*Anesthetics*

('anesthetic agent'/exp OR 'anesthetic*':ti,ab,kw OR 'anaesthetic*':ti,ab,kw OR 'preanaesthetic*':ti,ab,kw OR 'preanesthetic*':ti,ab,kw OR 'nitric oxide'/exp OR 'nitric oxide':ti,ab,kw OR 'nitrogen monoxide':ti,ab,kw OR 'mononitrogen monoxide':ti,ab,kw OR 'genosyl':ti,ab,kw OR 'inomax':ti,ab,kw OR 'noxivent':ti,ab,kw OR 'fentanyl'/exp OR 'fentanyl*':ti,ab,kw OR 'phentanyl*':ti,ab,kw OR 'defent':ti,ab,kw OR 'duragesic*':ti,ab,kw OR 'durogesic':ti,ab,kw OR 'durotep':ti,ab,kw OR 'epufen':ti,ab,kw OR 'fentalis':ti,ab,kw OR 'fentamat':ti,ab,kw OR 'fentamyl':ti,ab,kw OR 'fentanest':ti,ab,kw OR 'fentanex':ti,ab,kw OR 'fentanil':ti,ab,kw OR 'fentora':ti,ab,kw OR 'fetanex':ti,ab,kw OR 'fetnanyl':ti,ab,kw OR 'leptanal':ti,ab,kw OR 'matrifen':ti,ab,kw OR 'mezolar matrix':ti,ab,kw OR 'pecfent':ti,ab,kw OR 'rapinyl':ti,ab,kw OR 'recuvyra':ti,ab,kw OR 'sublimaze':ti,ab,kw OR 'subsys':ti,ab,kw OR 'tanyl':ti,ab,kw OR 'tilotrans':ti,ab,kw OR 'transfenta':ti,ab,kw OR 'wafernyl':ti,ab,kw OR 'morphine'/exp OR 'morphine*':ti,ab,kw OR 'MS Contin':ti,ab,kw OR 'Oramorph SR':ti,ab,kw OR 'Duramorph':ti,ab,kw OR 'anpec':ti,ab,kw OR 'duromorph':ti,ab,kw OR 'epimorph':ti,ab,kw OR 'miro':ti,ab,kw OR 'morfin':ti,ab,kw OR 'morfine':ti,ab,kw OR 'morphia':ti,ab,kw OR 'morphin':ti,ab,kw OR 'morphinium':ti,ab,kw OR 'morphium':ti,ab,kw OR 'opso':ti,ab,kw OR 'skenan':ti,ab,kw OR 'propofol'/exp OR 'propofol':ti,ab,kw OR 'anepol':ti,ab,kw OR 'anesia':ti,ab,kw OR 'aquafol':ti,ab,kw OR 'cryotol':ti,ab,kw OR 'diisoprofol':ti,ab,kw OR 'diprivan':ti,ab,kw OR 'diprofol':ti,ab,kw OR 'disoprivan':ti,ab,kw OR 'disoprofol':ti,ab,kw OR 'fresofol':ti,ab,kw OR 'gobbifol':ti,ab,kw OR 'hiremon':ti,ab,kw OR 'ivofol':ti,ab,kw OR 'plofed':ti,ab,kw OR 'pofol':ti,ab,kw OR 'profast':ti,ab,kw OR 'propocam':ti,ab,kw OR 'propolipid':ti,ab,kw OR 'propoven':ti,ab,kw OR 'provive':ti,ab,kw OR 'rapinovet':ti,ab,kw OR 'rapiva':ti,ab,kw OR 'recofol*':ti,ab,kw OR 'ripol':ti,ab,kw OR 'safol':ti,ab,kw OR 'spifol':ti,ab,kw OR 'spiva':ti,ab,kw OR 'unifol':ti,ab,kw OR 'sevoflurane'/exp OR 'sevofluran*':ti,ab,kw OR 'petrem':ti,ab,kw OR 'sevo-anesteran':ti,ab,kw OR 'sevocalm':ti,ab,kw OR 'sevoflo':ti,ab,kw OR 'sevofrane':ti,ab,kw OR 'sevohale':ti,ab,kw OR 'sevorane':ti,ab,kw OR 'sevotec':ti,ab,kw OR 'sojourn':ti,ab,kw OR 'ultane*':ti,ab,kw)

*Analgesics & antipyretics*

('analgesic agent'/exp OR 'analgesic*':ti,ab,kw OR 'analgetic*':ti,ab,kw OR 'antalgic*':ti,ab,kw OR 'anodyne*':ti,ab,kw OR 'antinociceptive*':ti,ab,kw OR 'antipyretic agent'/exp OR 'antipyretic*':ti,ab,kw OR 'antifebrile*':ti,ab,kw OR 'paracetamol'/exp OR 'paracetamol':ti,ab,kw OR 'acetaminophen*':ti,ab,kw OR 'hydroxyacetanilide':ti,ab,kw OR 'Acetamidophenol':ti,ab,kw OR 'Acephen':ti,ab,kw OR 'Acetaco':ti,ab,kw OR 'Tylenol':ti,ab,kw OR 'Anacin*':ti,ab,kw OR 'Datril':ti,ab,kw OR 'Panadol':ti,ab,kw OR 'Acamol':ti,ab,kw OR 'Algotropyl':ti,ab,kw OR 'pentazocine'/exp OR 'pentazocin*':ti,ab,kw OR 'pentozocin*':ti,ab,kw OR 'pentacozin*':ti,ab,kw OR 'dolapent':ti,ab,kw OR 'fortal':ti,ab,kw OR 'fortalgesic':ti,ab,kw OR 'fortral*':ti,ab,kw OR 'fortwin':ti,ab,kw OR 'lexir':ti,ab,kw OR 'liticon':ti,ab,kw OR 'peltazon':ti,ab,kw OR 'pentafen':ti,ab,kw OR 'pentagin':ti,ab,kw OR 'pentalgina':ti,ab,kw OR 'perutagin':ti,ab,kw OR 'sosegon':ti,ab,kw OR 'sosigon':ti,ab,kw OR 'talioin':ti,ab,kw OR 'talwin':ti,ab,kw)

*Antitussives*

('antitussive agent'/exp OR 'antitussive*':ti,ab,kw OR 'cough suppressant*':ti,ab,kw OR 'cough depressant*':ti,ab,kw OR 'anticough*':ti,ab,kw OR 'butamirate'/exp OR 'butamirate*':ti,ab,kw OR 'butamyrate*':ti,ab,kw OR 'Sinecod':ti,ab,kw OR 'cloperastine'/exp 'cloperastine*':ti,ab,kw OR 'codeine'/exp OR 'codein*':ti,ab,kw OR 'Ardinex':ti,ab,kw OR 'Tussicalm':ti,ab,kw OR 'Pentuss':ti,ab,kw OR 'dextromethorphan'/exp OR 'dextromethorphan*':ti,ab,kw OR 'methorphan*':ti,ab,kw OR 'racemethorphan*':ti,ab,kw OR 'Delsym':ti,ab,kw)

*BZD*

('bromazepam'/exp OR 'bromazepam':ti,ab,kw OR 'anyrex':ti,ab,kw OR 'bartul':ti,ab,kw OR 'bromalich':ti,ab,kw OR 'bromaz 1A pharma':ti,ab,kw OR 'bromazanil':ti,ab,kw OR 'bromazep Von Ct':ti,ab,kw OR 'compedium':ti,ab,kw OR 'creosedi':ti,ab,kw OR 'durazanil':ti,ab,kw OR 'lectopam':ti,ab,kw OR 'lekotam':ti,ab,kw OR 'lexamil':ti,ab,kw OR 'lexatin':ti,ab,kw OR 'lexaurin':ti,ab,kw OR 'lexilium':ti,ab,kw OR 'lexomil':ti,ab,kw OR 'lexotan*':ti,ab,kw OR 'normoc':ti,ab,kw OR 'sintrogel':ti,ab,kw OR 'brotizolam'/exp OR 'brotizolam':ti,ab,kw OR 'dormex':ti,ab,kw OR 'ladormin':ti,ab,kw OR 'lendorm':ti,ab,kw OR 'lendormin':ti,ab,kw OR 'lindormin':ti,ab,kw OR 'mederantil':ti,ab,kw OR 'noctilan':ti,ab,kw OR 'sintonal':ti,ab,kw OR 'zolpidem'/exp OR 'zolpidem*':ti,ab,kw OR 'Zolirin':ti,ab,kw OR 'Zolpi-Lich':ti,ab,kw OR 'Zolpinox':ti,ab,kw OR 'Zolpimist':ti,ab,kw OR 'Ambien':ti,ab,kw OR 'Amsic':ti,ab,kw OR 'Bikalm':ti,ab,kw OR 'Stilnoct':ti,ab,kw OR 'Stilnox':ti,ab,kw OR 'Dalparan':ti,ab,kw OR 'Zodormdura':ti,ab,kw OR 'Zoldem':ti,ab,kw)

*Retonoids*

('retinoid'/exp OR 'retinoid*':ti,ab,kw OR 'isotretinoin'/exp OR 'isotretinoin':ti,ab,kw OR 'a cnotren':ti,ab,kw OR 'a-cnotren':ti,ab,kw OR 'abosorica':ti,ab,kw OR 'absorica*':ti,ab,kw OR 'accuran':ti,ab,kw OR 'accure':ti,ab,kw OR 'accutane*':ti,ab,kw OR 'accutin':ti,ab,kw OR 'acnal sc':ti,ab,kw OR 'acnemin':ti,ab,kw OR 'acnenor':ti,ab,kw OR 'acnetrait':ti,ab,kw OR 'acnetrex':ti,ab,kw OR 'acnogen':ti,ab,kw OR 'acnotin':ti,ab,kw OR 'acnotren':ti,ab,kw OR 'actaven':ti,ab,kw OR 'aisoskin':ti,ab,kw OR 'akinol':ti,ab,kw OR 'aknenormin':ti,ab,kw OR 'amnesteem':ti,ab,kw OR 'axotret':ti,ab,kw OR 'ciscumed':ti,ab,kw OR 'ciscutan':ti,ab,kw OR 'claravis':ti,ab,kw OR 'contracne':ti,ab,kw OR 'curacne*':ti,ab,kw OR 'curatane':ti,ab,kw OR 'decutan':ti,ab,kw OR 'dercutane':ti,ab,kw OR 'flexresan':ti,ab,kw OR 'inerta':ti,ab,kw OR 'isdiben':ti,ab,kw OR 'iso tretinoin':ti,ab,kw OR 'isoacne':ti,ab,kw OR 'isocural':ti,ab,kw OR 'isoderm':ti,ab,kw OR 'isogalen':ti,ab,kw OR 'isomacne':ti,ab,kw OR 'isoretinoic acid':ti,ab,kw OR 'isoretinoin':ti,ab,kw OR 'isoriac':ti,ab,kw OR 'isosupra lidose':ti,ab,kw OR 'isotane':ti,ab,kw OR 'isotren':ti,ab,kw OR 'isotret':ti,ab,kw OR 'isotret-hexal':ti,ab,kw OR 'isotrex*':ti,ab,kw OR 'isotroin':ti,ab,kw OR 'izotek':ti,ab,kw OR 'izotziaja':ti,ab,kw OR 'mayesta':ti,ab,kw OR 'medinac':ti,ab,kw OR 'myorisan':ti,ab,kw OR 'newtinon sc':ti,ab,kw OR 'nimegen':ti,ab,kw OR 'oratane':ti,ab,kw OR 'pharmiso':ti,ab,kw OR 'pinple':ti,ab,kw OR 'procuta':ti,ab,kw OR 'procuta ge':ti,ab,kw OR 'reducar':ti,ab,kw OR 'reticutan':ti,ab,kw OR 'retinoin, iso':ti,ab,kw OR 'rizuderm':ti,ab,kw OR 'roaccutan*':ti,ab,kw OR 'roaccuttan':ti,ab,kw OR 'roacnetan':ti,ab,kw OR 'roacutan':ti,ab,kw OR 'roacuttan':ti,ab,kw OR 'rocne':ti,ab,kw OR 'rotaxin':ti,ab,kw OR 'rotren':ti,ab,kw OR 'sotret':ti,ab,kw OR 'stiefotrex':ti,ab,kw OR 'teriosal':ti,ab,kw OR 'tretin':ti,ab,kw OR 'tretinex':ti,ab,kw OR 'tretiosan':ti,ab,kw OR 'tretoskin':ti,ab,kw OR 'zenatane':ti,ab,kw)

*Anthelminthics*

('anthelmintic agent'/exp OR 'anthelmintic*':ti,ab,kw OR 'anthelminthic*':ti,ab,kw OR 'antihelmintic*':ti,ab,kw OR 'antihelminthic*':ti,ab,kw OR 'anti-helmintic*':ti,ab,kw OR 'anti-helminthic*':ti,ab,kw OR 'helminthicidal*':ti,ab,kw OR 'vermicid*':ti,ab,kw OR 'vermifuge*':ti,ab,kw OR 'albendazole'/exp OR 'albendazol*':ti,ab,kw OR 'abentel':ti,ab,kw OR 'adazol':ti,ab,kw OR 'albatel':ti,ab,kw OR 'alben':ti,ab,kw OR 'albendazol':ti,ab,kw OR 'albendol':ti,ab,kw OR 'albendoral':ti,ab,kw OR 'albenza':ti,ab,kw OR 'albenzol':ti,ab,kw OR 'albex':ti,ab,kw OR 'albezole':ti,ab,kw OR 'alfuca':ti,ab,kw OR 'alminth':ti,ab,kw OR 'alzental':ti,ab,kw OR 'alzol':ti,ab,kw OR 'anthelmex':ti,ab,kw OR 'bendapar':ti,ab,kw OR 'bendex-400':ti,ab,kw OR 'bilutac':ti,ab,kw OR 'borotel':ti,ab,kw OR 'ceprazol':ti,ab,kw OR 'champs d-worm 6':ti,ab,kw OR 'ciclopar':ti,ab,kw OR 'digezanol':ti,ab,kw OR 'disthelm':ti,ab,kw OR 'endoplus':ti,ab,kw OR 'emanthal':ti,ab,kw OR 'eskazole':ti,ab,kw OR 'fintel':ti,ab,kw OR 'gascop':ti,ab,kw OR 'getzol':ti,ab,kw OR 'helben':ti,ab,kw OR 'helmiben':ti,ab,kw OR 'helmindazol':ti,ab,kw OR 'kalbend':ti,ab,kw OR 'labenda':ti,ab,kw OR 'loveral':ti,ab,kw OR 'lurdex':ti,ab,kw OR 'mebenix':ti,ab,kw OR 'metiazol':ti,ab,kw OR 'mycotel':ti,ab,kw OR 'nemozole':ti,ab,kw OR 'pantex':ti,ab,kw OR 'paranthil':ti,ab,kw OR 'proftril*':ti,ab,kw OR 'rotopar':ti,ab,kw OR 'rozolex':ti,ab,kw OR 'sioban':ti,ab,kw OR 'valbantel':ti,ab,kw OR 'valbazen':ti,ab,kw OR 'valbenzen':ti,ab,kw OR 'vastus':ti,ab,kw OR 'vemizol':ti,ab,kw OR 'vermin plus':ti,ab,kw OR 'xadem':ti,ab,kw OR 'zeben':ti,ab,kw OR 'zela':ti,ab,kw OR 'zentab':ti,ab,kw OR 'zentel':ti,ab,kw)

*Cytostatics*

('cytostatic agent'/exp OR 'cytostatic*':ti,ab,kw OR 'capecitabine'/exp OR 'capecitabine':ti,ab,kw OR 'apecitab':ti,ab,kw OR 'atubri':ti,ab,kw OR 'cacit':ti,ab,kw OR 'capcel':ti,ab,kw OR 'capebina':ti,ab,kw OR 'capecel':ti,ab,kw OR 'capecitalox':ti,ab,kw OR 'capecite':ti,ab,kw OR 'capegard':ti,ab,kw OR 'capezam':ti,ab,kw OR 'capibine':ti,ab,kw OR 'capicet':ti,ab,kw OR 'capiibine':ti,ab,kw OR 'capnat':ti,ab,kw OR 'capoda':ti,ab,kw OR 'capostat':ti,ab,kw OR 'capsy':ti,ab,kw OR 'capxcel':ti,ab,kw OR 'caxeta':ti,ab,kw OR 'cerex':ti,ab,kw OR 'citabin':ti,ab,kw OR 'coloxet':ti,ab,kw OR 'ecansya':ti,ab,kw OR 'kapetral':ti,ab,kw OR 'naprocap':ti,ab,kw OR 'preveloda':ti,ab,kw OR 'xabine':ti,ab,kw OR 'xalvobin':ti,ab,kw OR 'xecap':ti,ab,kw OR 'xelazor':ti,ab,kw OR 'xelcip':ti,ab,kw OR 'xelocel':ti,ab,kw OR 'xeloda':ti,ab,kw OR 'zerectum':ti,ab,kw OR 'zocitab':ti,ab,kw)

*Cholinesterase inhibitors*

('galantamine'/exp OR 'galantamin*':ti,ab,kw OR 'galanthamin*':ti,ab,kw OR 'acumor*':ti,ab,kw OR 'alenzo':ti,ab,kw OR 'aneprosil':ti,ab,kw OR 'bergal*':ti,ab,kw OR 'consion*':ti,ab,kw OR 'elmino*':ti,ab,kw OR 'gaalin':ti,ab,kw OR 'galanaxiro':ti,ab,kw OR 'galantex':ti,ab,kw OR 'galanthen':ti,ab,kw OR 'galanyl':ti,ab,kw OR 'galatamin*':ti,ab,kw OR 'galema*':ti,ab,kw OR 'galnora':ti,ab,kw OR 'galsya*':ti,ab,kw OR 'galzemic*':ti,ab,kw OR 'gamyl':ti,ab,kw OR 'gatalin*':ti,ab,kw OR 'gazylan*':ti,ab,kw OR 'girlamen':ti,ab,kw OR 'jilkon':ti,ab,kw OR 'lotprosin*':ti,ab,kw OR 'loxifren':ti,ab,kw OR 'luventa*':ti,ab,kw OR 'lycoremin*':ti,ab,kw OR 'margal':ti,ab,kw OR 'masparen*':ti,ab,kw OR 'memo (drug) ':ti,ab,kw OR 'memoton life':ti,ab,kw OR 'memoton-life':ti,ab,kw OR 'micol (galantamine) ':ti,ab,kw OR 'natagal*':ti,ab,kw OR 'nivalin*':ti,ab,kw OR 'razadyne*':ti,ab,kw OR 'reminyl*':ti,ab,kw OR 'spegal*':ti,ab,kw OR 'tamilin':ti,ab,kw OR 'vertusal*':ti,ab,kw OR 'zentan':ti,ab,kw OR 'zoroflog':ti,ab,kw OR 'rivastigmine'/exp OR 'rivastigmin*':ti,ab,kw OR 'alzest':ti,ab,kw OR 'exelon*':ti,ab,kw OR 'kingsmin':ti,ab,kw OR 'nimvastid':ti,ab,kw OR 'prometax':ti,ab,kw OR 'rivastach':ti,ab,kw OR 'donepezil'/exp OR 'donepezil*':ti,ab,kw OR 'adlarity':ti,ab,kw OR 'adonep':ti,ab,kw OR 'adtreat':ti,ab,kw OR 'alertan':ti,ab,kw OR 'alzancer':ti,ab,kw OR 'alzepezil':ti,ab,kw OR 'alzepil':ti,ab,kw OR 'alzil':ti,ab,kw OR 'apo-doperil':ti,ab,kw OR 'aricept*':ti,ab,kw OR 'aripezil':ti,ab,kw OR 'aripil':ti,ab,kw OR 'asenta':ti,ab,kw OR 'blixie':ti,ab,kw OR 'caricia':ti,ab,kw OR 'cenipil':ti,ab,kw OR 'ciclodin':ti,ab,kw OR 'cogiton*':ti,ab,kw OR 'covolos':ti,ab,kw OR 'darezil':ti,ab,kw OR 'darizol':ti,ab,kw OR 'davia':ti,ab,kw OR 'demelan':ti,ab,kw OR 'dementis':ti,ab,kw OR 'demezilon':ti,ab,kw OR 'destezil':ti,ab,kw OR 'dezial':ti,ab,kw OR 'donebrain':ti,ab,kw OR 'donecept':ti,ab,kw OR 'donectil*':ti,ab,kw OR 'donefien':ti,ab,kw OR 'donegal':ti,ab,kw OR 'doneliquid geriasan':ti,ab,kw OR 'donepesan':ti,ab,kw OR 'donepestan':ti,ab,kw OR 'donepex':ti,ab,kw OR 'doneprion':ti,ab,kw OR 'donerion':ti,ab,kw OR 'donestad':ti,ab,kw OR 'donester':ti,ab,kw OR 'donesyn':ti,ab,kw OR 'donpethon':ti,ab,kw OR 'dospelin':ti,ab,kw OR 'dozept':ti,ab,kw OR 'dozilax':ti,ab,kw OR 'eranz':ti,ab,kw OR 'filosept':ti,ab,kw OR 'hania':ti,ab,kw OR 'kognezil':ti,ab,kw OR 'landex':ti,ab,kw OR 'lixben':ti,ab,kw OR 'lizidra':ti,ab,kw OR 'medozil':ti,ab,kw OR 'memac':ti,ab,kw OR 'memorion':ti,ab,kw OR 'memorit':ti,ab,kw OR 'navazil':ti,ab,kw OR 'nepanizil':ti,ab,kw OR 'neperion':ti,ab,kw OR 'nepezil':ti,ab,kw OR 'nepla':ti,ab,kw OR 'oromentis':ti,ab,kw OR 'palixid':ti,ab,kw OR 'pamigen':ti,ab,kw OR 'penezil':ti,ab,kw OR 'pezale':ti,ab,kw OR 'peziled':ti,ab,kw OR 'pezimax':ti,ab,kw OR 'promemore':ti,ab,kw OR 'rafazil':ti,ab,kw OR 'ricordo':ti,ab,kw OR 'sulbenin':ti,ab,kw OR 'tuadin':ti,ab,kw OR 'uxazen':ti,ab,kw OR 'venaxen':ti,ab,kw OR 'yasnal':ti,ab,kw OR 'yasnoro':ti,ab,kw OR 'zakalmer':ti,ab,kw OR 'zauton':ti,ab,kw OR 'zinocept':ti,ab,kw OR 'zopitel':ti,ab,kw).
